# Structural diversity of anti-pancreatic cancer capsimycins identified in mangrove-derived *Streptomyces xiamenensis* 318 and post-modification *via* a novel cytochrome P450 monooxygenase

**DOI:** 10.1038/srep40689

**Published:** 2017-01-18

**Authors:** He-Lin Yu, Shu-Heng Jiang, Xu-Liang Bu, Jia-Hua Wang, Jing-Yi Weng, Xiao-Mei Yang, Kun-Yan He, Zhi-Gang Zhang, Ping Ao, Jun Xu, Min-Juan Xu

**Affiliations:** 1 Ministry of Education Key Laboratory of Systems Biomedicine, Shanghai Center for Systems Biomedicine, Shanghai Jiao Tong University, Shanghai 200240, China; 2 State Key Laboratory of Oncogenes and Related Genes, Shanghai Cancer Institute, Shanghai Jiao Tong University, Shanghai 200240, China; 3 State Key Laboratory of Microbial Metabolism and School of Life Sciences and Biotechnology, Institute of Oceanology, Shanghai Jiao Tong University, Shanghai 200240, China

## Abstract

Polycyclic tetramate macrolactams (PTMs) were identified as distinct secondary metabolites of the mangrove-derived *Streptomyces xiamenensis* 318. Together with three known compounds—ikarugamycin (**1**), capsimycin (**2**) and capsimycin B (**3**)—two new compounds, capsimycin C (**4**) with *trans*-diols and capsimycin D (**5**) with *trans*-configurations at C-13/C-14, have been identified. The absolute configurations of the *tert/tert*-diols moiety was determined in **4** by NMR spectroscopic analysis, CD spectral comparisons and semi-synthetic method. The post-modification mechanism of the carbocyclic ring at C-14/C-13 of compound **1** in the biosynthesis of an important intermediate **3** was investigated. A putative cytochrome P450 superfamily gene, SXIM_40690 (*ikaD*), which was proximally localized to the ikarugamycin biosynthetic pathway, was characterized. *In vivo* gene inactivation and complementation experiment confirmed that IkaD catalysed the epoxide-ring formation reaction and further hydroxylation of ethyl side chain to form capsimycin G (**3′**). Binding affinities and kinetic parameters for the interactions between ikarugamycin (**1**) and capsimycin B (**3**) with IkaD were measured with Surface Plasmon Resonance. The intermediate compound **3′** was isolated and identified as 30-hydroxyl-capsimycin B. The caspimycins **2** and **3**, were transferred to methoxyl derivatives, **6** and **7**, under acidic and heating conditions. Compounds **1–3** exhibited anti-proliferative activities against pancreatic carcinoma with IC_50_ values of 1.30–3.37 μM.

Secondary metabolites from mangrove-derived actinomycetes have been recognized as rich sources of biologically active natural products for pharmaceutical development^1^. *Streptomyces xiamenensis* 318, a moderate halophile isolated from mangrove sediment with a streamlined genome^2^, has been shown to be a cell factory that produces an anti-fibrotic drug candidate, xiamenmycin A. Nineteen gene clusters within the genome were predicted to be involved in secondary metabolism based on the antiSMASH pipeline^2^,^3^. Two biomolecule types, benzopyran^1^ derivatives and polycyclic tetramate macrolactams (PTMs), have been identified as the major secondary metabolites produced by *S. xiamenensis* 318. As a newly discovered PTM-producing strain, *S. xiamenensis* 318 has been subjected to further investigations of its chemical constituents.

PTM compounds are structurally, biosynthetically, and pharmacologically unique natural products^4^,^5^,^6^. To date, there are approximately eleven PTM derivatives with a 5–6–5 ring system, including ikarugamycin (**1**) and capsimycin (**2**), which were previously isolated from *Streptomyces*^7^,^8^,^9^,^10^. The main skeleton consists of a carbocyclic ring system (rings A, B and C) and a 16-membered macrolactam ring (rings D and E)^4^,^5^. The usual post-modifications for rings A-C include epoxidation of the double bond at C-13/C-14 and oxidation of the side chain at C-30^4^,^5^,^6^. The other macrolactam modifications include hydroxylation at C-3 and methylation at N-28^4^,^5^,^6^. The broad diversity of PTM structures triggered our interest in investigating the biosynthetic connections within this group of metabolites. A gene cluster that consisted of *ikaABC* recently proved to be responsible for encoding a hybrid of the PKS and NRPS pathway in ikarugamycin biosynthesis^4^,^5^,^11^,^12^. Gulder *et al*. have demonstrated that the post-PKS/NRPS-hydroxylation at C3 finalizes biotransformation from ikarugamycin to butremycin^13^.

Based on their unique biosynthetic origins, PTMs have multiple biological activities that involve antiprotozoal^14^,^15^, antifungal^7^,^15^,^16^, antibacterial^9^,^17^,^18^ and antiviral actions^19^. They also inhibit cholesteryl ester accumulation in J774 macrophages^14^. Ikarugamycin exhibits particularly pronounced antitumour activities against MCF-7, HMO2, HepG2, Huh7, and HL-60 cells with low IC_50_ values ranging from 1–10 μM^7^,^14^,^20^. Here, we screened the PTMs for their antitumour activities against pancreatic cancer. Pancreatic ductal adenocarcinoma (PDAC) is characterized by frequent metastasis and recurrence over multiple decades, making it one of the deadliest malignancies^21^,^22^. Therefore, the development of new medicine is urgently needed, and bioactive small molecules are becoming increasingly important for therapeutic treatment.

In this study, we carried out a phytochemical analysis of *S. xiamenensis* 318 to pursue new PTMs and profile the secondary metabolites of this strain. The biosynthetic origin and post-modification of capsimycin B (**3**) was of particular interest; therefore, its biosynthetic mechanism was also examined. Furthermore, capsimycin (**2**) and capsimycin B (**3**), can be chemically converted to their *O*-methylation forms *i.e*., capsimycin E (**6**) and F (**7**) by heating under acidic conditions. The bioactivities of ikarugamycin (**1**), capsimycin (**2**) and capsimycin B-F (**3**–**7**) against pancreatic cancer were evaluated *in vitro* using the CCK-8 method, and their cytotoxicities were measured using the lactate dehydrogenase (LDH) method.

## Results and Discussion

### Isolation and structural elucidation of capsimycin derivatives

**Figure 1:**
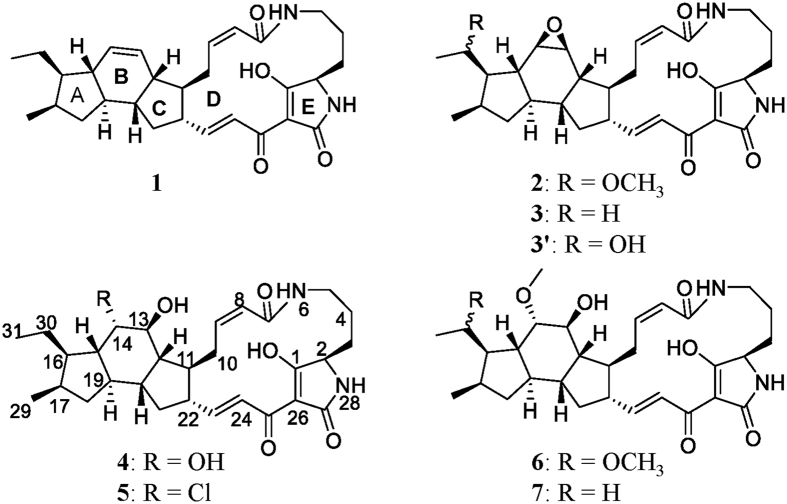
Structures of ikarugamycin (**1**), capsimycin (**2**), capsimycin B (**3**), capsimycin C (**4**), capsimycin D (**5**), capsimycin E (**6**), capsimycin F (**7**), and capsimycin G (**3′**).

Genome mining of a PTM biosynthetic gene cluster along with a chemical analysis of the targeted compound, ikarugamycin (**1**), demonstrated the production of PTMs by *S. xiamenensis* strain 318 in the fermentation broth^2^. In our ongoing phytochemical study of *S. xiamenensis* strain 318, the broth was extracted with ethyl acetate, fractionated by vacuum column liquid chromatography (guided by the ikarugamycin-like UV characteristic with absorption at 325 nm) and separated by semi-preparative HPLC. This targeted approach to chemical profiling resulted in the purification of capsimycin derivatives **2–5** (Fig. 1). Capsimycin (**2**) and ikarugamycin epoxide (**3**), named capsimycin B here, were identified by comparing the HR-MS data with the literature and by an extensive NMR data analysis, respectively. In this paper, we report the structural elucidation of two new capsimyins, C (**4**) and D (**5**).

Compound **4**, named capsimycin C, was isolated as a yellow-beige powder that exhibited an earlier retention time than **3** during separation. Its molecular formula was determined as C_29_H_40_N_2_O_6_ by HRESIMS (m/z 513.2958 [M + H]^+^, calcd 513.2965), which was 18 amu higher than **3**, indicating **4** was the oxidized product of **3**. The ^1^H and ^13^C NMR spectra for **4** (Tables 1 and 2) along with the ^1^H-^1^H COSY and HSQC correlations were similar to those for **3**. The ^1^H NMR spectrum for **4** (Table 1) showed the presence of four olefinic protons [*δ*_H_ 7.03 (1H, d, *J* = 15.4 Hz), 6.75 (1H, dd, *J* = 15.4, 10.3 Hz), 5.99 (1H, dd, *J* = 11.6, 3.1 Hz), and 5.75 (1H, dd, *J* = 11.6, 1.9 Hz)] and two methyl groups [*δ*_H_ 0.81 (3H, d, *J* = 7.0 Hz) and 0.86 (3H, t, *J* = 7.4 Hz)], which represented its structural similarity to **3**, (*i.e*., no double bond in ring B (unlike **1**) and no methoxy group at C-30). The ^13^C NMR and DEPT spectra (Table 2) exhibited 29 signals, indicating the presence of four carbonyl groups (*δ*_C_ 197.5, 175.8, 173.7, and 167.7), four olefinic methines for two double bonds (*δ*_C_ 153.4, 142.3, 123.6, and 122.3), and the remaining 21 carbons (one quaternary carbon at *δ*_C_ 101.0, eleven sp^3^ methines, seven methylenes and two aliphatic methyl groups), in accordance with the main skeleton of **3**. The downfield shifts of the ^1^H and ^13^C NMR signals for C-14 (*δ*_H_ 3.74 and *δ*_C_ 72.1) and C-13 (*δ*_H_ 3.72 and *δ*_C_ 74.6), confirmed by NOE correlations between H-14 and CH_3_-31 at *δ*_H_ 0.86, indicated that the epoxide ring was replaced by diols in **4**. The HMBC also supported the locations of the two hydroxyl groups at C-14 and C-13. Therefore, the planar structure of **4** was determined as 13,14-dihydroxy-ikarugamycin.

The stereoconfigurations of 13,14-diols in compound **4** were determined by semi-synthetic method and ^1^H NMR spectra analysis. The semi-synthetic reaction of 13,14-diol formation *via* S_N_2 mechanism from expoxide compound **3** led to 13,14-*trans*-diol product (**4′**). After purification and comparison of ^1^H NMR spectra between **4′** and **4**, we found out the proton data were identical, indicating the relative configurations of *vic-*diol in **4** were assigned as *trans*. The stereostructure of compound **4** was also determined with NMR spectra analysis and a CD spectra comparison. The strong concordance of the respective NMR data for **4** with those of ikarugamycin, together with the NOESY data and proton coupling constants, indicated that both compounds shared the same relative configuration of ring fusions. Additionally, identical relative configurations at rings A**-**C were shown by NOE correlations between H-15 (*δ*_H_ 1.38, dd, *J* = 11.0, 3.6 Hz), H-20 (*δ*_H_ 1.90, m) and CH_3_-29 (*δ*_H_ 0.81, d, *J* = 7.0 Hz), between H-17 (*δ*_H_ 2.10, m) and H-19 (*δ*_H_ 1.51, m), and between H-22 (*δ*_H_ 2.33, m) and H-12 (*δ*_H_ 1.91, dd, *J* = 10.6, 4.0 Hz). This confirmed the *cis* fusion of the A/B ring, *trans* fusion of the B/C rings and *cis* fusion of the C/D ring, respectively. For the macrolactam ring, the double bond configurations were assigned as 8*Z* and 23*E* from the proton coupling constants (*J*_8,9_ = 11.6 Hz and *J*_23,24_ = 15.4 Hz, respectively). The H-2 configuration, which was originally the *α*-proton of L-ornithine, was predicted to have the *β*-orientation previously reported for clifednamide A^23^.

**Figure 2:**
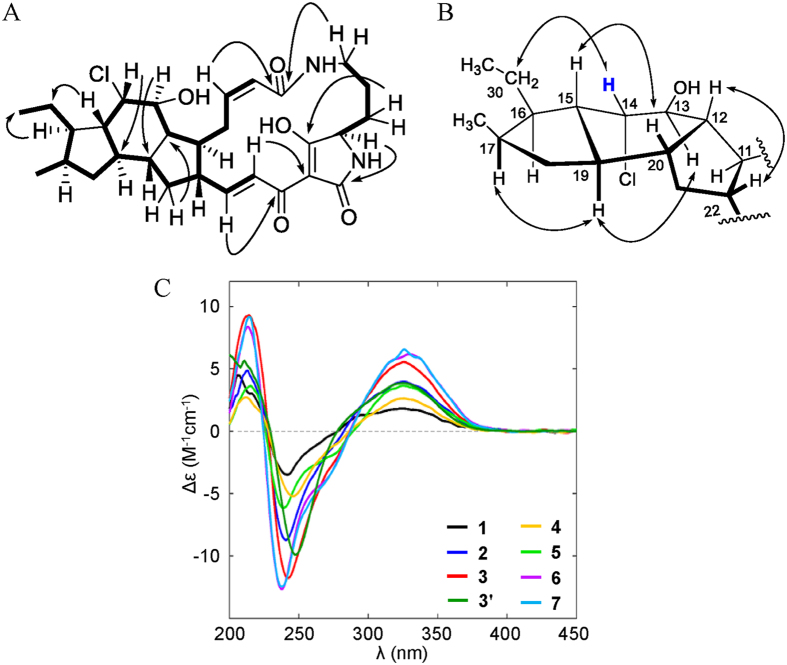
Structural elucidation of **5**. (**A**) Key HMBC and ^1^H-^1^H COSY correlations of capsimycin D (**5**). (**B**) Key NOESY correlations for capsimycin D (**5**). (**C**) ECD curves for compounds **1–7**.

A chloride-containing capsimycin C (**5**) was isolated from *S. xiamenensis* and became the first representative of a new halogenated PTM subtype. With an ionized molecular peak at m/z 531.2623, compound **5** showed an isotopic peak at m/z 533.2637 with a high relative intensity of 3:1 in its HR-ESI-MS spectrum, indicating the halogenated substituent compound. The molecular formula for **5** was determined as C_29_H_39_ClN_2_O_5_ and was further confirmed by electrospray ionization-fourier transform-ion cyclotron resonance-mass spectrometry (ESI-FT-ICR-MS) at m/z 531.26215, which was 36 amu higher than **3**. Like **3**, the ^1^H NMR spectrum for **5** (Table 1) showed the presence of four olefinic protons [*δ*_H_ 7.13 (1H, d, *J* = 15.4 Hz), 6.83 (1 H, dd, *J* = 15.4, 10.3 Hz), 6.06 (1H, ddd, *J* = 11.5, 11.5, 3.4 Hz), and 5.84 (1H, dd, *J* = 11.5, 1.3 Hz)] and two methyl groups [*δ*_H_ 0.90 (3H, d, *J* = 6.8 Hz) and 0.94 (3H, t, *J* = 7.4 Hz)]. The ^13^C NMR and DEPT spectra (Table 2) were in close agreement with those for **3**. The HMQC spectrum allowed assignments for all protons to their corresponding carbon atoms. Together with the HMBC correlation analysis, the ^1^H and ^13^C NMR data revealed the gross structure for **5**, which was a derivative of compound **3**. The main difference between **5** and **3** occurred in ring B, where the epoxide ring at C-13/C-14 was opened and substituted by a hydroxylated group and one chlorine atom to produce the carbons at *δ*_C_ 73.7 (d, C-13) and *δ*_C_ 64.7 (d, C-14), respectively. The HMBC correlations between H-12 (*δ*_H_ 2.07, m) and C-14, C-13 and C-20 (*δ*_C_ 42.6, d) and between H-15 (*δ*_H_ 1.77, m) and C-14, C-17 (32.6, d) and C-20 confirmed this substitution (Fig. 2A). Therefore, the structure of **5** was determined as 14-chloride-13-hydroxy-ikarugamycin.

The stereostructure of **5** was determined from the NMR and CD data. To confirm that the relative configurations of the A-D ring junctions in **5** were identical to those in **1**, ROESY correlations were observed between H-15, H-20 (*δ*_H_ 2.07, m) and CH_3_-29 (*δ*_H_ 0.90, d, *J* = 6.8 Hz) and between H-17 (*δ*_H_ 2.21, m) and H-19 (*δ*_H_ 1.61, m), together with H-22 (*δ*_H_ 2.41, m) and H-12. Their similar proton coupling constants (*J*_8,9_ = 11.5 Hz and *J*_23,24_ = 15.4 Hz) showed a 8*Z*, 23*E* double bond in the marolactam ring. The NOE correlation in ring B between H-13 (*δ*_H_ 4.13, br t, *J* = 2.6 Hz) and H-19 indicated the *β*-orientated proton at C-13, but the correlation between H-14 (*δ*_H_ 4.23, br t, *J* = 2.9 Hz) and H_2_-30 (*δ*_H_ 1.35, m) indicated the *α*-orientated proton at C-14, revealing the relative configurations of H-13 and H-14 as *trans* (Fig. 2B). The absolute configurations of **4** and **5** have been determined by comparing their ECD curves with those of ikarugamycin (**1**) and capsimycin (**2**) (Fig. 2C), whose absolute configurations were previously confirmed by oxidative degradation^8^, total synthesis^24^ and X-ray crystallography^25^. Based on its optical rotation values, the capsimycin analysed in this study had an identical configuration to the previously established one [capsimycin: 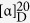 + 164.6 (c 0.35, chloroform) vs. +196° (c 1, chloroform)^10^].

Compound **3′** was isolated from *S. xiamenensis* as a key intermediate with an ionized molecular peak at *m/z* 511.2803 (calcd 511.2808), which was 14 amu less than **2**. The molecular formula for **3′** was determined as C_29_H_38_N_2_O_6_, indicating one methyl group was missing in **3′** compared with **2**. The ^1^H NMR spectrum for **3′** also showed the presence of four olefinic protons [*δ*_H_ 7.28 (1H, m), 6.68 (1H, dd, *J* = 15.0, 9.8 Hz), 6.05 (1H, ddd, *J* = 11.9, 10.1, 2.8 Hz), and 5.91 (1 H, d, *J* = 11.9 Hz)] and two methyl groups [*δ*_H_ 1.36 (3 H, d, *J* = 6.3 Hz) and 1.08 (3 H, d, *J* = 7.7 Hz)]. The ^13^C NMR and DEPT spectra were also in close agreement with those for **2**, except for one methoxy group missing in **3′**. The main difference between **3′** and **2** occurred in ethyl side chain at ring A, where the methoxy group at C-30 was substituted by a hydroxyl group. Together with the HMQC and HMBC correlation analysis, the ^1^H and ^13^C NMR data revealed **3′** was a demethylation form of compound **2**. Therefore, the structure of **3′** was determined as 30-hydroxy-capsimycin B and named as capsimycin G.

### Epoxidation and hydroxylation *via* a cytochrome P450 monooxygenase IkaD leading to the biosynthesis of capsimycin

**Figure 3:**
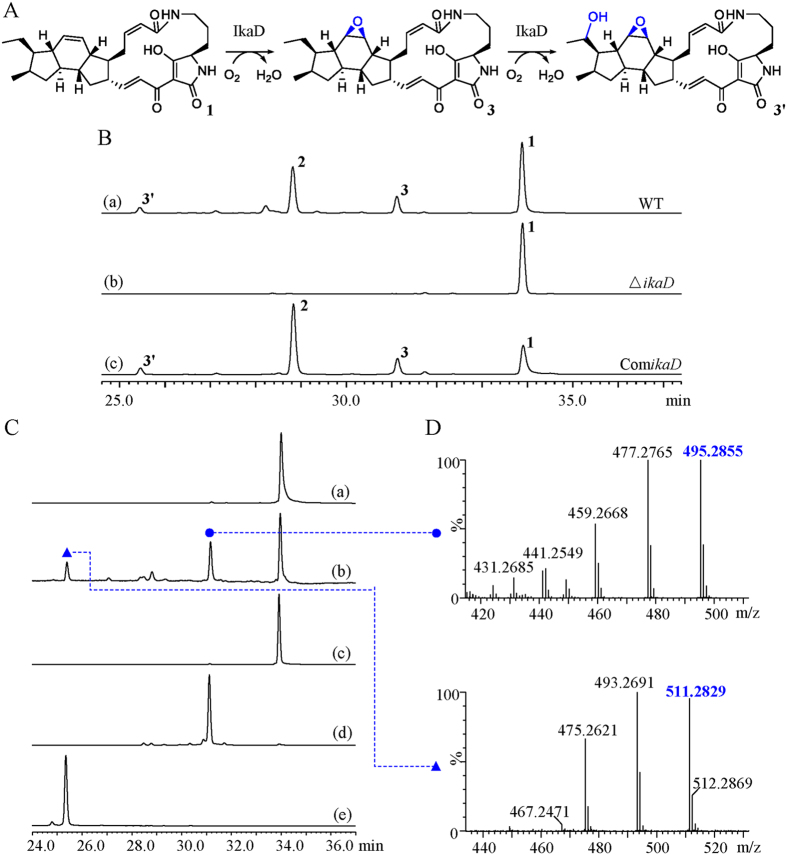
Epoxidation and hydroxylation *via* a cytochrome P450 monooxygenase IkaD leading to the biosynthesis of capsimycin. (**A**) Biochemical reactions showing the conversion of ikarugamycin (**1**) to capsimycin B (**3**), and then to compound **3′**. (**B**) Mutational analysis of the *ikaD* gene. HPLC analysis for metabolic profile for (a) Wild type (WT); (b) Disruption of *ikaD gene* abolished the production of **3** and **3′** (∆*ikaD*); (c) Complementation of *ikaD* gene restored the production of **3** and **3′** (Com*ikaD*). Analytical HPLC were performed on a HPLC system with a ZORBAX Extend-C18 column (4.6 × 150 mm, 5 μm, Agilent) under a gradient of 20%-100% MeOH/H_2_O over 40 min (1 mL/min) and monitored by UV detection at 325 nm. (**C**) HPLC analysis of the *in vitro* assay of IkaD and monitored by UV detection at 325 nm. (a) heat-inactivated IkaD incubated with ikarugamycin (**1**) as negative control; (b) IkaD incubated with ikarugamycin (**1**); (c) a standard of ikarugamycin (**1**); (d) a standard of capsimycin B (**3**); (e) a standard of compound **3′**. (**D**) UPLC-MS/MS spectra of capsimycin B (**3**) (upper) and compound **3′** (below) in the positive ionization mode.

Based on the increasing structural complexities revealed by our study, compounds **1**, **3** and **2** were fitted into a hypothetical biosynthetic grid to provide insights into the ikarugamycin post-modification process. This revealed capsimycin B (**3**) as an important intermediate within the biosynthetic route. The initial oxidation on the B ring was predicted to occur at the double bond of **1** to form an epoxide ring, as observed in **3** (Fig. 3A). Compound **3** would be further oxidized at C-30 of the side chain to produce 30-hydroxylation intermediate (**3′**) and *O*-methylation derivative (**2**).

**Figure 4:**
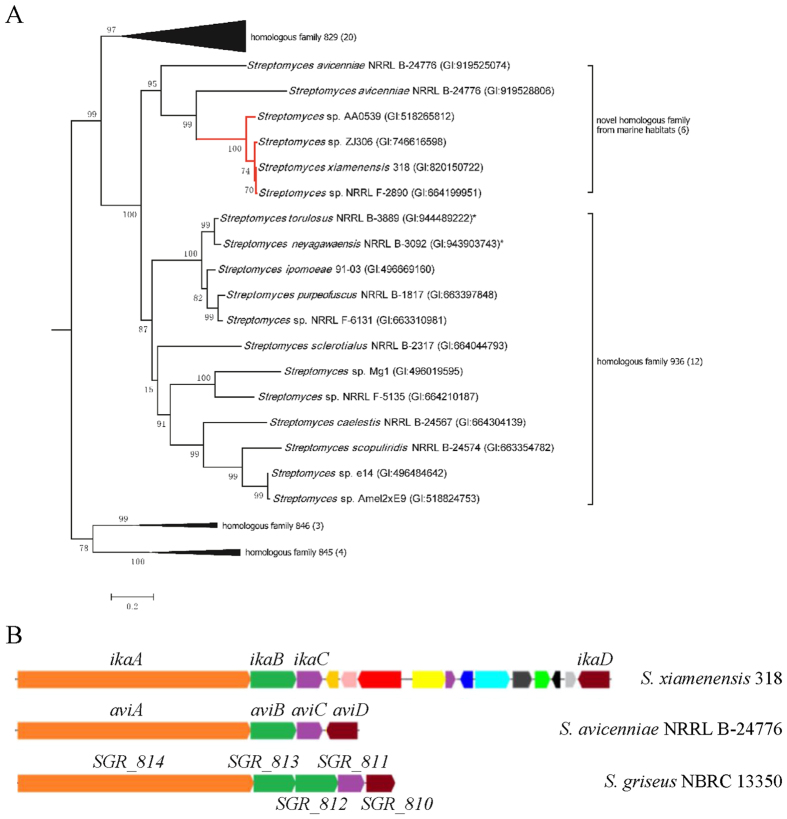
Phylogenetic analysis of the novel cytochrome P450 monooxygenase IkaD and schematic diagram of the gene clusters encoding PTM. (**A**) The Maximum-likelihood phylogenetic tree was rooted using a CYP102-clan of P450 superfamily member (GI:490075884 from *S. coelicolor*) and only the interested part of the dendrogram was shown (see Supplementary Fig. S3 for full dendrogram). Bootstrap values (expressed as percentages of 1000 replications) are shown at branching points. Bar, 0.02 substitutions per nucleotide position. *two of the top hits identified in the BlastP analysis against GenBank using SXIM_40690 but not listed in the CYP107-clan of P450 superfamily in CYPED. Numbers in parenthesis stand for the collapsed lineages of the specific homologous family. (**B**) PTM in *S. xiamenensis* 318 (*ikaA*, GI:820156332; *ikaB*, GI:921170534; *ikaC*, GI:664199966; *ikaD*, GI:820150722); PTM in *S. avicenniae* NRRL B-24776 (*aviA*, GI:919525077; *aviB*, GI:919525076; *aviC*, GI:919525075; *aviD*, GI:919525074); PTM in *S. griseus* NBRC 13350 (SGR_814, GI:178463123; SGR_813, GI:178463122; SGR_812, GI:178463121; SGR_811, GI:178463120; SGR_810, GI:178463119).

Driven by this hypothesis, a putative cytochrome P450 superfamily gene, SXIM_40690, was pursued downstream of the characterized ikarugamycin biosynthetic gene cluster in *S. xiamenensis* 318 and named as *ikaD* (Fig. 4B). A BlastP analysis using IkaD as the query against GenBank showed that three nearly identical amino acid sequences (>90% identity, 100% coverage) were found in *Streptomyces* sp. ZJ306, *Streptomyces* sp. NRRL F-2890 and *Streptomyces* sp. AA0539. As shown previously, these three strains had identical 16S rRNA gene sequences and were tentatively assigned to *S. xiamenensis*^2^. Moreover, counterparts of the characterized ikarugamycin gene cluster that consisted of *ikaA, ikaB* and *ikaC* were found in this group of *S. xiamenensis* strains (Supplementary Table S1).

All other protein homologues of IkaD were from actinomyces, but their similarity values fell sharply (ranging from 43% to 59%). The nomenclature of the diversified cytochrome P450 proteins indicated that the amino acid sequence identity values of 40% and 55% were the suggested cut-off values for assigning P450 homologues into an established family or subfamily^26^,^27^. IkaD had conserved cytochrome P450 protein domains (Supplementary Figure S2) and fell into the CYP107 superfamily category in the CYPED database (https://cyped.biocatnet.de/sFam/107). Phylogenetic analyses were performed using the top hits retrieved by the BlastP search in CYPED (Supplementary Figure S3). Along with its close relatives, IkaD from *S. xiamenensis* formed a small clan with two homologue proteins from *S. avicenniae* (59% for gi|919528806|ref|WP_052851163.1 and 53% for gi|919525074|ref|WP_052848984.1, respectively). Surprisingly, a *S. avicenniae* CYP homologous gene counterpart was also localized to a putative PTM gene cluster (Fig. 4B). Interestingly, both *S. xiamenensis* and *S. avicenniae* were described as novel species that originated from the same mangrove habitat^28^,^29^. The clear separation of the IkaD clan in the tree from the so-called CYP107 subfamily-936 clan suggests that IkaD can be considered a novel cytochrome P450 monooxygenase (Fig. 4A and Supplementary Figure S3).

As shown in Fig. 3B, the biosynthesis of compounds **2**, **3** and **3′** in the *ikaD* disruptant mutant was abolished. Complementation of *ikaD* in a plasmid clearly restored the production of compounds **2**, **3** and **3′**, suggesting *ikaD* related to the generation of compounds **2**, **3** and **3′**. IkaD was heterologously expressed in *E. coli* and the purified protein was digested by Trypsin and then subjected to nano LC-MS/MS analysis. Collected peptide sequences were searched against the Trembl Bacteria database using Mascot program. IkaD was identified as the unspecific monooxygenase in *S. xiamenensis* with the gene locus SXIM_40690 (significance threshold *p* < 0.05, see Figure S80). Size exclusion chromatography analysis showed that expressed IkaD exists as a monomer (see Figure S79).

IkaD was incubated with compound **1**, together with 100 μg/mL spinach ferredoxin, 0.2 U/mL spinach ferredoxin-NADP^+^ reductase and 1 mM NADPH in 50 mM Tris-HCl, pH 7.5. This assay resulted in the biotransformation from **1** to **3**, as the products compared with authentic standard sample by UPLC-QTOF-MS. The *in vitro* biotransformation of ikarugamycin to **3** was shown using IkaD as the biocatalyst (Fig. 3C). In the oxidative cascade that precedes further hydroxylation of **3**, dioxidized compound **3′** was observed and identified afterwards by NMR spectra analysis (Supplementary Table S9). The dual function, including the epoxidation from **1** to **3** and further hydroxylation from **3** to **3′** by IkaD, suggested that this versatile P450 monooxygenase should be considered in the enhancement of PTM structural diversity. Epoxides are the products of P450 monooxygenase activity conjugated on double bond, leading to potentially toxic intermediate, such as **3**, which can react with nucleophilic sites in DNA and proteins^30^. Thus, the functional elucidation of the tailoring enzyme involved in ikarugamycin biosynthesis makes precise biosynthesis, with the aim of producing a single molecule type, possible. This will be important for a bioactivity evaluation that specifically addresses the relationship between structure and bioactivity.

### Decomposition and oxidation of capsimycins *via* chemical reaction

**Figure 5:**
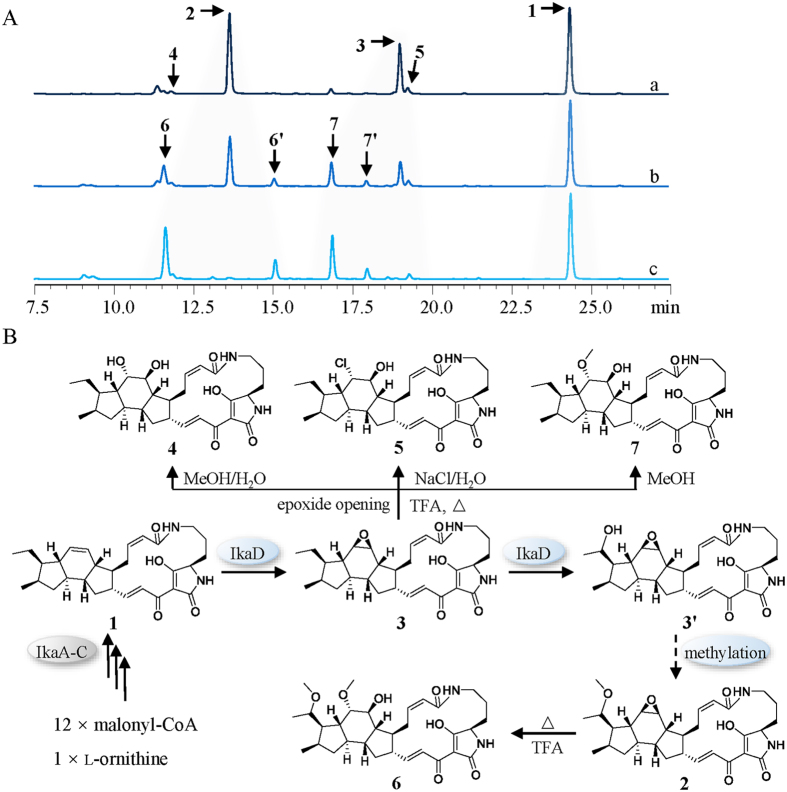
Transformation scheme of **1**–**7**. (**A**) HPLC analysis of the changes of **2** and **3** in methanol solution (contained 1‰ TFA) at 45 °C for (a) 0 min, (b) 60 min, (c) 120 min, while ikarugamycin (**1**) is unchanged under same condition. (**B**) Proposed (bio)transformation of **1**–**7.**

During the chemical analysis of *S. xiamenensis* strain 318, we discovered that different extraction methods may lead to differing metabolic profiles with missing peaks for **2** and **3**, the two major metabolites obtained under the acidic and high temperature conditions used during rotoevaporation (Fig. 5A). Two possible oxidative products (**6** and **7**) were deduced through a molecular weight analysis after methanol solutions for **2** and **3** were treated with strong protonic acids, such as trifluoroacetic acid (1‰, vol), at 40–60 °C (Fig. 5A and Supplementary Figure S5). These two acidification products were separated from their original substrates by preparative HPLC with a solvent system consisting of (A) H_2_O (0.5‰ trifluoroacetic acid) and (B) MeOH (0.5‰ trifluoroacetic acid). However, when we used a weak acid (*i.e*., formic acid) as the inducer, the reactions were slower and more inefficient (less than one-tenth of the substrates were acidized with 5‰ formic acid in 2 h). Furthermore, a weaker acid (*i.e.,* acetic acid) did not induce any acidification. To elucidate the *in vitro* chemical conversion process, we performed UPLC-MS profiling, followed by an isolation and structural elucidation of compounds **6** and **7**.

The HR-MS result for **6** (capsimycin E, *m/z* 557.3235 [M + H]^+^, C_31_H_45_N_2_O_7_, calcd 557.3227) indicated the methoxy derivative of **2**. The ^1^H and ^13^C NMR data for **6** were in close agreement with those for **2** (Tables 1 and 2), with the exception of an additional methoxy group evidenced by NMR signals at *δ*_H_ 3.42 (3H, s) and *δ*_C_ 58.6 (q). This substituent was deduced to replace the hydroxy group at C-14 (*δ*_C_ 83.0), based on the HMBC correlations between the methoxy protons. Accordingly, the planar structure for **6** was identified as 14-methoxy-capsimycin The ROESY correlations for H-13/H-19 and H-14/H-12 indicated that the relative configuration of the stereocentres at C-13 and C-14 in **6** was *trans*.

The molecular formula for **7**, named capsimycin F, was determined to be C_30_H_42_N_2_O_6_ by HRESIMS (*m/z* 527.3104 [M + H]^+^, calcd 527.3121), which was 32 amu higher than that of **3**. A comparison of the ^1^H and ^13^C NMR data (Tables 1 and 2) along with the 2D NMR data (COSY, HMQC, HMBC, and NOESY) revealed that the A-to-D ring skeleton for **7** was identical to that for **3**. An additional methoxy group signal at *δ*_H_ 3.42 (3H, s) and *δ*_C_ 59.0 (q) appeared to be the major difference between **7** and **3**. As with **6**, this methoxy group was deduced to substitute the C-14 hydroxy group, based on the HMBC correlations between the methoxy protons and C-14 (*δ*_C_ 82.5, d). Accordingly, the planar structure for **7** was identified as 14-methoxy-capsimycin B. The relative configurations of the two stereocentres in ring B of **7**, C-13 and C-14, were determined as *trans* by the ROESY correlations for H-13/H-19 and H-14/H_2_-30.

Therefore, the epoxide ring at C-13/C-14 proved to be chemically unstable under acidic conditions at 40–60 °C and yielded the ring-opened and *O*-methylated products (**6** and **7**). Additionally, there were two other compounds **6′** and **7′**, isomers of **6** and **7** respectively, found in extracted ion chromatography by UPLC-MS (Supplementary Figure S6). Possibly, due to stereo-hindrance of the macrolactam ring, most *O*-methylation occurred at C-14, making **6** and **7** the major products. More derivatives originated from **3** could be found based on UPLC-HR-ESI-MS. In the solvent system of MeOH/H_2_O, **3** can be transferred to **4**. When NaCl/NaBr/NaI was added to MeOH/H_2_O, **3** can be transferred to **5** or other halogenated derivatives under either the acidic or heating condition. It indicated that compounds **4** and **5** might be an artifact during the extraction and isolation.

### Capsimycin fragmentation patterns by UPLC-QTOF-MS/MS

The MS/MS fragmentation patterns for PTMs (**1–7**) were examined as a convenient method for the fast detection of new metabolites and for de-replication of the known ones^31^. The PTM chemical structure is composed of two major moieties (the carbocyclic and macrolactam rings) and post-modifications, including methoxylation of the C**-**30 side chain. In principle, sufficiently abundant positive [M + H]^+^ ions can be used in MS/MS experiments to provide two types of structural information based on different C-30 substitutions. The fragmentation pattern analyses of **1–7** are important for characterizing metabolites that are present in trace amounts. Accurate mass measurements of the pseudo-molecular ions were listed in Supplementary Table S2.

**Figure 6:**
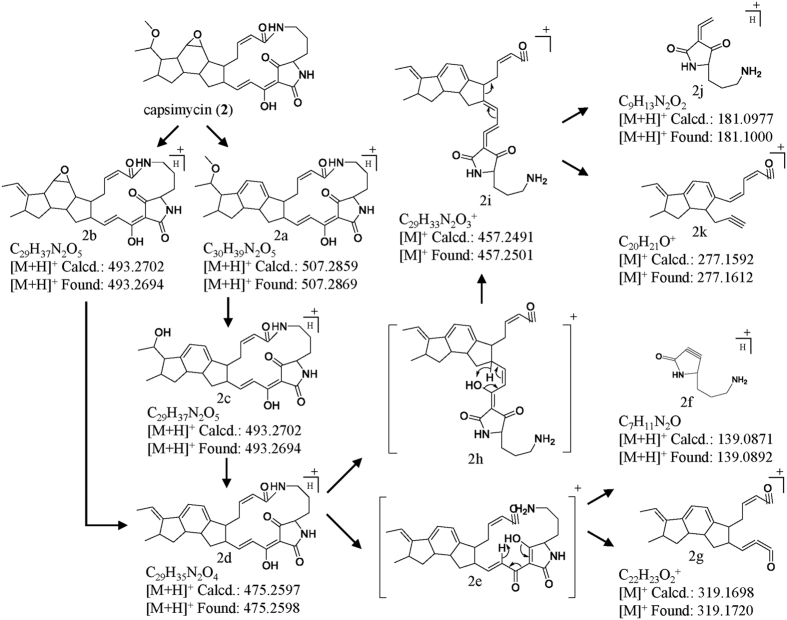
(+) ESI-MS/MS fragmentations of **2** by ionization of [M + H]^+^ and proposed mechanism.

The capsimycin (**2**) daughter ions (*m/z* 507.2869, 493.2694, 475.2598, 457.2501, 277.1612, 181.1000 and 139.0892) appeared under high collision energy (Fig. 6). The highest abundant fragment ion (*m/z* 493.2694) formed by a loss of −MeOH (*m/z* 32), possibly through the loss of a methoxyl group, possibly at C-30, to subsequently form a double bond. The *m/z* 475.2598 molecular ion was produced by the sequential opening of the epoxide ring and dehydration by loss of –H_2_O. The additional fragment ions (*m/z* 457.2501) were formed by cleavage of the amide bond in the macrolactam ring, followed by McLafferty rearrangement (Fig. 6). The *m/z* 277.1612 and 181.1000 fragment ions, produced from the second daughter ions at 457.2501, were formed by rearrangement and cleavage. Compound **6** is the methyl ester of capsimycin (**2**) at C-14, and both compounds had similar mass fragment patterns and similar relative abundance ratios (Supplementary Table S2 and Figure S7). Therefore, capsimycin (**2**) and capsimycin E (**6**) have identical fragmentation patterns.

Based on the fragmentation processes proposed for **2** and **6**, fragment ions at *m/z* 477.2761, 459.2661, 279.1766, 181.0993 and 139.0885 were pursued in the MS/MS spectrum for **3**. Compound **3** had a macrolactam ring cleavage fragmentation at *m/z* 181.0993 and 139.0885, which was identical to capsimycin (**2**). However, the highest abundant fragment for **3** was *m/z* 477.2761, and the *m/z* 459.2661 and 279.1766 fragments were 2 amu higher than those for **2** (Supplementary Table S2). These were probably formed by the side chain replacement at C-30, which would lead to a loss of one degree of unsaturation for each fragment. Compounds **4**, **5**, and **7** exhibited similar fragmentation patterns to that of compound **3** due to their identical C-30 side chains (Supplementary Figure S7).

The minor amount biosynthetic intermediate, dioxidized **3′**, was firstly found based on the fragmentation pattern and then further isolated and identified by NMR data analysis. The molecular formula for **3′** was determined by UPLC-QTOF-MS (*m/z* 511.2825) to be C_29_H_38_N_2_O_6_, which was 16 amu higher than **3**, indicating a hydrolysed product for **3**. The molecular ions observed at *m/z* 475.2615 and 277.1656 on the MS/MS fragmentation pattern for **3′** were consistent with those of **2** and **6**, which proved to possess the same PTM skeleton. Thus we showed that further hydroxylation occurs at C-30. Therefore, we can clarify the roles of IkaD in C-13/C-14 epoxidation and C-30 hydroxylation.

### Evaluation of bioactivity and cytotoxicity of compounds 1–7

**Figure 7:**
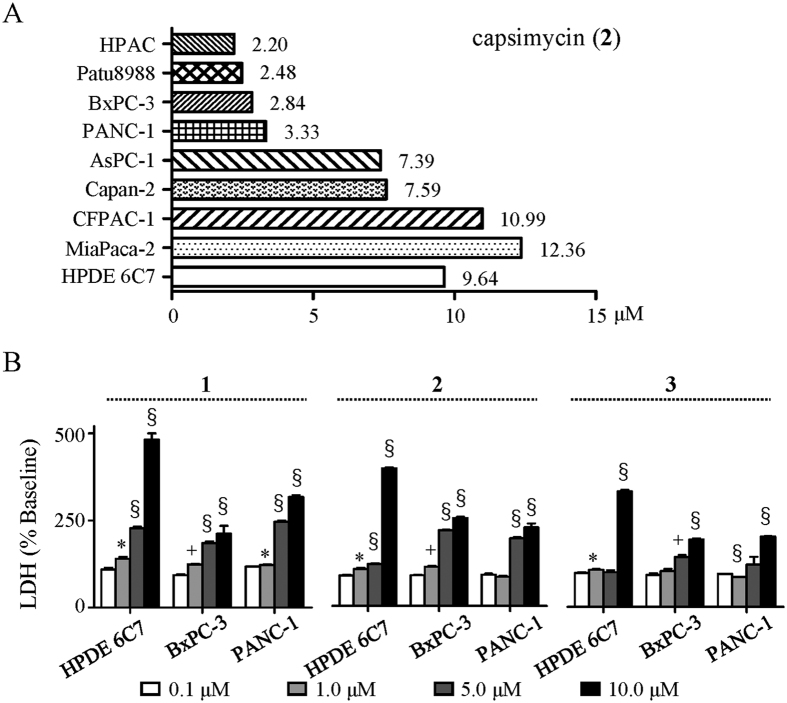
Bioactivities of 1–3 against pancreatic cancer. (**A**) IC_50_ of **2** against 8 pancreatic cell lines and one normal cell line HPDE-6C7. (**B**) Cytotoxicity analysis with LDH method in BxPC-3 and PANC-1, and normal cell line HPDE-6C7 after treatment with compounds **1**–**3** (Student’s T-test, n = 3, *P < 0.05, ^+^*P* < 0.01, ^§^*P* < 0.001, values differ from 0.1 μM compounds at indicated cell type).

Given the high antitumour activities exhibited by PTMs, compounds **1–7** were investigated for their anti-proliferation bioactivities against pancreatic cancer. Ikarugamycin (**1**), capsimycin (**2**), and capsimycin B (**3**) showed strong anti-proliferation activities (IC_50_ = 1.30, 3.33, and 3.37 μM, respectively). Compounds **2** and **3**, each possessing an epoxide ring at C-13/C-14 instead of a double bond, showed higher IC_50_ values compared to **1**. In addition, compound **2** were extensively screened for its bioactivity of anti-pancreatic cancer cell lines, including eight pancreatic cancer cells (HPAC, Patu8988, BxPC-3, PANC-1, AsPC-1, Capan-2, CFPAC-1, and MiaPaca-2) and one normal cell (HPDE-6C7). All the proliferations of pancreatic cancer cell lines were inhibited by compound **2**, while the IC_50_ values of six (HPAC, Patu8988, BxPC-3, PANC-1, AsPC-1, and Capan-2) were below 9.64 μM, an IC_50_ value against HPDE-6C7 (Fig. 7A). Furthermore, compounds **4–7**, which were the final products of the sequential oxidative reaction, were significantly less active than **1–3** as ascertained by a pronounced reduction in inhibition ratio at 10 μM. This emphasizes the importance of the C-13/C-14 double bond and epoxide ring for investigations of structure-activity relationships and suggests a possible mechanism for detoxification.

The cytotoxicity against normal human pancreatic ductal cell (HPDE-6C7) was evaluated using the LDH method. At the higher concentration of 10 μM, compounds **1–3** showed cytotoxic activities against HPDE6-C7, but these toxicities were weaker throughout their IC_50_ value ranges (Fig. 7B). This provided a reasonable therapeutic window for further pharmaceutical development. Therefore, **1–3** induced cell death in PANC-1 with IC_50_ values within the 1.30–3.37 μM range but exhibited negligible cytotoxicities towards HPDE-6C7 cell at the same concentrations.

## Experimental section

### Materials

All chemicals and reagents used for biochemical and molecular assays were purchased from standard commercial sources. The PrimeSTAR Max DNA Polymerase and pET vector were purchased from TaKaRa (Dalian, China), while restriction enzymes and T4 DNA ligase were purchased from Thermo Fisher Scientific (Waltham, MA, USA).

### Phylogenetic analysis of the cytochrome P450 monooxygenase

Amino acid sequences used in the phylogenetic analysis were downloaded from NCBI and the CYtochrome P450 Engineering Database (CYPED, https://cyped.biocatnet.de/). Sequences were aligned using ClustalW and manually degapped. The maximum-likelihood tree was built using FastTree (http://www.microbesonline.org/fasttree/) and included 430 representative amino acid sequences in the CYP107-clan and 6 CYP107-clan-like amino acid sequences. The tree was rooted using a CYP102-clan of a P450 superfamily member (GI:490075884 from *S. coelicolor*).

### DNA manipulation, cloning, and PCR

Using the whole genome of *S. xiamenensis* 318 as the template, the *ikaD* gene was amplified by PCR under standard conditions with the following primers: forward, 5′**-**CGGGATCCGATGCCCGGACAGCAGGAACA-3′ (introduced a *Bam*HI site); reverse, 5′-CCGCTCGAGCCAGGCGACGGGCAGTTCGT-3′ (introduced a *Xho*I site without a stop codon). The amplified DNA fragment was digested with *Bam*HI and *Xho*I and was ligated into pET24b via *Bam*HI/*Xho*I to generate the pET24b-*ikaD* recombinant plasmid for the expression of C-terminal His-tagged IkaD. After confirming the identities of the inserted genes by DNA sequencing, the constructs were used to transform *E. coli* Rosetta (DE3) for protein overexpression.

### Inactivation and complementation of *ikaD* gene

The 1722-bp left flanking region of *ikaD* was amplified by using primers 5′**-**GCTCTAGATACCTCAATGTGCCGCTGCT-3′ (introduced a *Xba*I site) and 5′**-**GGAATTCGGGGGCGGTGTTGCCTTTCG-3′ (introduced a *EcoR*I site). The 1743-bp right flanking region of *ikaD* was amplified by using primers 5′**-**GGAATTCCCACGAGAAGTGGCCTGTG-3′ (introduced a *EcoR*I site) and 5′**-**CCCAAGCTTAGATCAAGCTCAGCACACCC-3′ (introduced a *Hind*III site). After digestion of the amplified left and right PCR fragments, both fragments were ligated with *Xba*I/*Hind*III digested pJTU1278^32^, an *E. coli*-*Streptomyces* shuttle vector, to generate p40690. The plasmid p40690 was introduced into *S. xiamenensis* 318 by intergeneric conjugation, and thiostrepton-resistant (ThioR) single-crossover exconjugants were selected. Second-crossover events were expected after a round of non-selective growth of the initial ThioR exconjugants. Marker-free deletion mutant ∆*ikaD* was screened by a diagnostic PCR and the removal of the whole *ikaD* was confirmed by DNA sequencing of the mutant gene locus in ∆*ikaD*. To complement the *ikaD* mutation, a plasmid pCom*ikaD*, in which the complete *ikaD* gene under the control of a strong promoter PermE, was constructed. The gene *ikaD* was amplified from *S. xiamenensis* 318 genome using the primers 5′**-**GGAATTCCATATGATGCCCGGACAGCAGGAACA-3′ (introduced a *Nde*I site) and 5′**-**CGGAATTCCTACCAGGCGACGGGCAGTTCGT-3′ (introduced a *Eco*RI site). The PCR product was purified and digested with *Nde*I and *Ec*oRI, and then ligated into *Nde*I/*EcoR*I-digested pIB139^33^, to generate plasmid pCom*ikaD*. The pCom*ikaD* was introduced into ∆i*kaD* through conjugation, and an apramycin-resistant (AmR) exconjugant was selected and named Com*ikaD*.

### Overexpression and purification of IkaD

The *E. coli* Rosetta (DE3) cells were grown at 37 °C in 500 ml of LB broth containing 34 μg/ml chloramphenicol and 50 μg/ml kanamycin. When the OD_600_ reached 0.6–0.8, IPTG (isopropyl β-D-thiogalactoside) was added to a final concentration of 0.5 mM, and the cells were cultured at 16 °C overnight. Cells were harvested by centrifugation, resuspended in binding buffer (20 mM Tris-HCl, pH 8.0, 300 mM NaCl, 10% glycerol and 10 mM imidazole) and lysed by high pressure homogenisation (ATS engineering). The insoluble material was separated by centrifugation (10,000 rpm for 30 min at 4 °C), and the soluble fraction was incubated with 2 ml Ni-NTA resin in a column. The column was washed with 30–50 ml of wash buffer (20 mM imidazole in binding buffer), and elution buffer (300 mM imidazole in binding buffer) was subsequently added into the column. The protein fractions were pooled and dialysed against the storage buffer (50 mM Tris-HCl, pH 7.5, 200 mM NaCl, 0.2 mM dithiothreitol and 20% glycerol) at 4 °C overnight. The presence of purified IkaD was assessed by SDS-PAGE (12% gels), and the concentration was determined using the Bradford method with BSA as the standard^34^.

### *In vitro* conversion of ikarugamycin into capsimycin B by IkaD

A standard conversion was achieved by combining 0.5 μM IkaD, 140 μM ikarugamycin, 100 μg/mL spinach ferredoxin, 0.2 U/mL spinach ferredoxin-NADP^ + ^reductase and 1 mM NADPH in 50 mM Tris-HCl, pH 7.5. A reaction with boiled enzyme was used as a negative control. The reaction was carried out for 1 h at 30 °C and terminated by adding 3 × 200 μL of EtOAc. The resulting organic extraction was dried and dissolved in 100 μL methanol for the HPLC and UPLC-MS analyses.

### General chemical experimental procedures

Optical rotations were measured with an Autopol V plus polarimeter (Rudolph). A Shimadzu UV-1800 spectrophotometer was used to record the UV spectra. The IR spectra were determined using a Nicolet 6700 FT-IR spectrometer. The CD spectra were obtained using a J-815 spectrometer (JASCO) at 20 °C. ^1^H, ^13^C and 2D NMR spectra were acquired with an Avance III 600 MHz NMR spectrometer (Bruker), and TMS was used as the internal standard. Semipreparative HPLC was performed using a PC-2000 instrument (Laballiance) with a Kromasil C18 column (10 × 250 mm, 5 μm, AkzoNobel). FT mass spectra were recorded using a solariX 7T FT-ICR mass spectrometer (Bruker). The ESIMS and MSMS spectra were acquired using an ACQUITY UPLC system equipped with a Micromass Q-TOF Premier mass spectrometer (Waters), and UPLC-QTOF-MS and MSMS methods were previously described^35^. Analytical HPLC were performed on a Prominence module HPLC system (Shimadzu) with a Kromasil 100-5C8 column (4.6 × 250 mm, 5 μm, AkzoNobel) and a ZORBAX Extend-C18 column (4.6 × 150 mm, 5 μm, Agilent) under a step gradient of 40–100% CH_3_CN/H_2_O over 35 min (1.2 mL/min) and 20–100% MeOH/H_2_O over 40 min (1 mL/min) at UV 190–800 nm, respectively.

### Fermentation, extraction and isolation

Seed cultures of *Streptomyces xiamenensis* strain 318 were grown in Tryptone Soy Broth (TSB) medium (Oxoid) at 30 °C for 40 h in a rotary shaker at 200 rpm. The seed cultures were subsequently transferred into 100 × 100 mL of ISP2 medium (Glucose 4 g/L, Yeast extract 4 g/L, Malt extract 10 g/L, pH = 7.2) at 3% (v/v) in 500 mL Erlenmeyer flasks and cultivated in a rotary shaker at 30 °C for 7 days. The liquid cultures were extracted three times with equal volumes of EtOAc. The organic layers were combined and enriched *in vacuum* to obtain 7 g of the dark brown crude extract. Half of the extract was subjected to ODS column chromatography using a step gradient elution of MeOH/H_2_O (3:7 to 9:1, v/v) to produce five major fractions (Fr-1 to Fr-5). Fr-3 was purified by semipreparative reversed-phase HPLC using 70% CH_3_CN (0.5‰ formic acid) to yield **2** (25 mg) and **3**′ (3 mg). Fr-4 was further separated using semipreparative HPLC under a gradient solvent system from 60% to 90% CH_3_CN to yield **3** (7 mg), **4** (2.6 mg), and **5** (5.5 mg). Fr-5 was also purified by HPLC to produce ikarugamycin (**1**) (40 mg). The other half of the extract was treated in MeOH solution contained 1‰ TFA at 45 °C for 2 hours, and then purified as the above methods to obtain **6** (12 mg) and **7** (3 mg).

Capsimycin (**2**): yellow-beige powder; 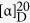 + 164.6 (c 0.35, CHCl_3_); UV (MeOH) λ_max_ (log ε) 323 (3.78); CD (c 1.63 mM, MeOH) λ_max_ (Δε) 213 (+4.87), 241 (−8.71), 326 (+3.98); IR (KBr) ν_max_ 3444, 2931, 1645, 1581, 1433, 1383, 1098, 754 cm^−1^; ^1^H and ^13^C NMR data, Tables 1 and 2; HRESIMS m/z 525.2975 [M+H]^+^ (calcd for C_30_H_41_N_2_O_6_, 525.2965).

Capsimycin B (**3**): yellow-beige powder; 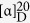 + 129.8 (c 0.08, MeOH); UV (MeOH) λ_max_ (log ε) 323 (3.88); CD (c 1.07 mM, MeOH) λ_max_ (Δε) 214 (+9.29), 242 (−11.77), 325 (+5.55); IR (KBr) ν_max_ 3444, 2928, 1643, 1434, 1384, 1114 cm^−1^; ^1^H and ^13^C NMR data, Tables 1 and 2; HRESIMS m/z 495.2854 [M + H]^+^ (calcd for C_29_H_39_N_2_O_5_, 495.2859).

Capsimycin C (**4**): pale yellow powder; 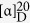 + 58.4 (c 0.13, MeOH); UV (MeOH) λ_max_ (log ε) 322 (3.54); CD (c 1.31 mM, MeOH) λ_max_ (Δε) 212 (+2.71), 246 (−5.21), 326 (+2.63); IR (KBr) ν_max_ 3448, 2924, 1639, 1384, 1113 cm^−1^; ^1^H and ^13^C NMR data, Tables 1 and 2; HRESIMS m/z 513.2958 [M + H]^+^ (calcd for C_29_H_41_N_2_O_6_, 513.2965).

Capsimycin D (**5**): pale yellow powder; 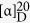 + 76.7 (c 0.20, MeOH); UV (MeOH) λ_max_ (log ε) 323 (3.72); CD (c 1.01 mM, MeOH) λ_max_ (Δε) 215 (+3.62), 239 (−6.14), 325 (+3.65); IR (KBr) ν_max_ 3447, 2925, 1642, 1384, 1113 cm^−1^; ^1^H and ^13^C NMR data, Tables 1 and 2; HRESIMS m/z 531.2623 [M + H]^+^ (calcd for C_29_H_40_N_2_O_5_Cl, 531.2626).

Capsimycin E (**6**): pale yellow powder; 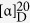 + 131.8 (c 0.25, MeOH); UV (MeOH) λ_max_ (log ε) 324 (3.88); CD (c 1.69 mM, MeOH) λ_max_ (Δε) 213 (+8.37), 238 (−12.64), 329 (+6.17); IR (KBr) ν_max_ 3448, 2926, 1640, 1433, 1384, 1107 cm^−1^; ^1^H and ^13^C NMR data, Tables 1 and 2; HRESIMS m/z 557.3235 [M + H]^+^ (calcd for C_31_H_45_N_2_O_7_, 557.3227).

Capsimycin F (**7**): pale yellow powder; 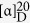 + 107.1 (c 0.13, MeOH); UV (MeOH) λ_max_ (log ε) 325 (3.94); CD (c 1.77 mM, MeOH) λ_max_ (Δε) 214 (+9.15), 238 (−12.45), 326 (+6.56); IR (KBr) ν_max_ 3448, 2924, 1639, 1432, 1383, 1108 cm^−1^; ^1^H and ^13^C NMR data, Tables 1 and 2; HRESIMS m/z 527.3104 [M + H]^+^ (calcd for C_30_H_43_N_2_O_6_, 527.3121).

Capsimycin G (**3**′): pale yellow powder; 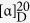 + 94.6 (c 0.11, MeOH); UV (MeOH) λ_max_ (log ε) 322 (3.70); CD (c 1.18 mM, MeOH) λ_max_ (Δε) 211 (+5.64), 248 (−9.90), 325 (+3.86); IR (KBr) ν_max_ 3432, 2931, 1655, 1600, 1465, 1384, 1233, 1134 cm^−1^; ^1^H and ^13^C NMR data, Tables 1 and 2; HRESIMS m/z 511.2803 [M + H]^+^ (calcd for C_29_H_39_N_2_O_6_, 511.2808).

### IC_50_ analysis and LDH testing

The cells were seeded into 96-well plates at 5 × 10^3^ cells per well. After overnight incubations, Dulbecco’s Modified Eagle Medium (Thermo Fisher Scientific, Inc.) was replaced with fresh medium, with or without different concentrations (0–10 μM) of compounds **1**–**7**. Cell viability was measured 24 h later using Cell Counting Kit-8 (CCK-8, Dojindo Molecular Technologies, Japan) following the manufacturer’s protocols. The half maximal inhibitory concentration (IC_50_) was defined as the concentration that yielded a 50% reduction in cell growth compared to control cells. Data are presented as the mean ± standard deviation (SD) of three independent experiments, each performed in triplicate.

## Additional Information

**How to cite this article**: Yu, H.-L. *et al*. Structural diversity of anti-pancreatic cancer capsimycins identified in mangrove-derived *Streptomyces xiamenensis* 318 and post-modification *via* a novel cytochrome P450 monooxygenase. *Sci. Rep.* **7**, 40689; doi: 10.1038/srep40689 (2017).

**Publisher's note:** Springer Nature remains neutral with regard to jurisdictional claims in published maps and institutional affiliations.

## Supplementary Material

Supporting Information

## Figures and Tables

**Table 1:** ^1^H NMR Spectroscopic Data for Compounds **3′-7** (*δ* in ppm, *J* in Hz, 600 MHz).

Position	*δ*_H_, multi (*J* in H_Z_)				
	3′^###^	4^##^	5^##^	6^#^	7^#^
2	3.78, br d (3.8)	3.79, dd (5.5, 2.1)	3.88, dd (5.5, 2.1)	3.85, br d (4.0)	3.85, br d (3.87)
3	1.85, m	1.71, m	1.82, m	1.84, m	1.84, m
	2.00, m	1.96, m	2.05, m	1.99, m	1.99, m
4	1.26, m	1.06, m	1.18, m	1.18, m	1.19, m
	1.52, m	1.51, m	1.62, m	1.54, m	1.54, m
5	2.64, m	2.54, br t (11.5)	2.65, br t (11.2)	2.66, br t (11.0)	2.66, br t (10.9)
	3.46, br d (12.0)	3.50, ddd (11.5, 4.6, 2.9)	3.55, ddd (11.2, 4.9, 3.0)	3.40, m	3.40, m
8	5.91, d (11.9)	5.75, dd (11.6, 1.9)	5.84, dd (11.5, 1.3)	5.84, d (11.3)	5.84, d (10.8)
9	6.05, ddd (11.9, 10.1, 2.8)	5.99, dd (11.6, 3.1)	6.06, ddd (11.5, 11.5, 3.4)	6.06, td (11.3, 3.8)	6.06, td (10.8, 3.8)
10	2.42, m	2.40, ddd (17.5, 3.0, 3.0)	2.53, dd (17.3, 3.0)	2.45, d (15.7)	2.45, br d (15.3)
	3.63, m	3.29, m	3.38, m	3.49, m	3.46, m
11	1.61, m	1.97, m	2.14, m	2.01, m	2.00, m
12	2.28, m	1.91, dd (10.6, 4.0)	2.07, m*	2.02, m	2.01, m
13	2.89, d (3.8)	3.72, br t (3.8)	4.13, br t (2.6)	4.08, br	4.08, br t (2.2)
14	3.26, br s	3.74, br t (3.8)	4.23, br t (2.9)	3.46, t (2.7)	3.38, m
15	0.93, m	1.38, dd (11.0, 3.6)	1.77, m	1.69, dd (11.2, 2.6)	1.46, dd (11.5, 2.7)
16	1.88, m	1.68, m	1.76, d (3.3)	2.06, m	1.73, m
17	2.39, m	2.10, m	2.21, m	2.18, m	2.16, m
18	0.65, td (11.9, 8.1)	0.61, m	0.75, m	0.67, m	0.66, m
	2.04, m	2.05, dd (12.3, 7.5)	2.19, d (7.6)	2.03, m	2.14, m
19	1.12, m	1.51, m	1.61, m	1.56, m	1.53, m
20	1.68, m	1.90, m	2.07, m*	2.00, m	1.98, m
21	1.10, m	1.20, m	1.29, m	1.27, m	1.24, m
	2.02, m	1.99, m	2.13, dd (7.6, 4.8)	2.08, m	2.08, m
22	2.35, m	2.33, m	2.41, m	2.37, m	2.37, m
23	6.68, dd (15.0, 9.8)	6.75, dd (15.4, 10.3)	6.83, dd (15.4, 10.3)	6.75, dd (15.8, 10.5)	6.74, dd (15.3, 10.4)
24	7.28, m	7.03, d (15.4)	7.13, d (15.4)	7.13, d (15.8)	7.13, d (15.3)
29	1.08, d (7.7)	0.81, d (7.0)	0.90, d (6.8)	1.00, d (7.1)	0.89, d (3.5)
30	3.87, m	1.27, m	1.35, m	3.39, m	1.37, m
31	1.36, d (6.3)	0.86, t (7.4)	0.94, t (7.4)	1.20, d (6.1)	0.95, t (7.6)
32				3.29, s	
33				3.42, s	3.42, s

*Inseperatable; ^#^measured in CD_3_OD; ^##^measured in 90% CDCl_3_/CD_3_OD. ^###^measured in 90% CD_3_OD/C_6_D_6_.

**Table 2:** ^13^C NMR Spectroscopic Data for **3′–7** (*δ* in ppm, 150 MHz).

Position	*δ*_C_, type				
	3′^###^	4^##^	5^##^	6^#^	7^#^
1	198.0, C	197.5, C	197.1, C	198.2, C	198.2, C
2	62.5, CH^∗∗^	61.8, CH	61.6, CH	62.9, CH	62.9, CH
3	27.9, CH_2_	27.5, CH_2_	27.5, CH_2_	28.3, CH_2_	28.3, CH_2_
4	21.7, CH_2_	21.3, CH_2_	21.1, CH_2_	22.0, CH_2_	22.0, CH_2_
5	39.7, CH_2_	39.2, CH_2_	39.0, CH_2_	40.0, CH_2_	40.0, CH_2_
7	168.6, C	167.7, C	167.2, C	168.8, C	168.9, C
8	124.9, CH	123.6, CH	123.7, CH	124.7, CH	124.7, CH
9	141.4, CH	142.3, CH	141.6, CH	142.6, CH	142.7, CH
10	26.8, CH_2_	26.9, CH_2_	26.4, CH_2_	27.6, CH_2_	27.6, CH_2_
11	47.1, CH	46.0, CH	45.6, CH	45.9, CH	45.7, CH
12	41.5, CH	46.6, CH	47.3, CH	49.6, CH	49.6, CH
13	55.1, CH	74.6, CH	73.7, CH	68.7, CH	68.7, CH
14	59.4, CH	72.1, CH	64.7, CH	83.0, CH	82.5, CH
15	48.3, CH	48.0, CH	47.0, CH	44.7, CH	47.6, CH
16	52.8, CH	42.5, CH	44.7, CH	45.9, CH	43.8, CH
17	34.8, CH	33.3, CH	32.6, CH	35.0, CH	33.8, CH
18	40.0, CH_2_	39.5, CH_2_	38.6, CH_2_	40.9, CH_2_	40.2, CH_2_
19	48.6, CH	41.0, CH	41.1, CH	42.7, CH	42.9, CH
20	42.5, CH	43.5, CH	42.6, CH	42.6, CH	43.0, CH
21	38.0, CH_2_	36.1, CH_2_	35.6, CH_2_	36.3, CH_2_	36.3, CH_2_
22	50.0, CH	49.9, CH	49.6, CH	51.2, CH	51.2, CH
23	149.3, CH	153.4, CH	153.0, CH	153.0, CH	153.2, CH
24	124.7, CH	122.3, CH	122.2, CH	123.7, CH	123.3, CH
25	176.3, C^∗^	173.7, C	173.6, C	174.1, C^∗^	173.7, C^∗^
26	102.4, C^∗^^∗^	101.0, C	100.8, C	102.2, C	102.2, C
27	177.5, C	175.8, C	175.6, C	176.9, C	177.1, C
29	18.3, CH_3_	17.6, CH_3_	17.7, CH_3_	18.4, CH_3_	18.0, CH_3_
30	68.9, CH	21.9, CH_2_	21.2, CH_2_	79.1, CH	22.6, CH_2_
31	23.5, CH_3_	13.0, CH_3_	12.8, CH_3_	18.0, CH_3_	13.6, CH_3_
32				55.8, CH_3_	
33				58.6, CH_3_	59.0, CH_3_

^∗^Observed in HMBC spectra; ^∗∗^low intensity. ^#^Measured in CD_3_OD; ^##^Measured in 90% CDCl_3_/CD_3_OD. ^###^Measured in 90% CD_3_OD/C_6_D_6_.
