# Supplementary material for: Structural diversity of anti-pancreatic cancer capsimycins identified in mangrove-derived Streptomyces xiamenensis 318 and post-modification via a novel cytochrome P450 monooxygenase
Source: Sci Rep. 2017 Jan 18;7:40689. doi: 10.1038/srep40689 (PMC5241641; doi:10.1038/srep40689)
Supplement: Supporting Information [file srep40689-s1.pdf]

Structural diversity of anti-pancreatic cancer capsimycins identified in mangrove-derived *Streptomyces xiamenensis* 318 and post-modification *via* a novel cytochrome P450 monooxygenase

He-Lin YU[1], Shu-Heng Jiang[2], Xu-Liang BU [3], Jia-Hua WANG [3], Jing-Yi Weng[1], Xiao-Mei Yang[2], Kun-Yan He[1], Zhi-Gang Zhang[2], Ping AO[1], Jun Xu [3]* and Min-Juan XU[1]*

[1] Ministry of Education Key Laboratory of Systems Biomedicine, Shanghai Centre for Systems Biomedicine, Shanghai Jiao Tong University, Shanghai 200240, China

[2] State Key Laboratory of Oncogenes and Related Genes, Shanghai Cancer Institute, Ren Ji Hospital, Shanghai Jiao Tong University School of Medicine, Shanghai 200240, China

[3] State Key Laboratory of Microbial Metabolism and School of Life Sciences and Biotechnology, Institute of Oceanology, Shanghai Jiao Tong University, Shanghai 200240, China

*Correspondence and requests for materials should be addressed to J. X. (xujunn@sjtu.edu.cn), M. X. (minjuanxu@sjtu.edu.cn)

Subject areas: *Streptomyces xiamenensis*, capsimycin, P450 monooxygenase, anti-pancreatic cancer

Supplementary materials

Figure S1. Coomassie blue stained SDS-PAGE gel analysis of IkaD. 8

Figure S2. Alignment of IkaD to some macrolide P450 monooxygenases. 8

Figure S3. Maximum-likelihood phylogenetic tree of the novel cytochrome P450 monooxgenase IkaD (full dendrogram). 9

Table S1. Summary of the P450 monooxygenases (Sxim_40690) BlastP analysis. 11

Figure S4. HPLC (A) and UPLC (B) profiles of ikarugamycin (1) conversions into capsimycin (3) and 3' catalyzed by IkaD *in vitro*. 12

Figure S5. HPLC analysis of the changes of capsimycin (2), capsimycin B (3), and ikarugamycin (1) in methanol solution (contained 1‰ TFA) at 45 ºC with different time. 13

Figure S6. Analysis of capsimycin (2) and capsimycin B (3) acidification. 14

Figure S7. Proposed three fragmentation patterns (A-C) of compound 1-7 in secondary mass spectrometry. 14

Table S2. Fragment ions of compounds 1-7 in the positive mode 15

Figure S8. ESI-HR-MS/MS of 1 18

Figure S9. ESI-HR-MS/MS of 2 18

Figure S10. ESI-HR-MS/MS of 3 19

Figure S11. ESI-HR-MS/MS of 4 19

Figure S12. ESI-HR-MS/MS of 5 20

Figure S13. ESI-HR-MS/MS of 6. 20

Figure S14. ESI-HR-MS/MS of 7. 21

Figure S15. ESI-HR-MS/MS of 3'. 21

Figure S16. UV spectra of compounds 1-7. 22

Table S3. ^{1}H (600 MHz) and ^{13}C (150 MHz) spectroscopic data of capsimycin (2). 23

Figure S17. HRESIMS of capsimycin (2) (Positve mode). 24

Figure S18. ^{1}H NMR (600 MHz, $CDCl_3$) spectrum of capsimycin (2). 24

Figure S19. ^{13}C NMR (600 MHz, $CDCl_3$) spectrum of capsimycin (2). 25

Figure S20. DEPT 135 (600 MHz, $CDCl_3$) spectrum of capsimycin (2). 25

Figure S21. HSQC (600 MHz, $CDCl_3$) of capsimycin (2). 26

Figure S22. HMBC (600 MHz, $CDCl_3$) of capsimycin (2). 26

Figure S23. COSY (600 MHz, $CDCl_3$) of capsimycin (2). 27

Figure S24-a. NOESY (600 MHz, $CDCl_3$) of capsimycin (2). 27

Figure S24-b. ROESY (600 MHz, $CDCl_3$) of capsimycin (2). 28

Figure S25. HRESIMS of 3 (Positve mode). 28

Table S4. ^{1}H (600 MHz) and ^{13}C (150 MHz) spectroscopic data of 3. 29

Figure S26. ^{1}H NMR (600 MHz, $CDCl_3$) spectrum of 3. 30

Figure S27. ^{13}C NMR (600 MHz, $CDCl_3$) spectrum of 3. 30

Figure S28. ^{13}C-DEPT 135 (600 MHz, $CDCl_3$) spectrum of 3. 31

Figure S29. HSQC (600 MHz, $CDCl_3$) of 3. 31

Figure S30. HMBC (600 MHz, $CDCl_3$) of 3. 32

Figure S31. COSY (600 MHz, $CDCl_3$) of 3. 32

Figure S32. NOESY (600 MHz, $CDCl_3$) of 3. 33

Figure S33. HRESIMS of 4 (Positve mode). 33

Table S5. ^1H (600 MHz) and ^{13}C (150 MHz) spectroscopic data of 4. 34

Figure S34. ^1H NMR (600 MHz, MeOD/$CDCl_3$) spectrum of 4. 35

Figure S35. ^{13}C NMR (600 MHz, MeOD/$CDCl_3$) spectrum of 4. 35

Figure S36. ^{13}C-DEPT NMR (600 MHz, MeOD/$CDCl_3$) spectrum of 4. 36

Figure S37. HSQC (600 MHz, MeOD/$CDCl_3$) of 4. 36

Figure S38. HMBC (600 MHz, MeOD/$CDCl_3$) of 4. 37

Figure S39. COSY (600 MHz, MeOD/$CDCl_3$) of 4. 37

Figure S40. TOCSY (600 MHz, MeOD/$CDCl_3$) of 4. 38

Figure S41-a. NOESY (600 MHz, MeOD/$CDCl_3$) of 4. 38

Figure S41-b. ROESY (600 MHz, MeOD/$CDCl_3$) of 4. 39

Figure S42-a. FT-MS of 5 (Positve mode). 40

Figure S42-b. HR-MS of 5 (Positve mode). 40

Table S6. ^1H (600 MHz) and ^{13}C (150 MHz) spectroscopic data of 5. 41

Figure S43. ^1H NMR (600 MHz, MeOD/$CDCl_3$) spectrum of 5. 42

Figure S44. ^{13}C NMR (600 MHz, MeOD/$CDCl_3$) spectrum of 5. 42

Figure S45. ^{13}C-DEPT NMR (600 MHz, MeOD/$CDCl_3$) spectrum of 5. 43

Figure S46. HSQC (600 MHz, MeOD/$CDCl_3$) of 5. 43

Figure S47. HMBC (600 MHz, MeOD/$CDCl_3$) of 5. 44

Figure S48. COSY (600 MHz, MeOD/$CDCl_3$) of 5. 44

Figure S49. TOCSY (600 MHz, MeOD/$CDCl_3$) of 5. 45

Figure S50-a. NOESY (600 MHz, MeOD/$CDCl_3$) of 5. 45

Figure S50-b. ROESY (600 MHz, MeOD/$CDCl_3$) of 5. 46

Figure S51. HRESIMS of 6 (Positve mode). 46

Table S7. ^{1}H (600 MHz) and ^{13}C (150 MHz) spectroscopic data of 6. 47

Figure S52. ^{1}H NMR (600 MHz, MeOD) spectrum of 6. 48

Figure S53. ^{13}C NMR (600 MHz, MeOD) spectrum of 6. 48

Figure S54. ^{13}C-DEPT NMR (600 MHz, MeOD) spectrum of 6. 49

Figure S55. HSQC (600 MHz, MeOD) of 6. 49

Figure S56. HMBC (600 MHz, MeOD) of 6. 50

Figure S57. COSY (600 MHz, MeOD) of 6. 50

Figure S58-a. NOESY (600 MHz, MeOD) of 6. 51

Figure S58-b. ROESY (600 MHz, MeOD) of 6. 51

Table S8. ^{1}H (600 MHz) and ^{13}C (150 MHz) spectroscopic data of 7. 52

Figure S59. HRESIMS of 7 (Positve mode). 53

Figure S60. ^{1}H NMR (600 MHz, MeOD) spectrum of 7. 53

Figure S61. ^{13}C NMR (600 MHz, MeOD) spectrum of 7. 54

Figure S62. ^{13}C-DEPT NMR (600 MHz, MeOD) spectrum of 7. 54

Figure S63. HSQC (600 MHz, MeOD) of 7. 55

Figure S64. HMBC (600 MHz, MeOD) of 7. 55

Figure S65. COSY (600 MHz, MeOD) of 7. 56

Figure S66-a. NOESY (600 MHz, MeOD) of 7. 56

Figure S66-b. ROESY (600 MHz, MeOD) of 7. 57

Table S9. ^{1}H (600 MHz) and ^{13}C (150 MHz) spectroscopic data of 3'............ 58

Figure S67. HRESIMS of 3' (Positve mode). 59

Figure S68. ^{1}H NMR (600 MHz, 90% MeOD/C_6D_6) spectrum of 3'. 59

Figure S69. ^{13}C NMR (600 MHz, 90% MeOD/C_6D_6) spectrum of 3'. 60

Figure S70. ^{13}C-DEPT NMR (600 MHz, 90% MeOD/C_6D_6) spectrum of 3'... 60

Figure S71. HSQC (600 MHz, 90% MeOD/C_6D_6) of 3'. 61

Figure S72. HMBC (600 MHz, 90% MeOD/C_6D_6) of 3'. 61

Figure S73. COSY (600 MHz, 90% MeOD/C_6D_6) of 3'. 62

Figure S74. NOESY (600 MHz, 90% MeOD/C_6D_6) of 3'. 62

Figure S75. Kinetic analysis with Surface Plasmon Resonance (SPR) for A) ikarugamycin (1) and B) capsimycin B (3) binding to the IkaD. 63

Table S10. Binding affinities and kinetic parameters for the interactions between ikarugamycin (1) and capsimycin B (3) with IkaD as measured with SPR. 63

Figure S76. The reactions of capsimycin B (3) with NaX (X = Cl, Br, I). Targeted products were detected under the following conditions. A) Reaction of compound 3 in MeOH/H_2O; UPLC-total ion chromatography MS profiles of the products produced under the condition of B) 1 h after the mixing with TFA at room temperature; C) 1 h after the mixing with TFA at 45 ºC; D) MS spectra

of (a) capsimycin B + HCl, (b) capsimycin B + HBr, and (c) capsimycin B + HI. 65

Figure S77. The reactions of capsimycin B (3) with NaX (X = Cl, Br, I). No products were detected under the following conditions. A) Reaction of compound 3 in MeOH/H_2O; UPLC-total ion chromatography MS profiles of the products produced under the condition of B) immediately after the mixing at room temperature; C) 1 h after the mixing at room temperature; D) 1 h after the mixing at 45 ºC.. 66

Figure S78. The reactions of capsimycin B (3) with H_2O. Targeted product (4) was detected under the following conditions. A) Reaction of compound 3 in MeOH/H_2O can be accelerated by the TFA; B) UPLC-total ion chromatography MS profiles of the products produced under the condition of a) immediately after the mixing at room temperature; b) 1 h after the mixing at 45 ºC; c) 1 h after the mixing with TFA at room temperature; (d) 1 h after the mixing with TFA at 45 ºC. 67

Figure S79. Gel filtration of purified IkaD indicated with the blue line........... 68

Figure S80. IkaD identification by nano LC-MS/MS and database search...... 69

Protein identification by nano LC-MS/MS and database search...................... 70

Figure S81. Expended NOESY spectra (600 MHz, MeOD/$CDCl_3$) of 4 (A-C) and expended ROESY spectra (600 MHz, MeOH/$CDCl_3$) of 5 (D-E)............. 71

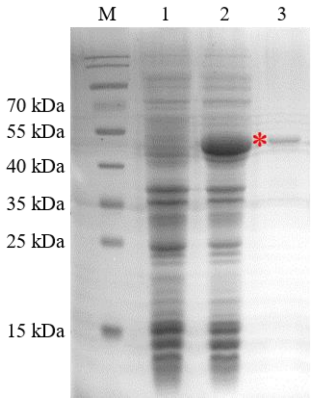

Figure S1. Coomassie blue stained SDS-PAGE gel analysis of IkaD.

Lanes: M, protein marker; 1, control, cell free supernatant of crude *E.* coli Rosetta (DE3) without IPTG induced; 2, expressing IkaD by IPTG induced; 3, purified C-terminal 6 × His tagged IkaD.

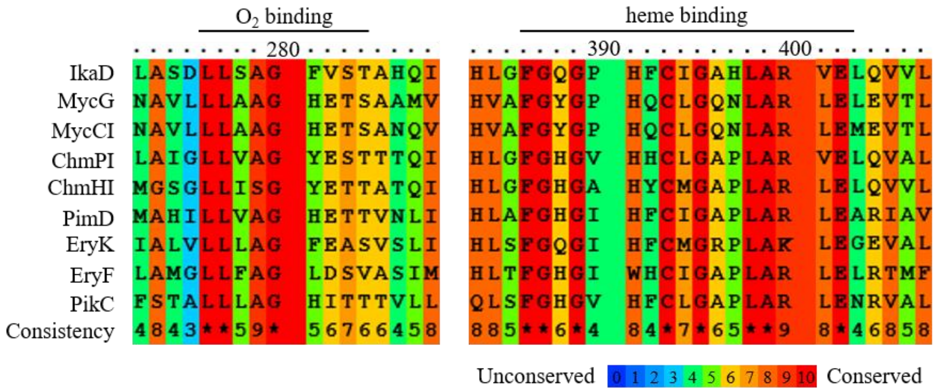

Figure S2. Alignment of IkaD to some macrolide P450 monooxygenases.

The O_2 binding region and the heme binding pocket were showed as the solid line on the left and right, respectively. The scoring scheme works from 0 for the least conserved alignment position, up to 10 for the most conserved alignment position. MycG and MycCI (accession numbers Q59523 and Q83WF5), ChmPI and ChmHI (accession numbers AAS79447 and AAS79453), PimD (accession number CAC20932), EryK and EryF (accession numbers P48635 and AAA26496), and PikC (accession number O87605). The results were analysed from website of Centre for Integrative Bioinformatics VU (http://www.ibi.vu.nl/programs/#msa).

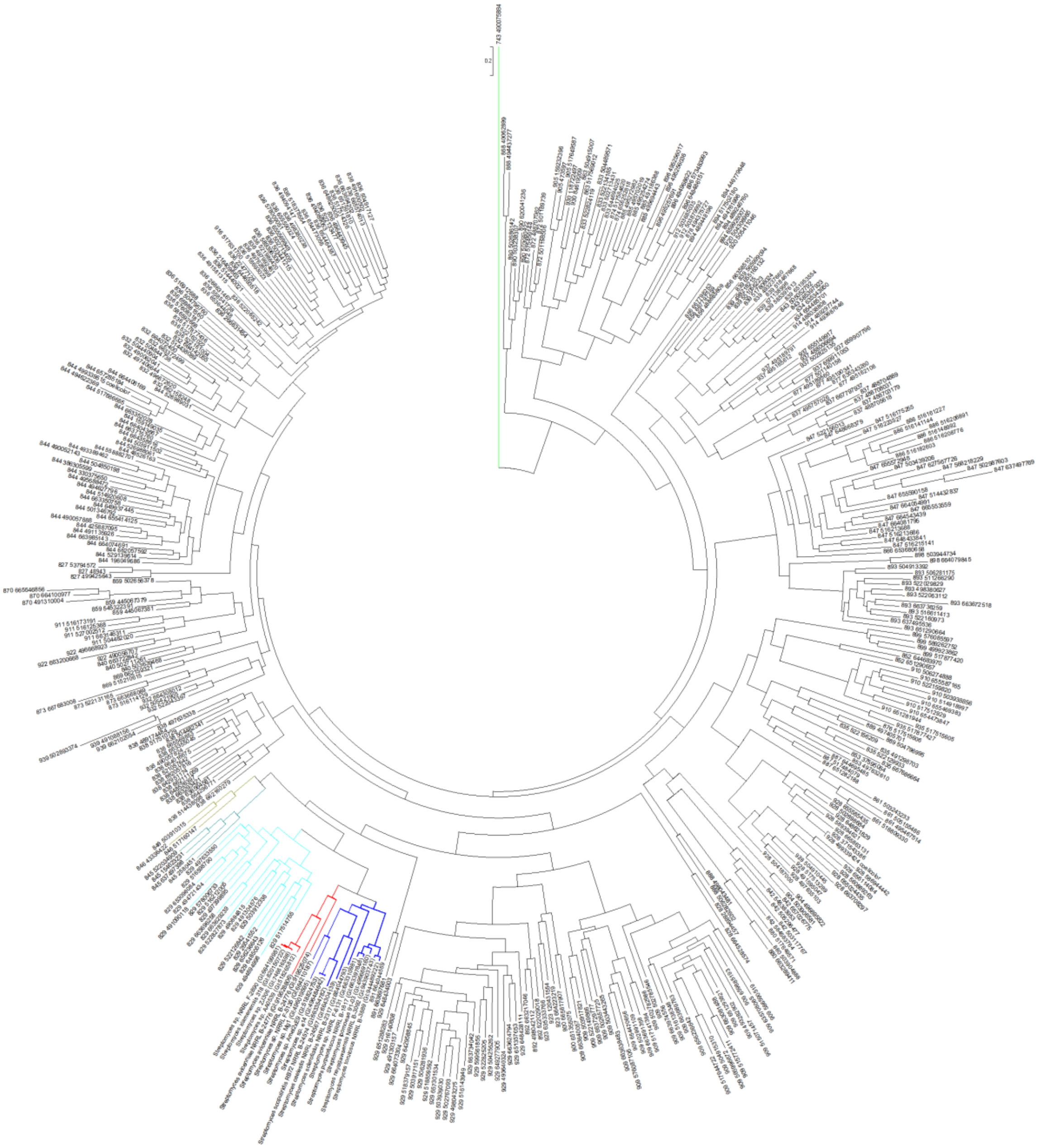

Figure S3. Maximum-likelihood phylogenetic tree of the novel cytochrome P450 monooxgenase IkaD (full dendrogram).

Maximum-likelihood phylogenetic tree of 430 representative amino acid sequences in the CYP107-clan and 6 CYP107-clan-like amino acid sequences, including the cytochrome P450 monooxgenase IkaD (SXIM_40690, GI:820150722). The tree was rooted using a

CYP102-clan of P450 superfamily member (GI:490075884 from *S. coelicolor*). The part of the dendrogram shown in Figure 5 was labeled in color. Bar, 0.02 substitutions per nucleotide position. Number for each branch is consisted of three letters that stand for the homologues family id according to CYPED, and follow by a string of NCBI gi number after the blank space.

Table S1. Summary of the P450 monooxygenases (Sxim_40690) BlastP analysis.

Query protein	Size*	Locus	Name	Identity/Coverage				
	1			2	3	4	5	6
WP_046725809.1	3125	SXIM_40840	IkaA	99/100	99/99	87/99	70/99	67/99
WP_053116264.1	611	SXIM_40830	IkaB	100/100	99/97	92/93	75/97	68/90
WP_030733536.1	349	SXIM_40820	IkaC	99/100	\	89/100	66/99	64/100
WP_046724814.1	409	SXIM_40690	IkaD	99/100	99/100	90/100	59/97	\

1, *Streptomyces* xiamenensis 318; 2, *Streptomyces* sp. ZJ306; 3, *Streptomyces* sp. NRRL F-2890; 4, *Streptomyces* sp. AA0539; 5, *Streptomyces* avicenniae; 6, *Streptomyces* grisesus. Size*, amino acid numbers.

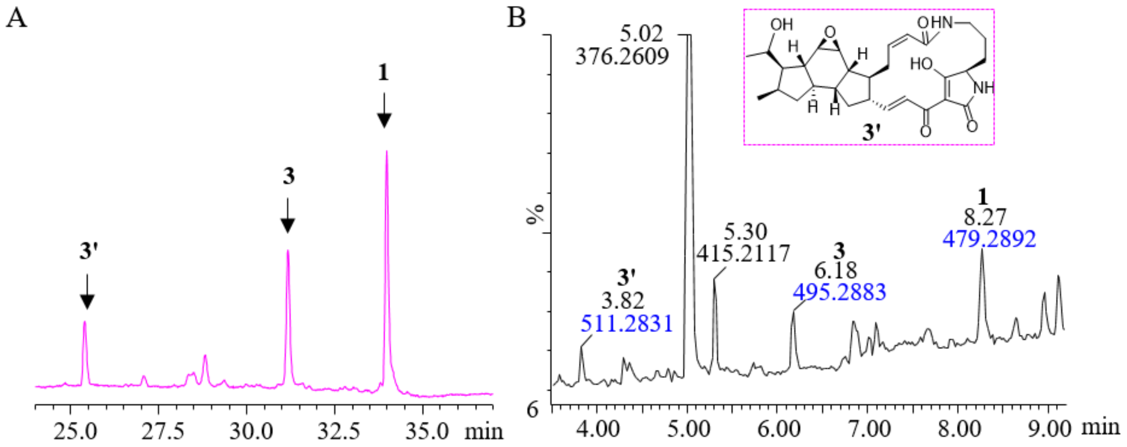

Figure S4. HPLC (A) and UPLC (B) profiles of ikarugamycin (**1**) conversions into capsimycin (**3**) and **3'** catalyzed by IkaD *in vitro*.

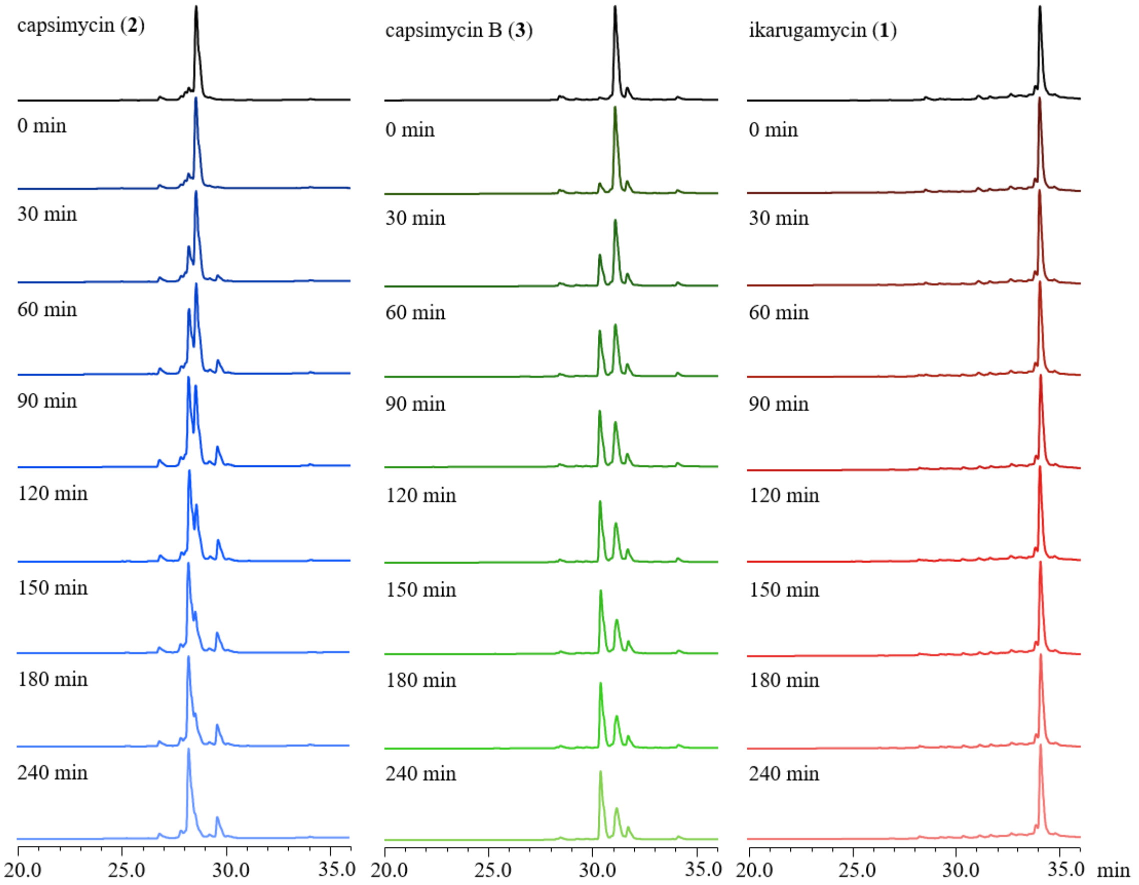

Figure S5. HPLC analysis of the changes of capsimycin (**2**), capsimycin B (**3**), and ikarugamycin (**1**) in methanol solution (contained 1‰ TFA) at 45 ºC with different time.

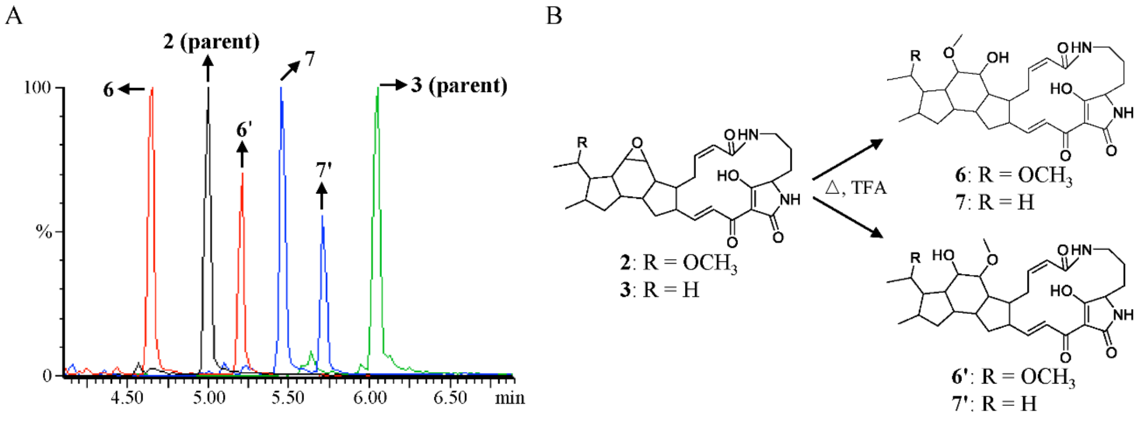

Figure S6. Analysis of capsimycin (**2**) and capsimycin B (**3**) acidification.

A) Extracted ion chromatography of compounds related to acidized **2** and **3** by UPLC-ESI-MS. B) proposed mechanisms for acidification of **2** and **3**.

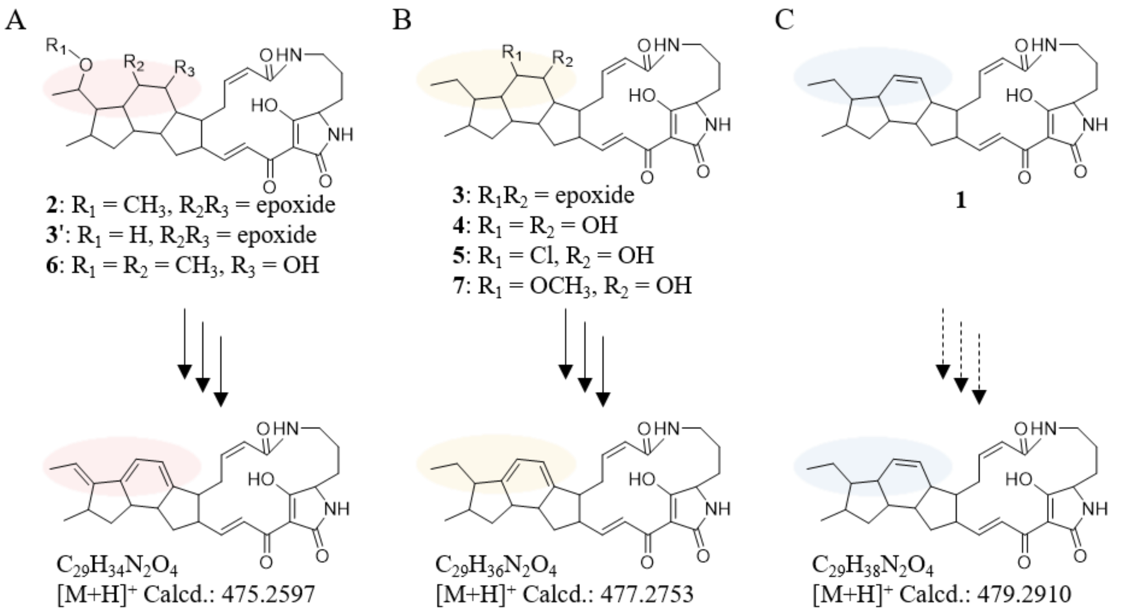

Figure S7. Proposed three fragmentation patterns (A-C) of compound **1-7** in secondary mass spectrometry.

Table S2. Fragment ions of compounds **1-7** in the positive mode.

	m/z Found	m/z Calculated	%Base	Formula	mDa	ppm
ika (**1**)	479.2908	479.2910	100	$C_{29}H_{39}N_2O_4$	-0.2	-0.4
	461.2809	461.2804	52	$C_{29}H_{37}N_2O_3$	0.5	1.1
	443.2706	443.2699	52	$C_{29}H_{35}N_2O_2$	0.7	1.6
	433.2835	433.2855	12	$C_{28}H_{37}N_2O_2$	-2.0	-4.6
	425.2594	425.2593	16	$C_{29}H_{33}N_2O$	0.1	0.2
	323.2031	323.2011	26	$C_{22}H_{27}O_2$	2.0	6.2
	305.1914	305.1905	40	$C_{22}H_{25}O$	0.9	2.9
	295.2082	295.2062	27	$C_{21}H_{27}O$	2.0	6.8
	281.1920	281.1905	84	$C_{20}H_{25}O$	1.5	5.3
	263.1814	263.1800	69	$C_{20}H_{23}$	1.4	5.3
	253.1966	253.1956	99	$C_{19}H_{25}$	1.0	3.9
	211.1488	211.1487	40	$C_{16}H_{19}$	0.1	0.5
	193.0997	193.0977	50	$C_{10}H_{13}N_2O_2$	2.0	10.4
	181.0990	181.0977	55	$C_9H_{13}N_2O_2$	1.3	7.2
	165.0679	165.0664	79	$C_8H_9N_2O_2$	1.5	9.1
	139.0882	139.0871	97	$C_7H_{11}N_2O$	1.1	7.9
	109.1028	109.1017	39	C_8H_{13}	1.1	10.1
cap (**2**)	525.2966	525.2965	39	$C_{30}H_{40}N_2O_6$	0.1	0.2
	507.2869	507.2859	15	$C_{30}H_{39}N_2O_5$	1.0	2.0
	493.2694	493.2702	100	$C_{29}H_{37}N_2O_5$	-0.8	-1.6
	475.2598	475.2597	98	$C_{29}H_{35}N_2O_4$	0.1	0.2
	457.2501	457.2491	42	$C_{29}H_{33}N_2O_3$	1.0	2.2
	449.2454	449.2440	22	$C_{27}H_{33}N_2O_4$	1.4	3.1
	439.2401	439.2386	15	$C_{29}H_{31}N_2O_2$	1.5	3.4
	429.2520	429.2542	11	$C_{28}H_{33}N_2O_2$	-2.2	-5.1
	319.1720	319.1698	14	$C_{22}H_{23}O_2$	2.2	6.9
	291.1761	291.1749	14	$C_{21}H_{23}O$	1.4	4.1
	277.1612	277.1592	45	$C_{20}H_{21}O$	2.0	7.2
	249.1616	249.1643	16	$C_{19}H_{21}$	-2.7	-10.8
	209.1350	209.1330	22	$C_{16}H_{17}$	2.0	9.6
	193.0999	193.0977	22	$C_{10}H_{13}N_2O_2$	2.2	11.4
	181.1000	181.0977	32	$C_9H_{13}N_2O_2$	2.3	12.7
	165.0684	165.0664	47	$C_8H_9N_2O_2$	2.0	12.1
	139.0892	139.0871	50	$C_7H_{11}N_2O$	2.1	15.1
	122.0628	122.0606	6	C_7H_8NO	2.2	18.0
cap B (**3**)	495.2853	495.2859	86	$C_{29}H_{39}N_2O_5$	-0.6	-1.2
	477.2761	477.2753	100	$C_{29}H_{37}N_2O_4$	0.8	1.7
	459.2661	459.2648	54	$C_{29}H_{35}N_2O_3$	1.3	2.8
	441.2549	441.2542	20	$C_{29}H_{33}N_2O_2$	0.7	1.6
	431.2694	431.2699	14	$C_{28}H_{35}N_2O_2$	-0.5	-1.2
	321.1855	321.1855	15	$C_{22}H_{25}O_2$	0.0	0.0
	293.1916	293.1905	14	$C_{21}H_{25}O$	1.1	3.8
	279.1766	279.1749	40	$C_{20}H_{23}O$	1.7	6.1
	251.1799	251.1800	18	$C_{19}H_{23}$	-0.1	-0.4
	211.1487	211.1487	10	$C_{16}H_{19}$	0.0	0.0
	193.0991	193.0977	20	$C_{10}H_{13}N_2O_2$	1.4	7.3

	181.0993	181.0977	34	$C_9H_{13}N_2O_2$	1.6	8.8
	165.0679	165.0664	55	$C_8H_9N_2O_2$	1.5	9.1
	139.0885	139.0871	55	$C_7H_{11}N_2O$	1.4	10.1
cap C (**4**)	513.2963	513.2965	17	$C_{29}H_{41}N_2O_6$	-0.2	0.4
	495.2858	495.2859	42	$C_{29}H_{39}N_2O_5$	-0.1	-0.2
	477.2717	477.2753	100	$C_{29}H_{37}N_2O_4$	-3.6	-7.5
	459.2641	459.2648	52	$C_{29}H_{35}N_2O_3$	-0.7	-1.5
	441.2534	441.2542	29	$C_{29}H_{33}N_2O_2$	-0.8	-1.8
	424.2310	424.2277	18	$C_{29}H_{30}NO_2$	3.3	7.6
	321.1847	321.1855	22	$C_{22}H_{25}O_2$	-0.8	-2.5
	303.1726	303.1749	20	$C_{22}H_{23}O$	-2.3	-7.6
	293.1906	293.1905	15	$C_{21}H_{25}O$	0.1	0.3
	279.1743	279.1749	82	$C_{20}H_{23}O$	-0.6	-2.1
	251.1777	251.1800	28	$C_{19}H_{23}$	-2.3	-9.2
	211.1485	211.1487	31	$C_{16}H_{19}$	-0.2	-0.9
	193.0987	193.0977	24	$C_{10}H_{13}N_2O_2$	1.0	5.2
	181.0986	181.0977	33	$C_9H_{13}N_2O_2$	0.9	5.0
	165.0672	165.0664	43	$C_8H_9N_2O_2$	0.8	4.8
	139.0875	139.0871	50	$C_7H_{11}N_2O$	0.4	2.9
cap D (**5**)	531.2604	531.2626	51	$C_{29}H_{40}N_2O_5Cl$	-2.2	-4.1
	513.2505	513.2520	38	$C_{29}H_{38}N_2O_4Cl$	-1.5	-2.9
	495.2504	495.2495	15	$C_{28}H_{35}N_2O_6$	0.9	1.8
	477.2718	477.2753	64	$C_{29}H_{37}N_2O_4$	-3.5	-7.3
	459.2637	459.2648	41	$C_{29}H_{35}N_2O_3$	-1.1	-2.4
	441.2538	441.2542	31	$C_{29}H_{33}N_2O_2$	-0.4	-0.9
	424.2289	424.2277	21	$C_{29}H_{30}NO_2$	1.2	2.8
	321.1854	321.1855	22	$C_{22}H_{25}O_2$	-0.1	-0.3
	303.1750	303.1749	21	$C_{22}H_{23}O$	0.1	0.3
	293.1900	293.1905	21	$C_{21}H_{25}O$	-0.5	-1.7
	279.1743	279.1749	100	$C_{20}H_{23}O$	-0.6	-2.1
	251.1791	251.1800	41	$C_{19}H_{23}$	-0.9	-3.6
	211.1483	211.1487	34	$C_{16}H_{19}$	-0.4	-1.9
	193.0986	193.0995	34	$C_{10}H_{13}N_2O_2$	0.9	4.7
	181.0982	181.0977	47	$C_9H_{13}N_2O_2$	0.5	2.8
	165.0674	165.0664	59	$C_8H_9N_2O_2$	1.0	6.1
	139.0878	139.0871	51	$C_7H_{11}N_2O$	0.7	5.0
	109.1021	109.1017	16	C_8H_{13}	0.4	3.7
cap E (**6**)	557.3220	557.3227	5	$C_{31}H_{44}N_2O_7$	-0.7	-1.2
	525.2969	525.2965	22	$C_{30}H_{40}N_2O_6$	0.4	0.7
	507.2898	507.2859	19	$C_{30}H_{39}N_2O_5$	3.9	7.7
	493.2712	493.2702	20	$C_{29}H_{37}N_2O_5$	1.2	2.0
	475.2589	475.2597	100	$C_{29}H_{35}N_2O_4$	-0.8	-1.7
	457.2512	457.2491	33	$C_{29}H_{33}N_2O_3$	2.1	4.6
	449.2455	449.2440	43	$C_{27}H_{33}N_2O_4$	1.5	3.3
	439.2412	439.2386	14	$C_{29}H_{31}N_2O_2$	2.6	5.9
	431.2369	431.2335	16	$C_{27}H_{31}N_2O_3$	3.4	7.9
	319.1714	319.1698	12	$C_{22}H_{23}O_2$	1.6	5.0
	277.1609	277.1592	49	$C_{20}H_{21}O$	1.7	6.1
	249.1599	249.1643	11	$C_{19}H_{21}$	-4.4	-17.6
	209.1353	209.1330	26	$C_{16}H_{17}$	2.3	11.0
	193.1010	193.0977	16	$C_{10}H_{13}N_2O_2$	3.3	17.1
	181.1006	181.0977	18	$C_9H_{13}N_2O_2$	2.9	16.0
	165.0687	165.0664	21	$C_8H_9N_2O_2$	2.3	13.9

	139.0894	139.0871	27	$C_7H_{11}N_2O$	2.3	16.5
cap F (**7**)	527.3118	527.3121	15	$C_{30}H_{42}N_2O_6$	-0.3	-0.5
	509.2995	509.3015	15	$C_{30}H_{40}N_2O_5$	-2.0	-3.9
	495.2838	495.2859	33	$C_{29}H_{39}N_2O_5$	-2.1	-4.2
	477.2728	477.2753	100	$C_{29}H_{36}N_2O_4$	-2.5	-5.2
	459.2649	459.2648	55	$C_{29}H_{35}N_2O_3$	0.1	0.2
	441.2510	441.2542	33	$C_{29}H_{33}N_2O_2$	-3.2	-7.3
	424.2281	424.2277	18	$C_{29}H_{30}NO_2$	0.4	0.9
	321.1837	321.1855	23	$C_{22}H_{25}O_2$	-1.8	-5.6
	303.1750	303.1749	23	$C_{22}H_{23}O$	0.1	0.3
	279.1742	279.1749	95	$C_{20}H_{23}O$	-0.7	-2.5
	251.1781	251.1800	26	$C_{19}H_{23}$	-1.9	-7.6
	211.1487	211.1487	36	$C_{16}H_{19}$	0.0	0.0
	193.0986	193.0977	25	$C_{10}H_{13}N_2O_2$	0.9	4.7
	181.0982	181.0977	28	$C_9H_{13}N_2O_2$	0.5	2.8
	165.0677	165.0664	34	$C_8H_9N_2O_2$	1.3	7.9
	139.0879	139.0871	46	$C_7H_{11}N_2O$	0.8	5.8
capG (**3'**)	511.2825	511.2808	14	C29H39N2O6	1.7	3.3
	493.2687	493.2702	100	C29H37N2O5	-1.5	-3.0
	475.2615	475.2597	68	C29H35N2O4	1.8	3.8
	457.2487	457.2491	18	C29H33N2O3	-0.4	-0.9
	319.1688	319.1698	14	C22H23O2	-1.0	-3.1
	277.1656	277.1592	50	C20H21O1	6.4	23.1
	193.0978	193.0977	32	C10H13N2O2	0.1	0.5
	181.0948	181.0977	27	C9H13N2O2	-2.9	-16.0

Figure S8. ESI-HR-MS/MS of **1**.

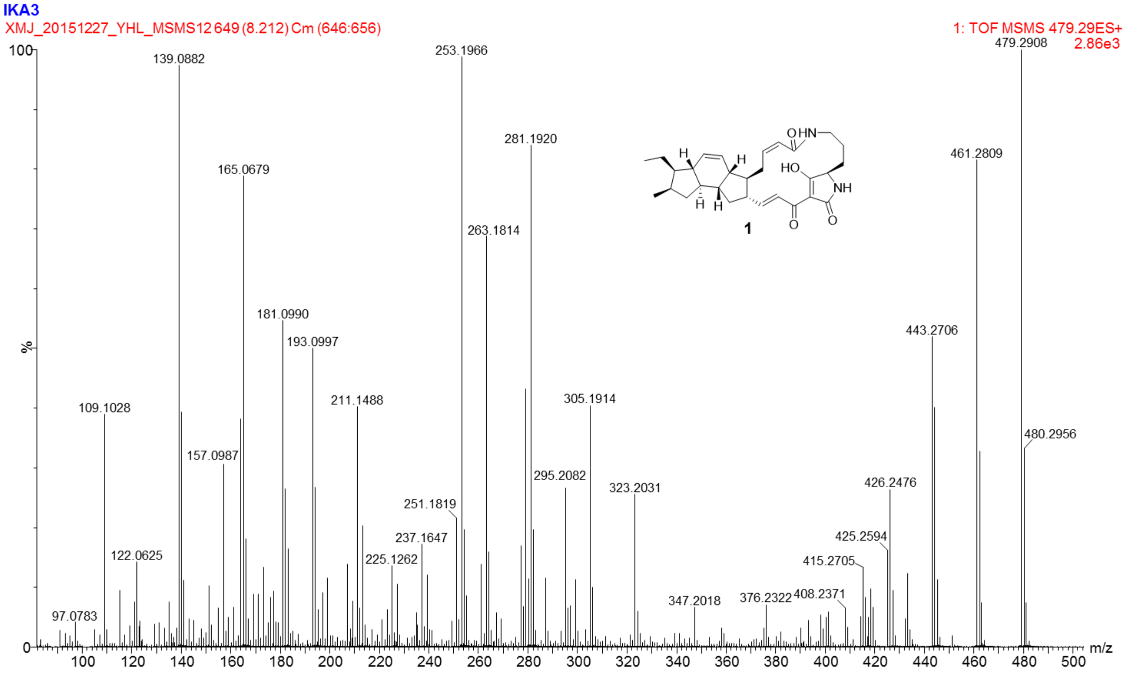

Figure S9. ESI-HR-MS/MS of **2**.

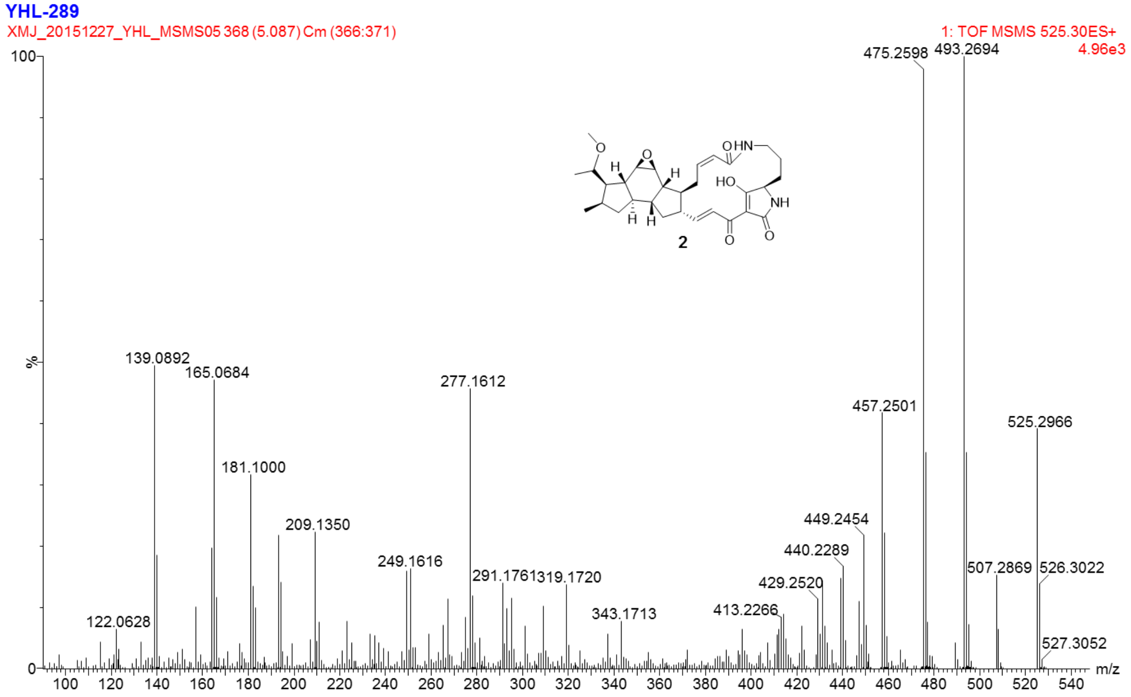

Figure S10. ESI-HR-MS/MS of **3**.

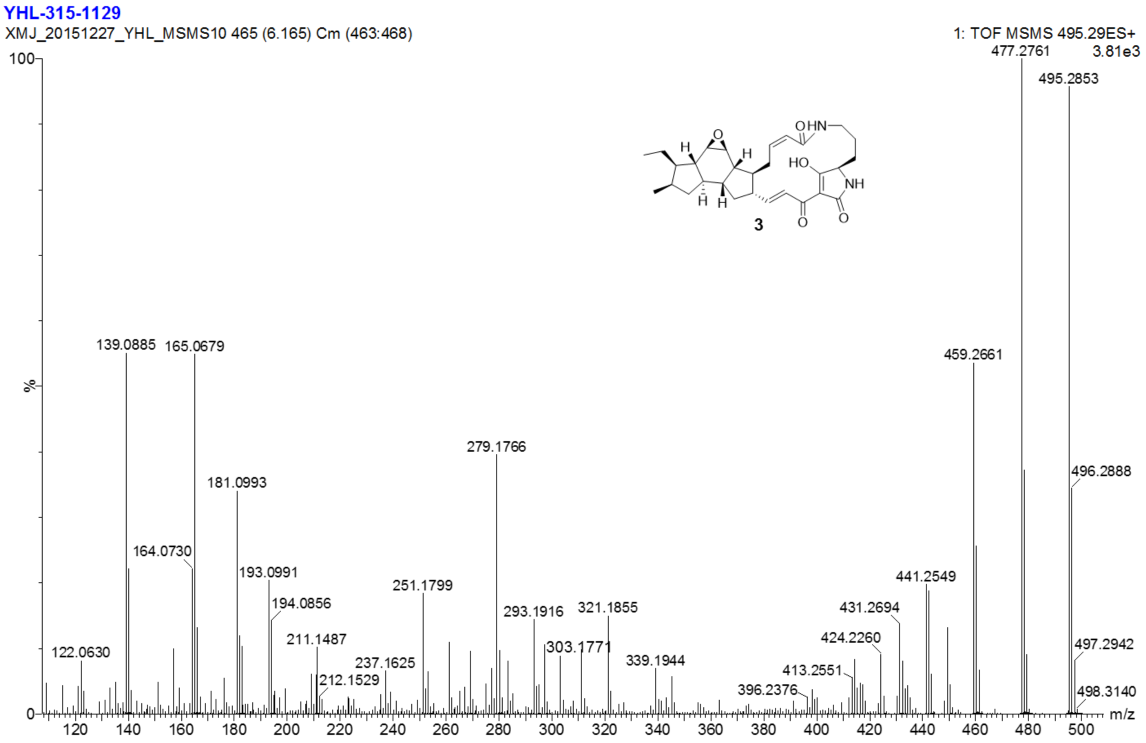

Figure S11. ESI-HR-MS/MS of **4**.

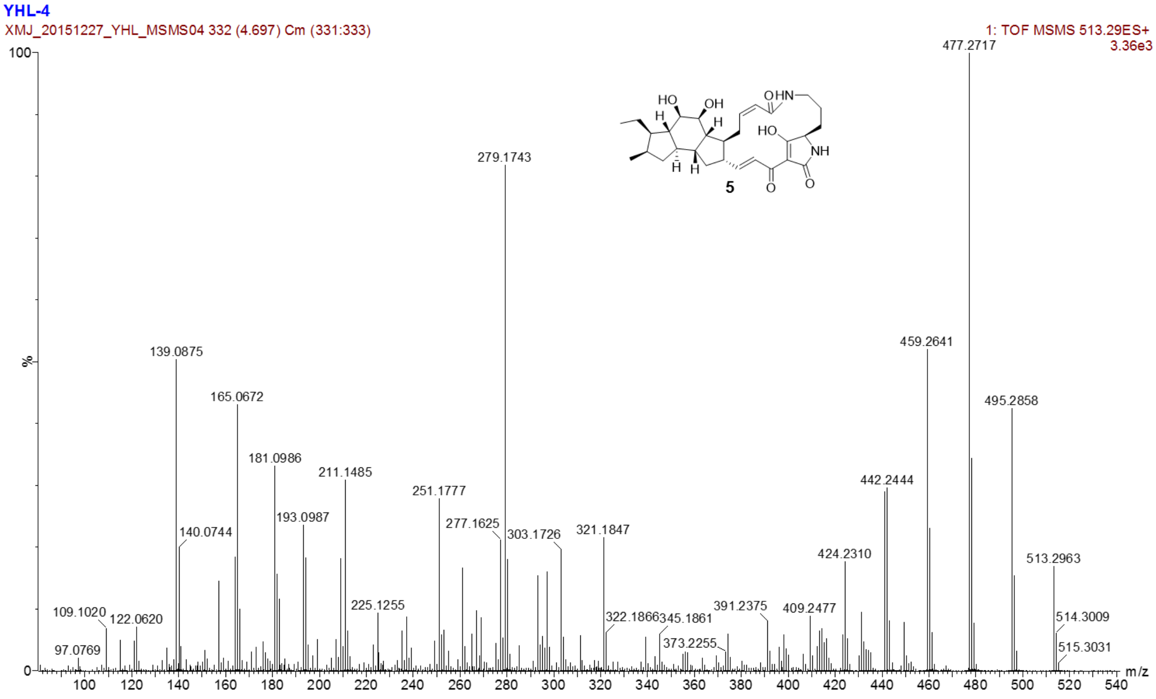

Figure S12. ESI-HR-MS/MS of **5**.

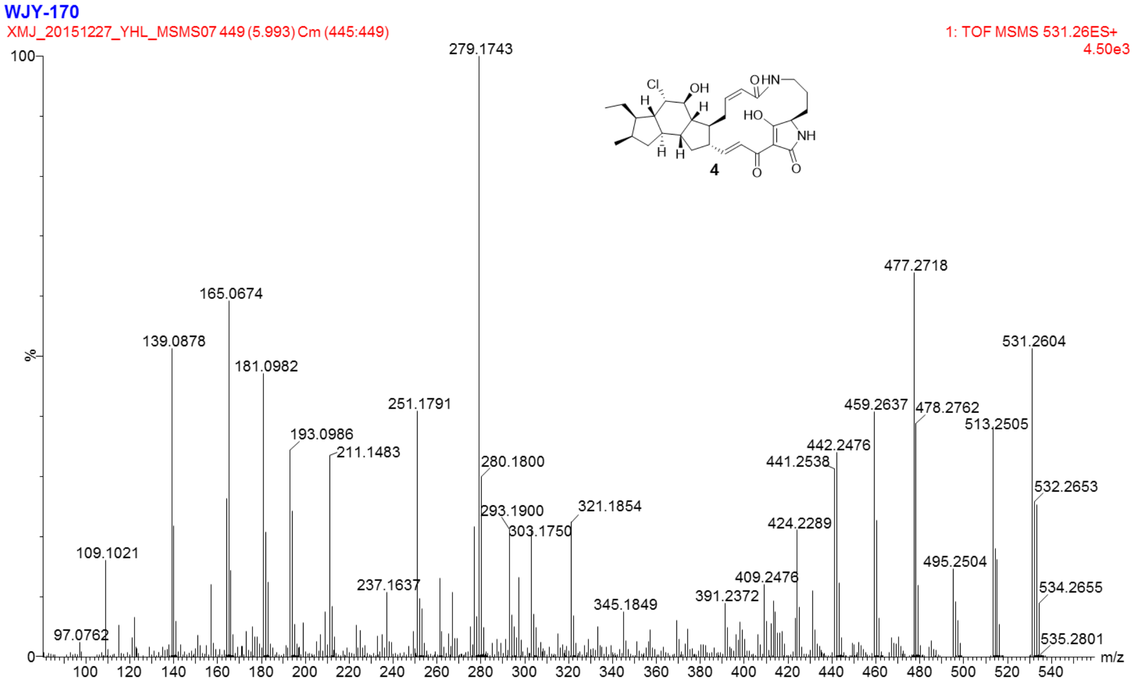

Figure S13. ESI-HR-MS/MS of **6**.

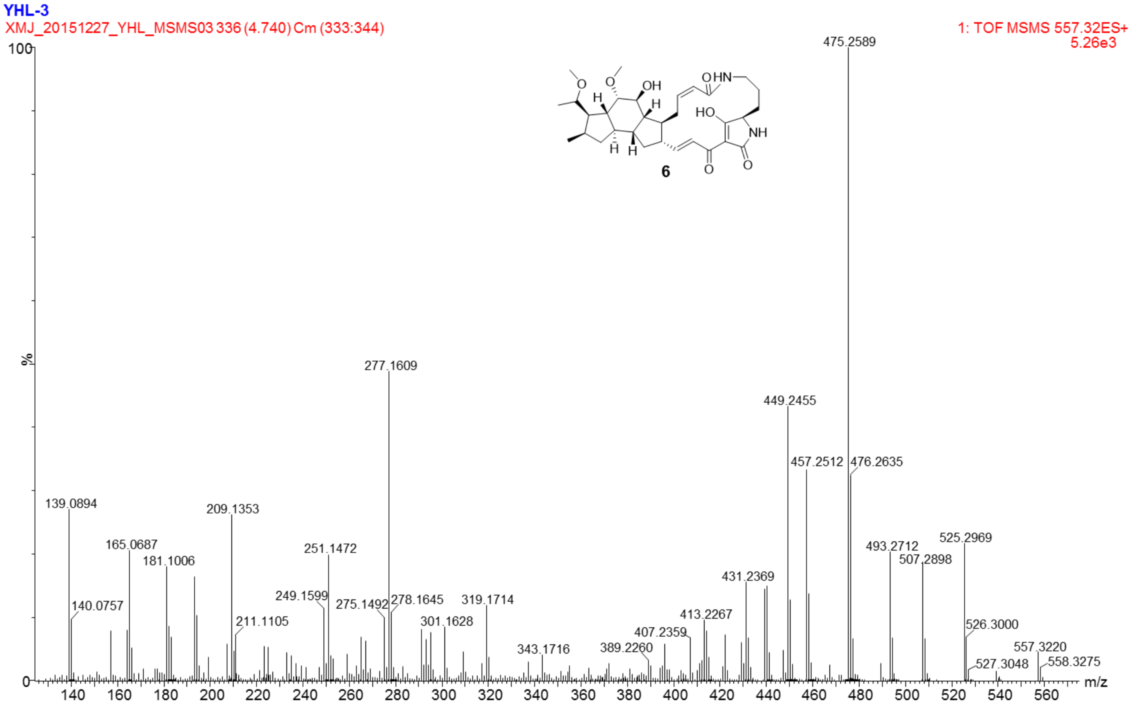

Figure S14. ESI-HR-MS/MS of **7**.

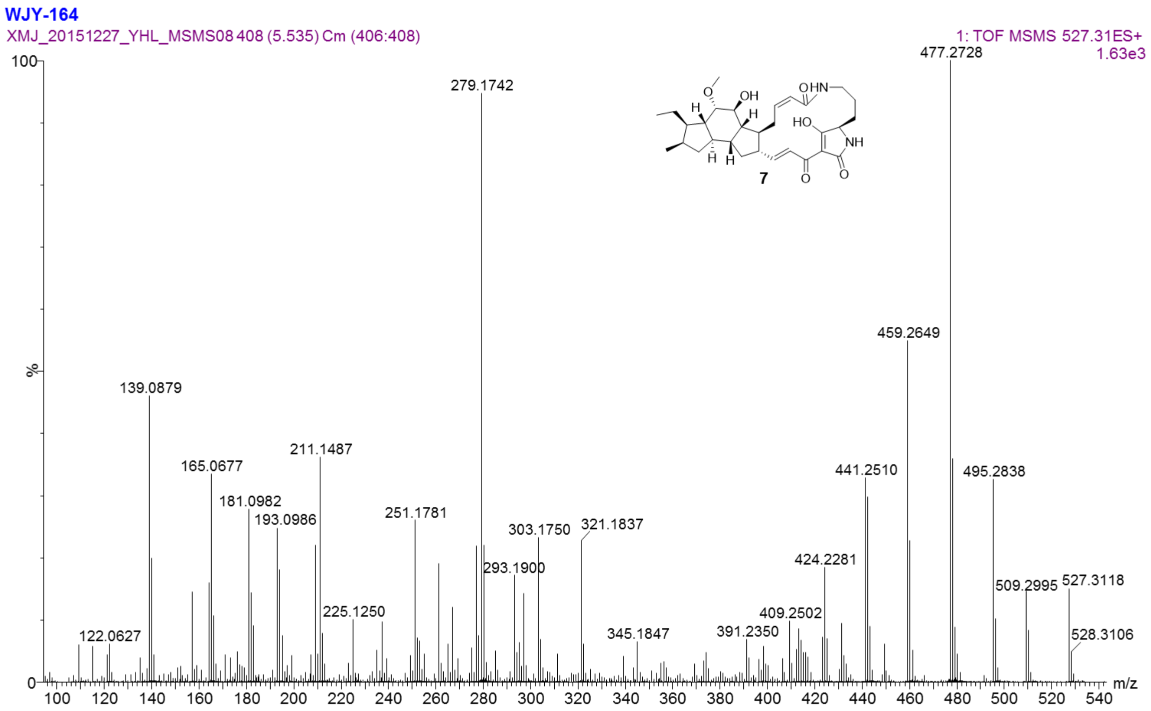

Figure S15. ESI-HR-MS/MS of **3'**.

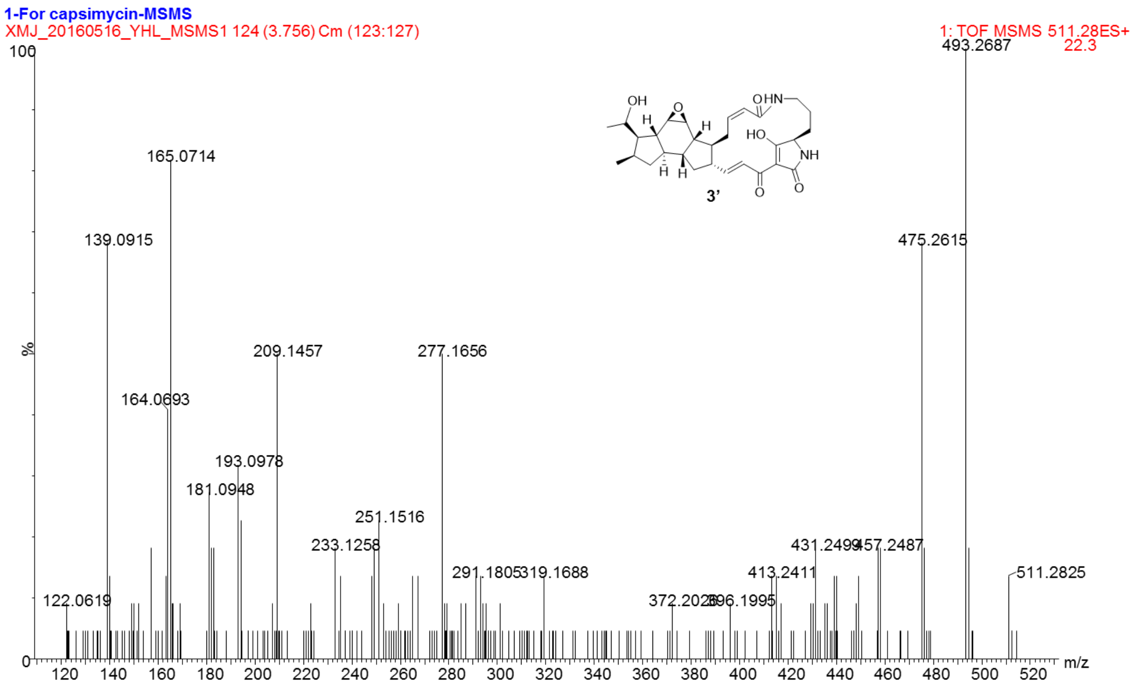

Figure S16. UV spectra of compounds **1-7**.

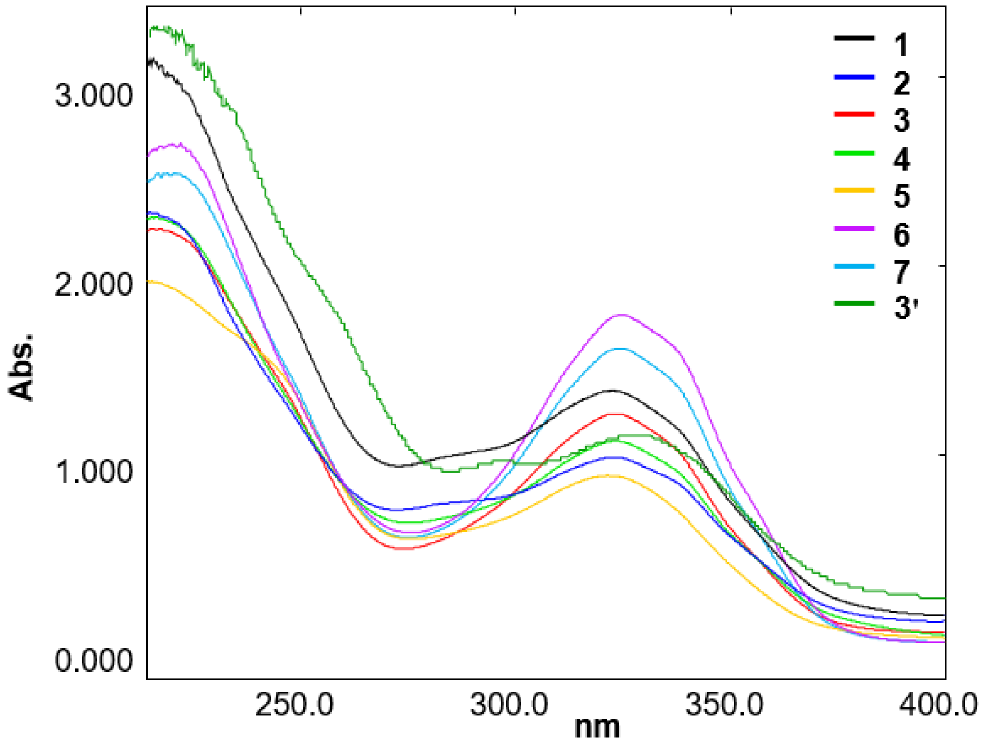

Table S3. ^{1}H (600 MHz) and ^{13}C (150 MHz) spectroscopic data of capsimycin (**2**).

Position	δ_C, type	δ_H, multi (*J* in H_Z)	HMBC (correlations from H to C)
1	196.5, C		
2	61.6, CH	3.83, br d (3.6)	1, 3, 4, 27
3	27.2, CH_2	1.75, m	1, 2, 4, 5
		1.97, m	
4	20.9, CH_2	1.19, m	2, 3, 5
		1.47, m	
5	38.9, CH_2	2.55, br t (10.4)	3, 4, 7
		3.61, m	
6-NH		6.39, br s	5, 7
7	166.3, C		
8	124.2, CH	5.82, dd (11.4, 2.0)	7, 9, 10
9	140.0, CH	6.04, ddd (11.4, 11.4, 2.3)	7, 8, 11
10	25.3, CH_2	2.36, m	8, 9, 11, 12
		3.67, m	
11	45.5, CH	1.65, m	9, 10, 12, 13, 23
12	40.1, CH	2.31, m	10, 11, 13, 14, 15, 20
13	53.8, CH	2.87, d (4.0)	11, 12, 14, 20
14	58.3, CH	3.22, dd (4.0, 2.1)	15
15	47.1, CH	0.95, m	14, 16, 20, 30
16	50.1, CH	1.87, m	14, 15, 17, 30, 31
17	33.6, CH	2.30, m	16, 18, 29, 30
18	39.0, CH_2	0.59, m	15, 16, 17, 19, 20, 29
		1.96, m	
19	47.5, CH	1.07, m	14, 15, 18, 20, 21
20	40.8, CH	1.67, m	11, 12, 18, 21
21	36.8, CH_2	1.11, m	11, 12, 19, 20, 22, 23
		2.07, m	
22	48.7, CH	2.44, m	10, 11, 21, 23, 24
23	151.2, CH	6.69, dd (15.4, 10.1)	11, 21, 22, 24, 25, 26
24	122.7, CH	7.11, d (15.4)	11, 22, 23, 25, 26
25	173.6, C		
26	100.7, C		
27	175.7, C		
28-NH		7.17, br s	
29	17.7, CH_3	0.93, d (7.2)	16, 17, 18
30	77.4, CH	3.30, m	16, 17, 33
31	17.4, CH_3	1.25, d (7.2)	16, 30

32	55.2, CH_3O	3.25, s	30

Measured in $CDCl_3$. δ values are given in ppm.

Figure S17. HRESIMS of capsimycin (**2**) (Positve mode).

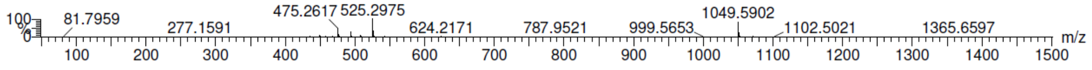

Minimum:				-1.5				
Maximum:		5.0	5.0	50.0				
Mass	Calc. Mass	mDa	PPM	DBE	i-FIT	Norm	Conf(%)	Formula
525.2975	525.2965	1.0	1.9	11.5	256.0	0.427	65.26	C30 H41 N2 O6
	525.2983	-0.8	-1.5	-1.5	256.6	1.057	34.74	C18 H45 N4 O13

Figure S18. ^{1}H NMR (600 MHz, $CDCl_3$) spectrum of capsimycin (**2**).

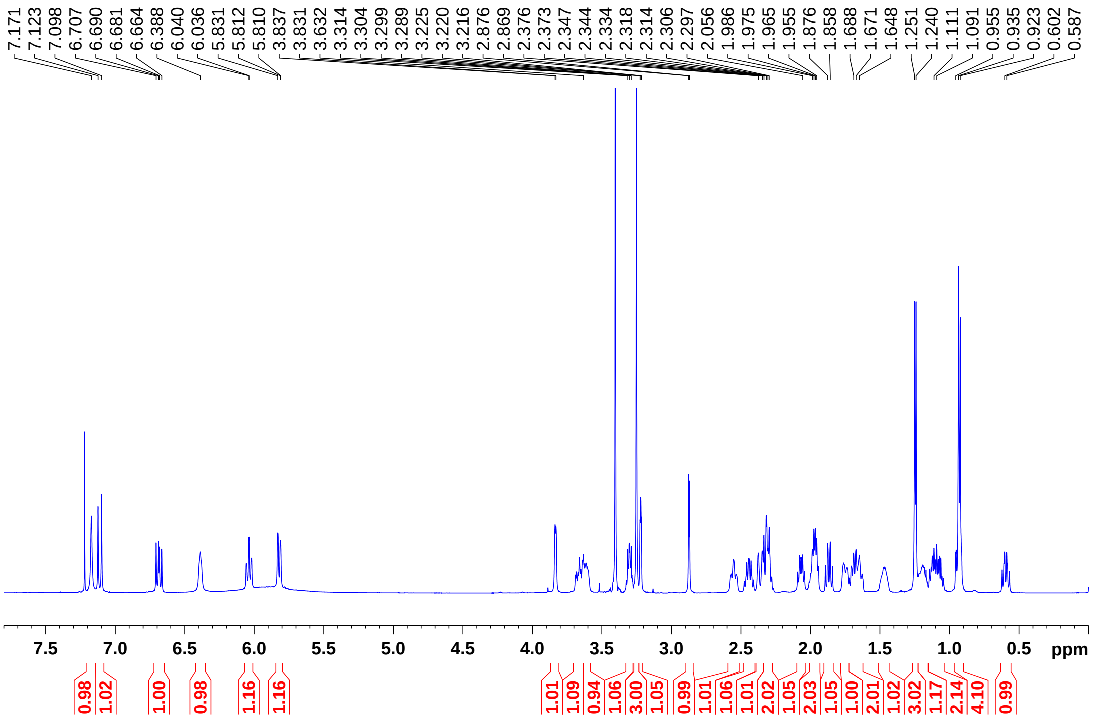

Figure S19. ^{13}C NMR (600 MHz, $CDCl_3$) spectrum of capsimycin (**2**).

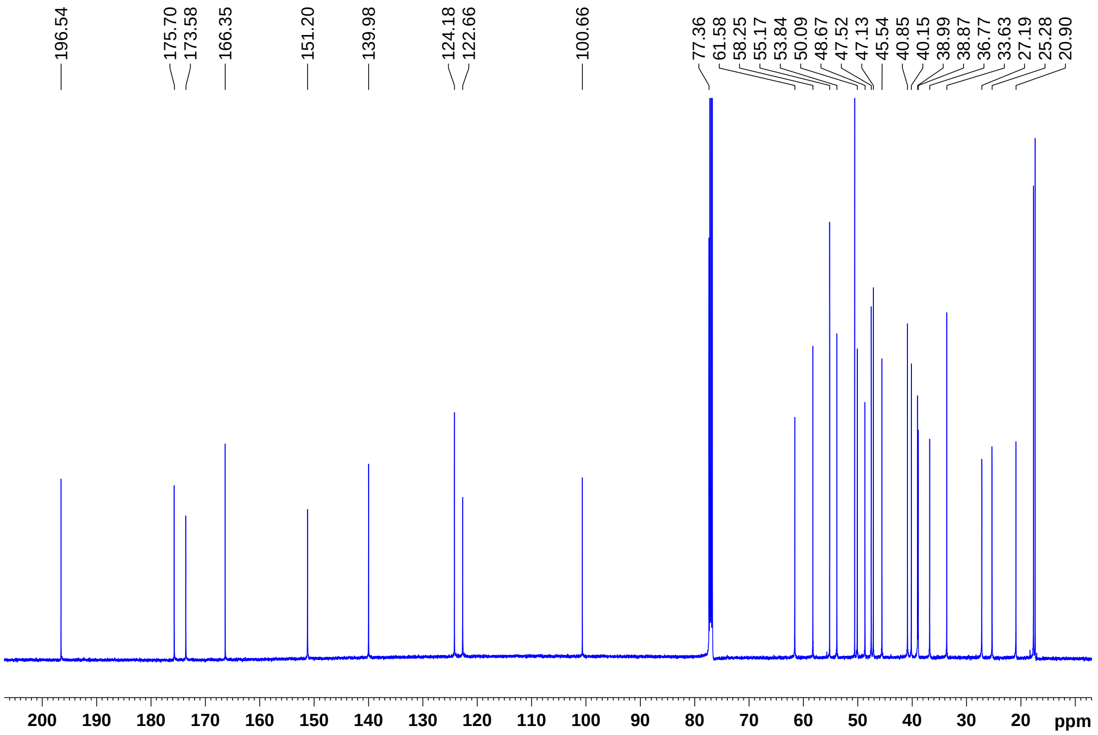

Figure S20. DEPT 135 (600 MHz, $CDCl_3$) spectrum of capsimycin (**2**).

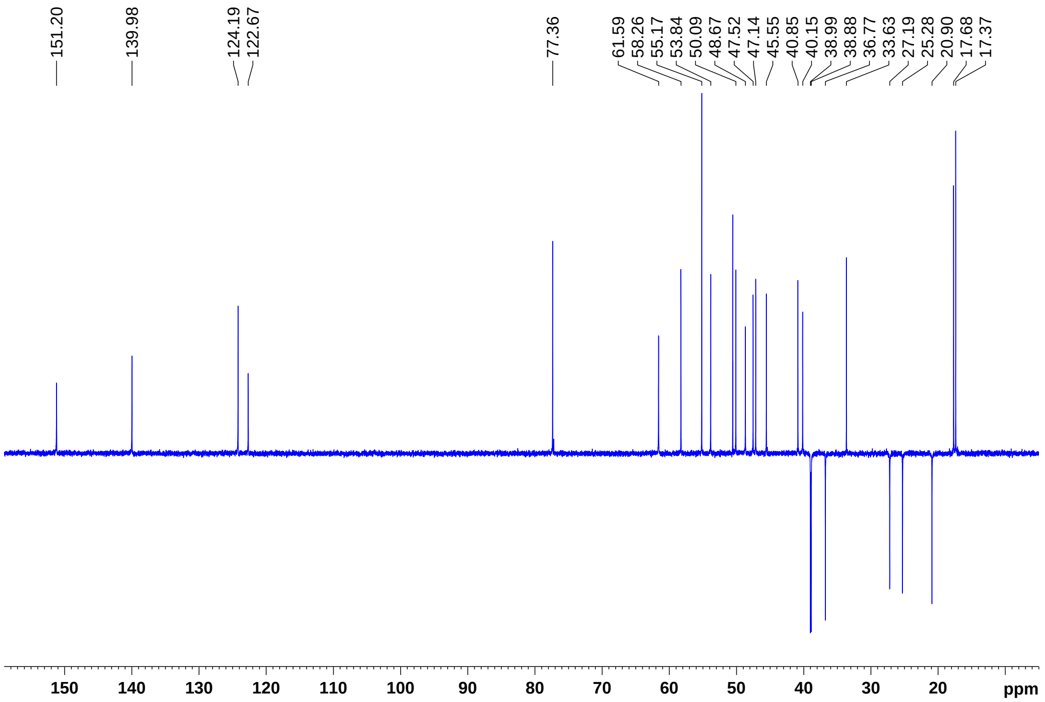

Figure S21. HSQC (600 MHz, $CDCl_3$) of capsimycin (**2**).

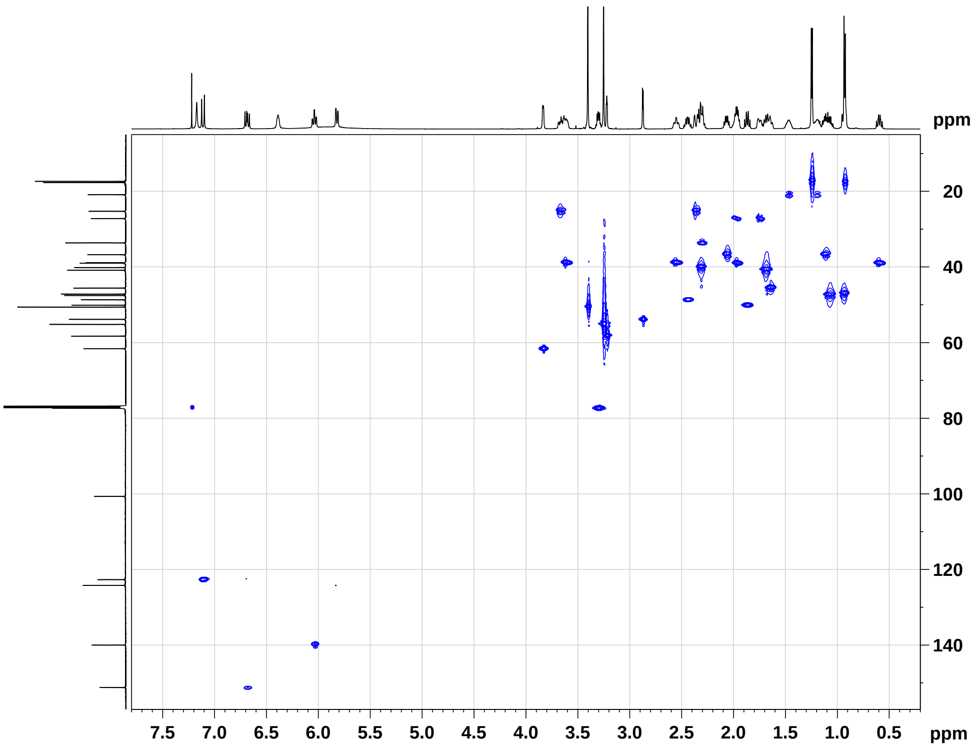

Figure S22. HMBC (600 MHz, $CDCl_3$) of capsimycin (**2**).

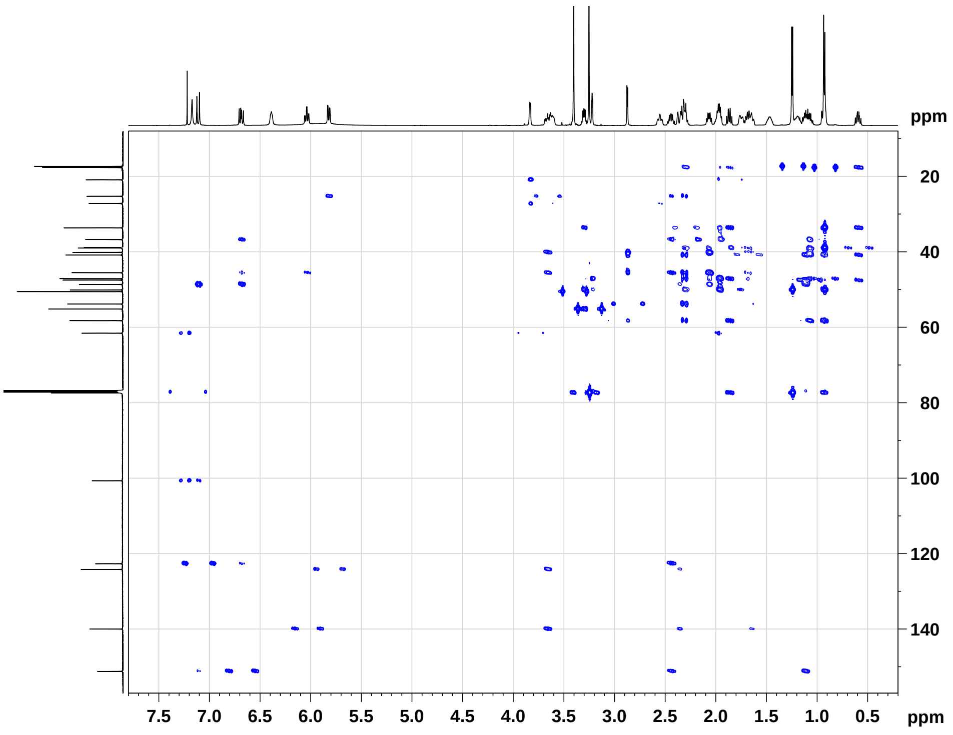

Figure S23. COSY (600 MHz, $CDCl_3$) of capsimycin (**2**).

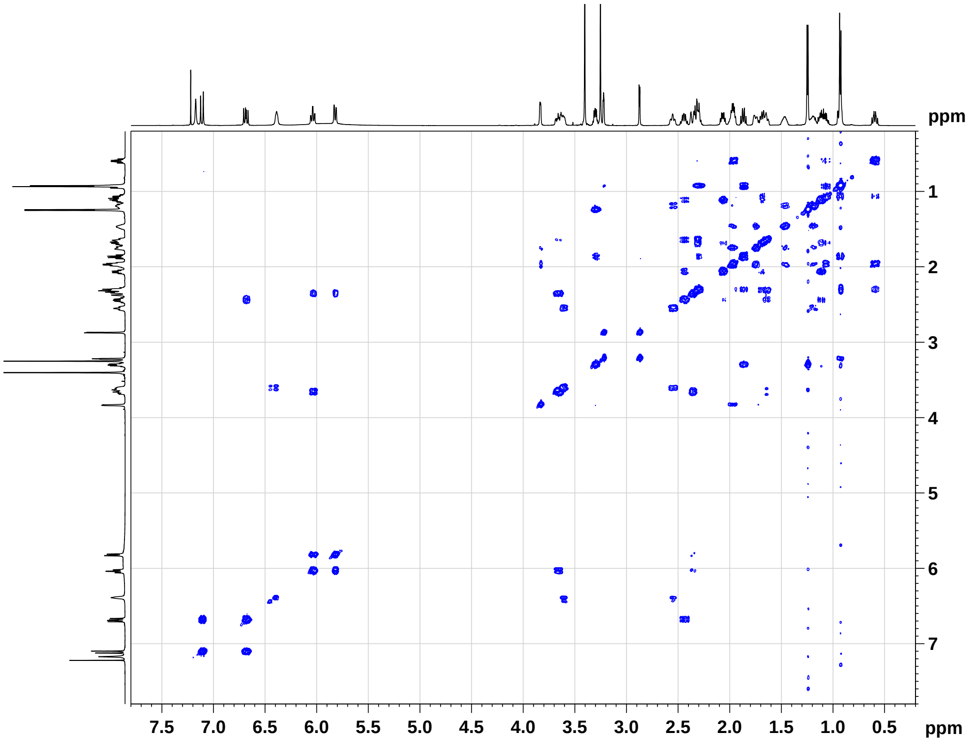

Figure S24-a. NOESY (600 MHz, $CDCl_3$) of capsimycin (**2**).

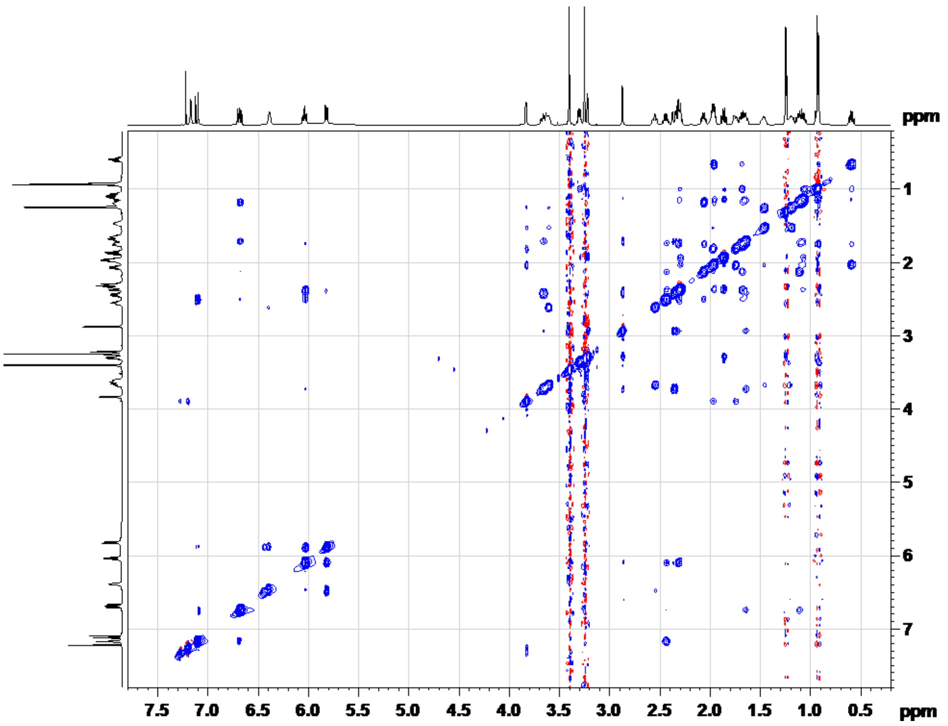

Figure S24-b. ROESY (600 MHz, $CDCl_3$) of capsimycin (**2**).

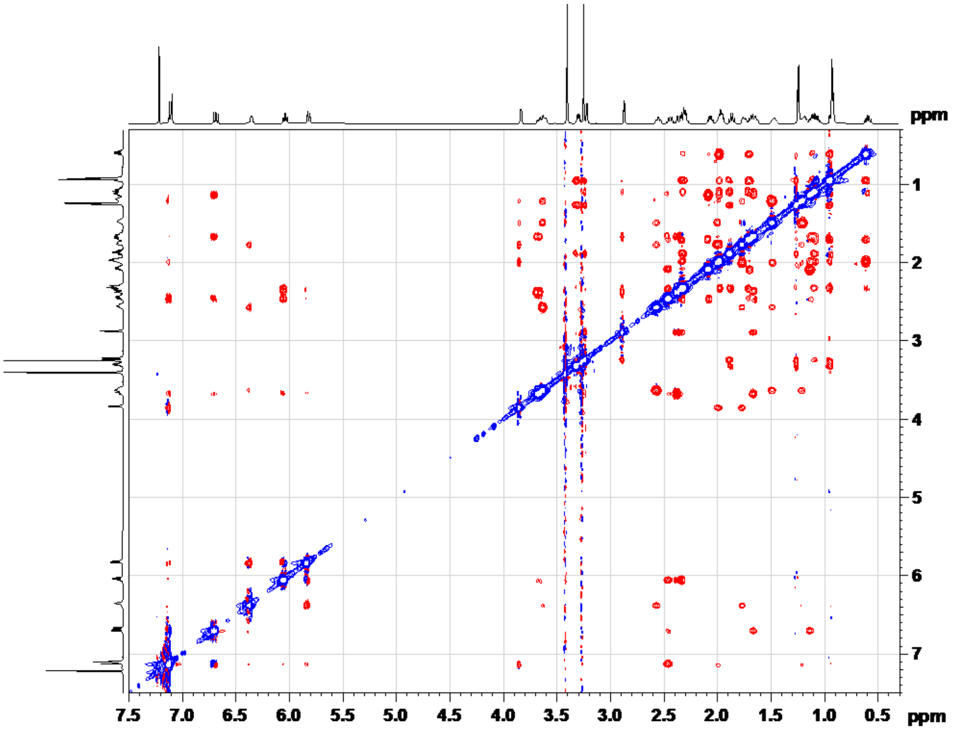

Figure S25. HRESIMS of **3** (Positve mode).

Single Mass Analysis
Tolerance = 10.0 PPM / DBE: min = -1.5, max = 50.0
Element prediction: Off
Number of isotope peaks used for i-FIT = 3

Monoisotopic Mass, Even Electron Ions
340 formula(e) evaluated with 3 results within limits (up to 50 best isotopic matches for each mass)
Elements Used:
C: 0-35 H: 0-100 N: 0-5 O: 0-10
3151
XMJ_20151227_YHL_03 288 (6.165) Cm (287:288)
1: TOF MS ES+
6.07e+004

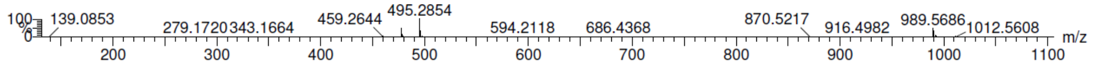

Mass	Calc. Mass	mDa	PPM	DBE	i-FIT	Norm	Conf(%)	Formula
Minimum:				-1.5				
Maximum:		5.0	10.0	50.0				
495.2854	495.2859	-0.5	-1.0	11.5	214.3	0.980	37.55	C29 H39 N2 O5
	495.2819	3.5	7.1	7.5	214.0	0.672	51.07	C24 H39 N4 O7
	495.2899	-4.5	-9.1	15.5	215.5	2.173	11.39	C34 H39 O3

Table S4. ^{1}H (600 MHz) and ^{13}C (150 MHz) spectroscopic data of **3**.

Position	δ_C, type	δ_H, multi (*J* in Hz)	HMBC (correlations from H to C)
1	195.9, C		
2	61.4, CH	3.85, br d (3.7)	1, 3, 4, 27
3	27.4, CH_2	1.77, m	1, 2, 4, 5
		1.98, m	
4	21.0, CH_2	1.20, m	2, 3, 5
		1.51, m	
5	38.9, CH_2	2.56, br t (11.4)	3, 4, 7
		3.63, m	
6-NH			
7	166.3, C		
8	124.2, CH	5.79, dd (11.6, 2.0)	7, 9, 10
9	140.4, CH	6.06, ddd (11.6, 11.6, 2.5)	7, 10, 11
10	25.5, CH_2	2.38, m	8, 9, 11, 22
		3.61, m	
11	45.2, CH	1.65, m	9, 10, 12, 13, 23
12	40.8, CH	2.34, m	10, 11, 13, 14, 19, 20
13	53.5, CH	2.89, d (3.8)	11, 12, 14, 20
14	57.7, CH	3.13, dd (3.8, 2.0)	15, 16
15	49.6, CH	0.91, m	14, 16, 20, 30
16	46.6, CH	1.60, m	14, 15, 17, 29, 30, 31
17	33.0, CH	2.18, m	15, 16, 18, 29
18	38.3, CH_2	0.59, m	15, 16, 17, 19, 20, 29
		1.96, m	
19	47.9, CH	1.01, m	14, 15, 18, 20, 21
20	40.7, CH	1.70, m	11, 12, 15, 18, 19, 21
21	36.6, CH_2	1.11, m	11, 12, 19, 20, 22
		2.06, m	
22	48.9, CH	2.46, m	10, 11, 21, 23, 24
23	151.6, CH	6.70, dd (15.6, 10.2)	11, 21, 22, 24, 25
24	122.7, CH	7.11, d (15.6)	11, 21, 22, 23, 25, 26
25	173.7, C		
26	100.6, C		
27	175.5, C		
28-NH			
29	17.5, CH_3	0.81, d (7.3)	16, 17, 18
30	22.5, CH_2	1.44, m	15, 16, 17, 31
31	13.2, CH_3	0.94, d (7.3)	16, 30

Measured in $CDCl_3$. δ values are given in ppm.

Figure S26. ^{1}H NMR (600 MHz, $CDCl_3$) spectrum of **3**.

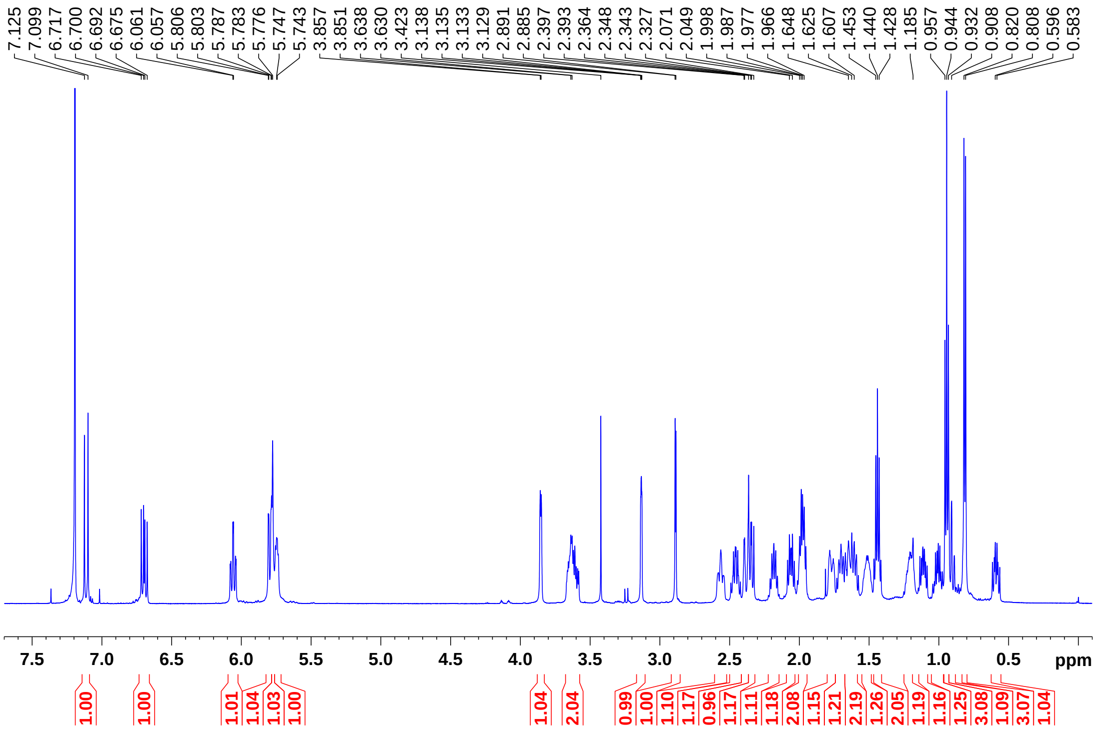

Figure S27. ^{13}C NMR (600 MHz, $CDCl_3$) spectrum of **3**.

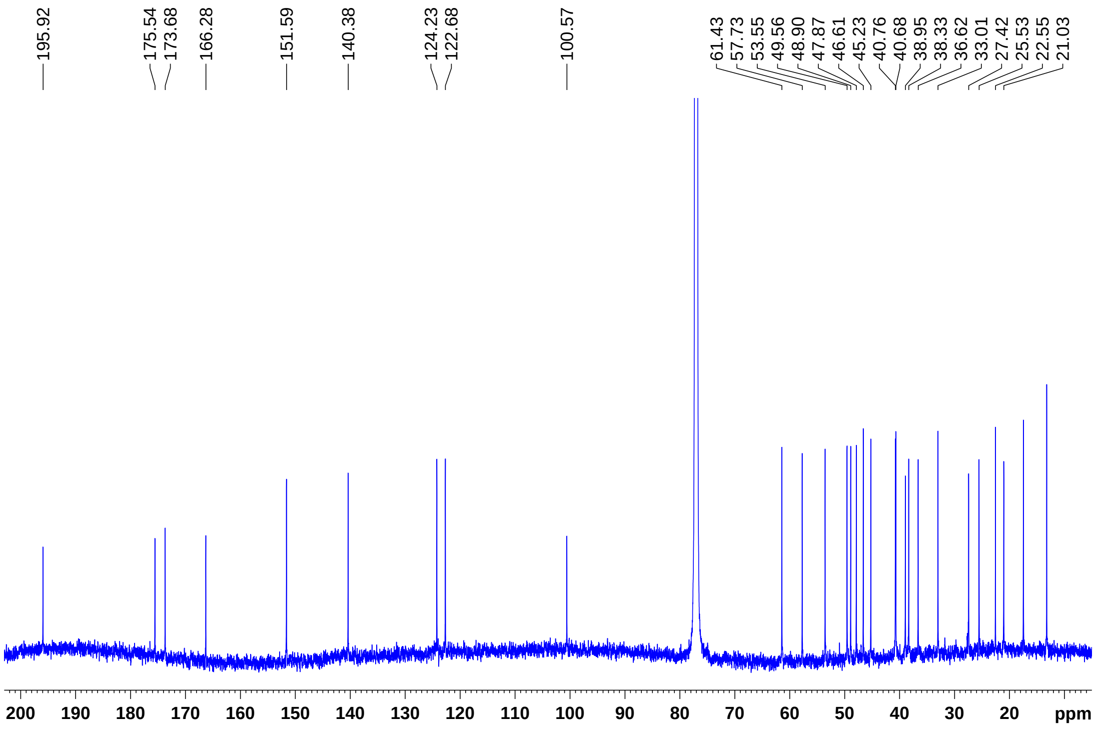

Figure S28. ^{13}C-DEPT 135 (600 MHz, $CDCl_3$) spectrum of **3**.

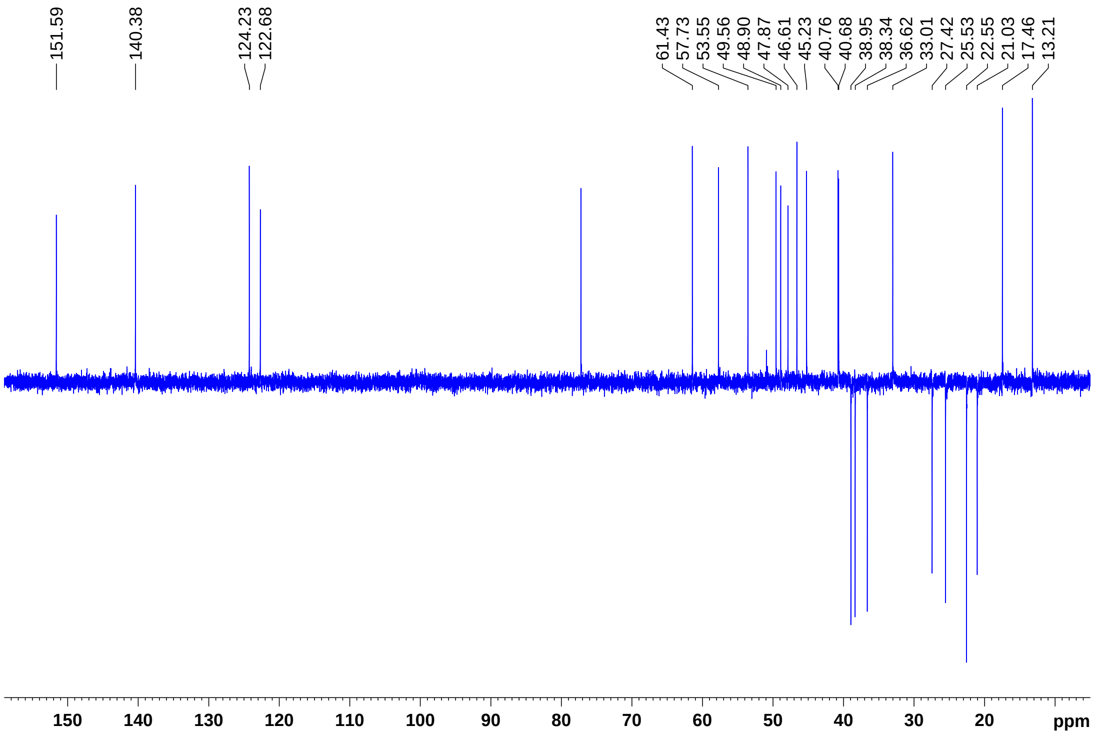

Figure S29. HSQC (600 MHz, $CDCl_3$) of **3**.

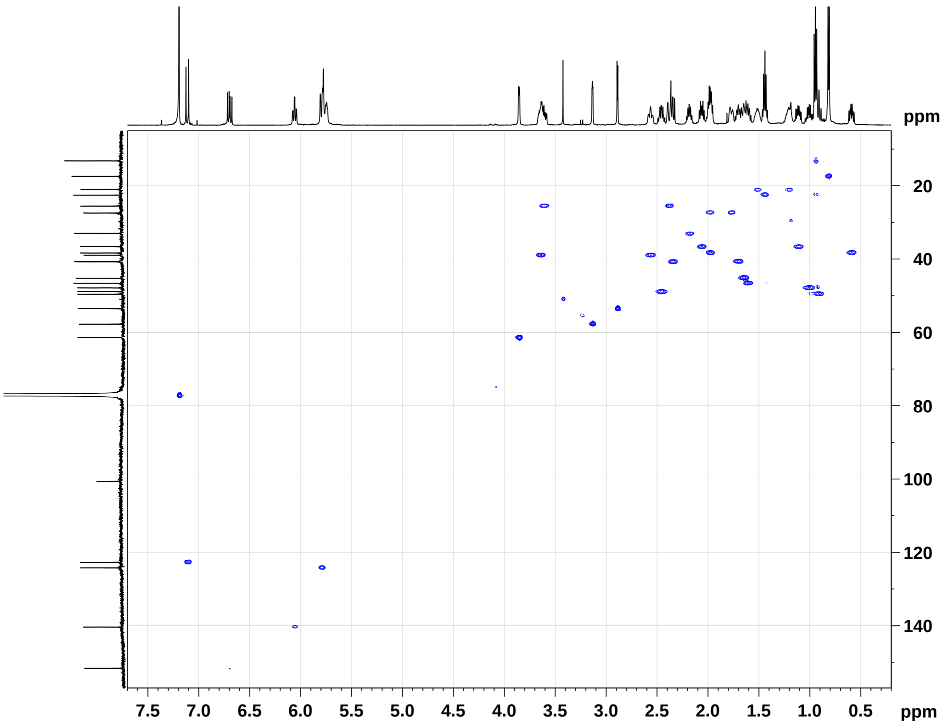

Figure S30. HMBC (600 MHz, $CDCl_3$) of **3**.

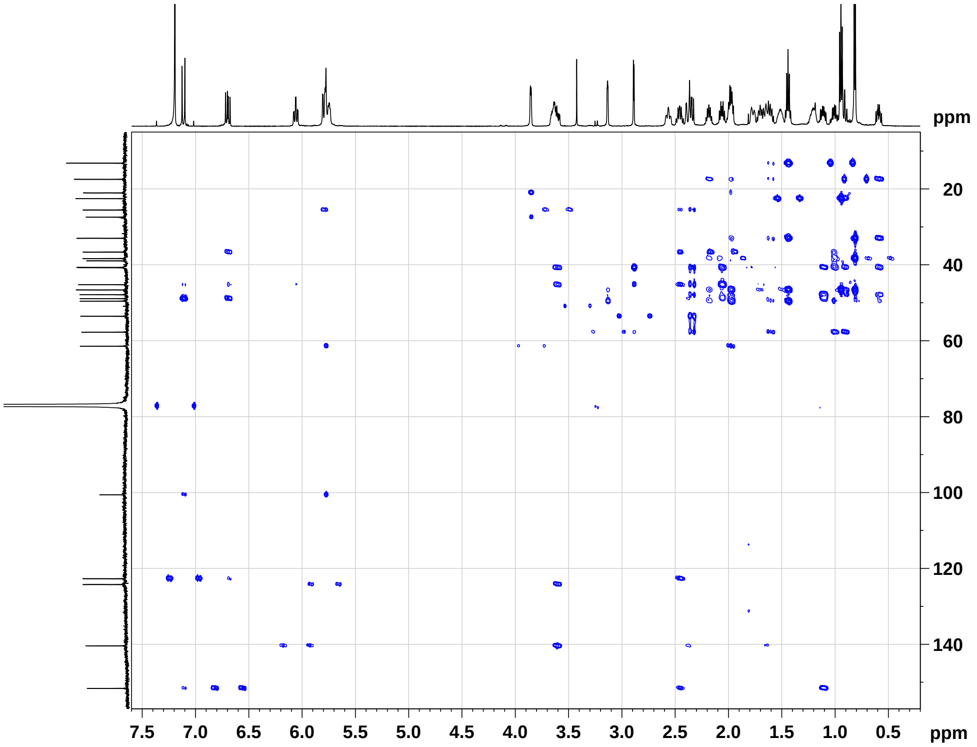

Figure S31. COSY (600 MHz, $CDCl_3$) of **3**.

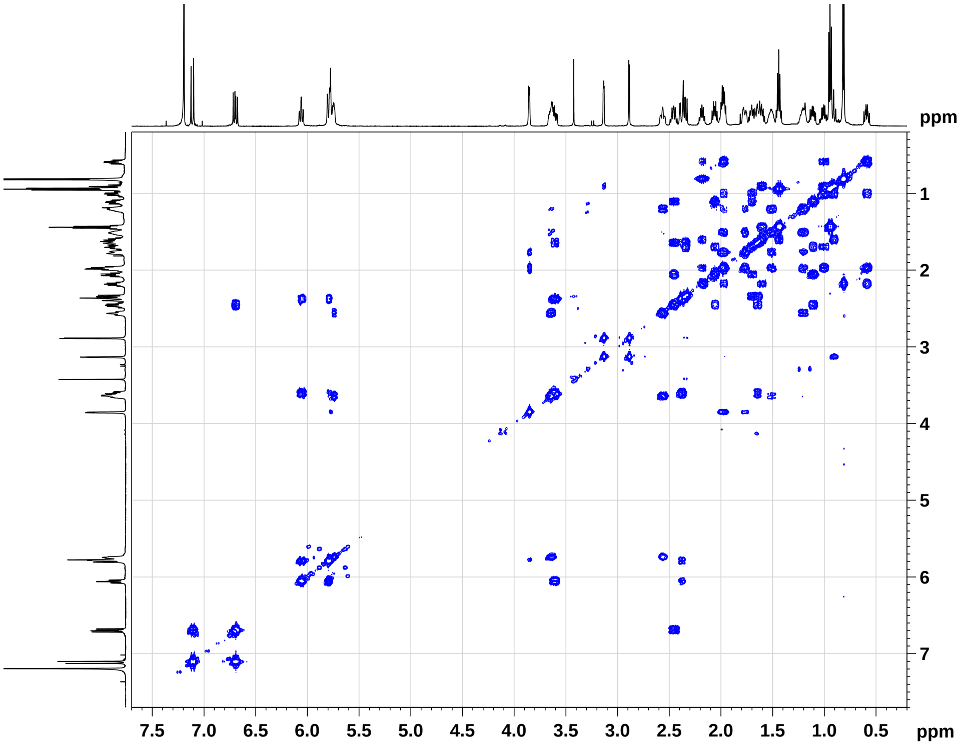

Figure S32. NOESY (600 MHz, $CDCl_3$) of **3**.

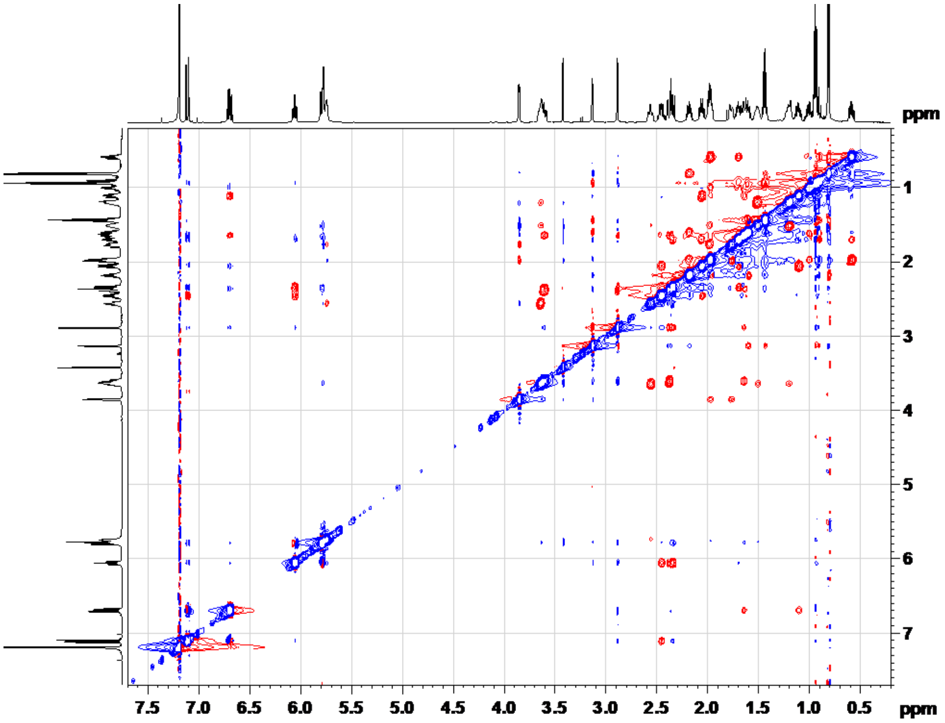

Figure S33. HRESIMS of **4** (Positve mode).

Elemental Composition Report **Page 1**

Single Mass Analysis
Tolerance = 5.0 mDa / DBE: min = -1.5, max = 50.0
Element prediction: Off
Number of isotope peaks used for i-FIT = 3

Monoisotopic Mass, Even Electron Ions
195 formula(e) evaluated with 2 results within limits (up to 50 best isotopic matches for each mass)
Elements Used:
C: 24-35 H: 6-100 N: 2-5 O: 2-20
100-1-12
XMJ_20151103_06 218 (4.683) Cm (218) 1: TOF MS ES+
1.83e+004

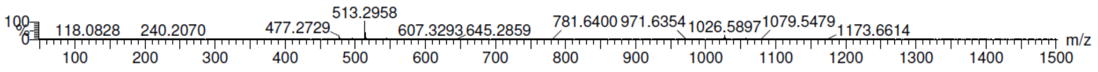

Minimum:				-1.5				
Maximum:		5.0	10.0	50.0				
Mass	Calc. Mass	mDa	PPM	DBE	i-FIT	Norm	Conf(%)	Formula
513.2958	513.2965	-0.7	-1.4	10.5	26.3	0.044	95.72	C29 H41 N2 O6
	513.2924	3.4	6.6	6.5	29.4	3.151	4.28	C24 H41 N4 O8

Table S5. ^{1}H (600 MHz) and ^{13}C (150 MHz) spectroscopic data of **4**.

Position	δ_C, type	δ_H (multi., *J* in Hz)	HMBC (correlations from H to C)
1	197.5, C		
2	61.8, CH	3.79, dd (5.5, 2.1)	1, 3, 4, 27
3	27.5, CH_2	1.71, m	1, 2, 4, 5
		1.96, m	
4	21.3, CH_2	1.06, m	3, 5
		1.51, m	
5	39.2, CH_2	2.54, br t (11.5)	3, 4, 7
		3.50, ddd (11.5, 4.6, 2.9)	
6-NH			
7	167.7, C		
8	123.6, CH	5.75, dd (11.6, 1.9)	7, 10
9	142.3, CH	5.99, dd (11.6, 3.1)	7, 11
10	26.9, CH_2	2.40, ddd (17.5, 3.0, 3.0)	8, 9, 11, 12
		3.29, m	
11	46.0, CH	1.97, m	12, 20, 22
12	46.6, CH	1.91, dd (10.6, 4.0)	11, 13, 14, 19, 20
13	74.6, CH	3.72, br t (3.8)	12, 14, 15, 19, 20
14	72.1, CH	3.74, br t (3.8)	12, 13, 15
15	48.0, CH	1.38, dd (11.0, 3.6)	16, 19, 30
16	42.5, CH	1.68, m	17, 18, 20, 29, 31
17	33.3, CH	2.10, m	15, 16, 18, 19, 29
18	39.5, CH_2	0.61, m	15, 16, 17, 19, 20, 29
		2.05, dd (12.3, 7.5)	
19	41.0, CH	1.51, m	18, 20
20	43.5, CH	1.90, m	13, 18, 19
21	36.1, CH_2	1.20, m	11, 19, 20, 22
		1.99, m	
22	49.9, CH	2.33, m	11, 21, 23, 24
23	153.4, CH	6.75, dd (15.4, 10.3)	11, 21, 22, 25
24	122.3, CH	7.03, d (15.4)	22, 23, 25, 26
25	173.7, C		
26	101.0, C		
27	175.8, C		
28-NH			
29	17.6, CH3	0.81, d (7.0)	16, 17, 18
30	21.9, CH2	1.27, m	16, 17, 31
31	13.0, CH3	0.86, t (7.38)	16, 30

Measured in 90% $CDCl_3/CD_3OD$. δ values are given in ppm.

Figure S34. ^{1}H NMR (600 MHz, MeOD/CDCl$_3$) spectrum of **4**.

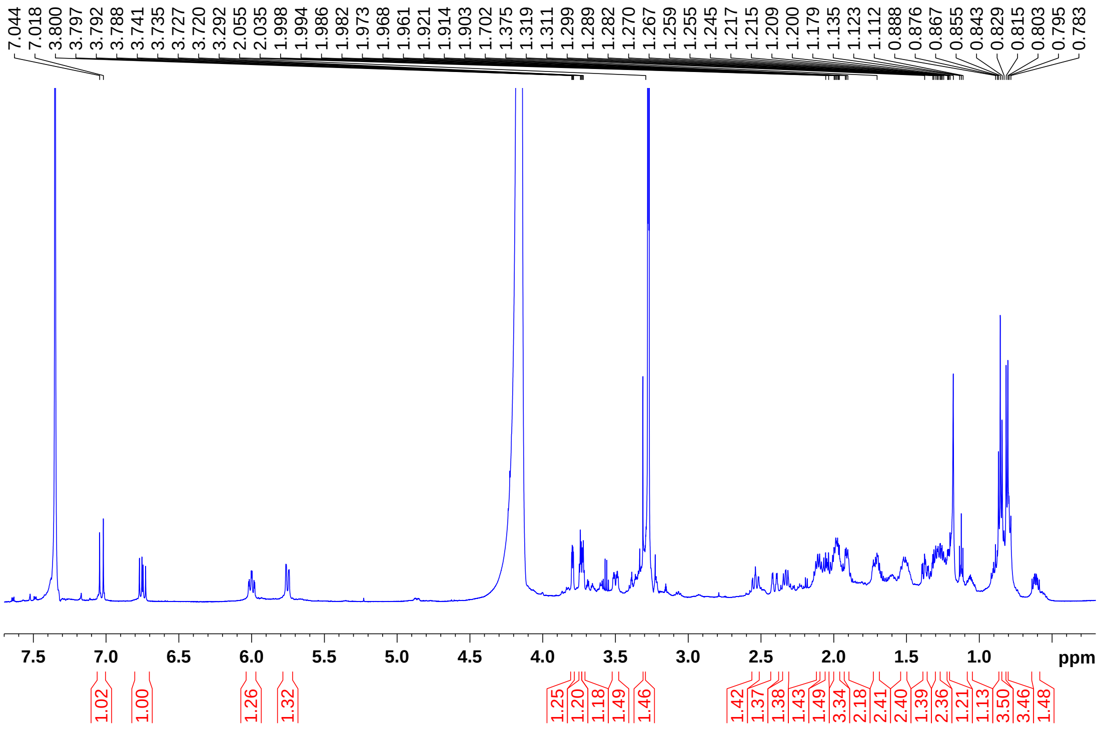

Figure S35. ^{13}C NMR (600 MHz, MeOD/CDCl$_3$) spectrum of **4**.

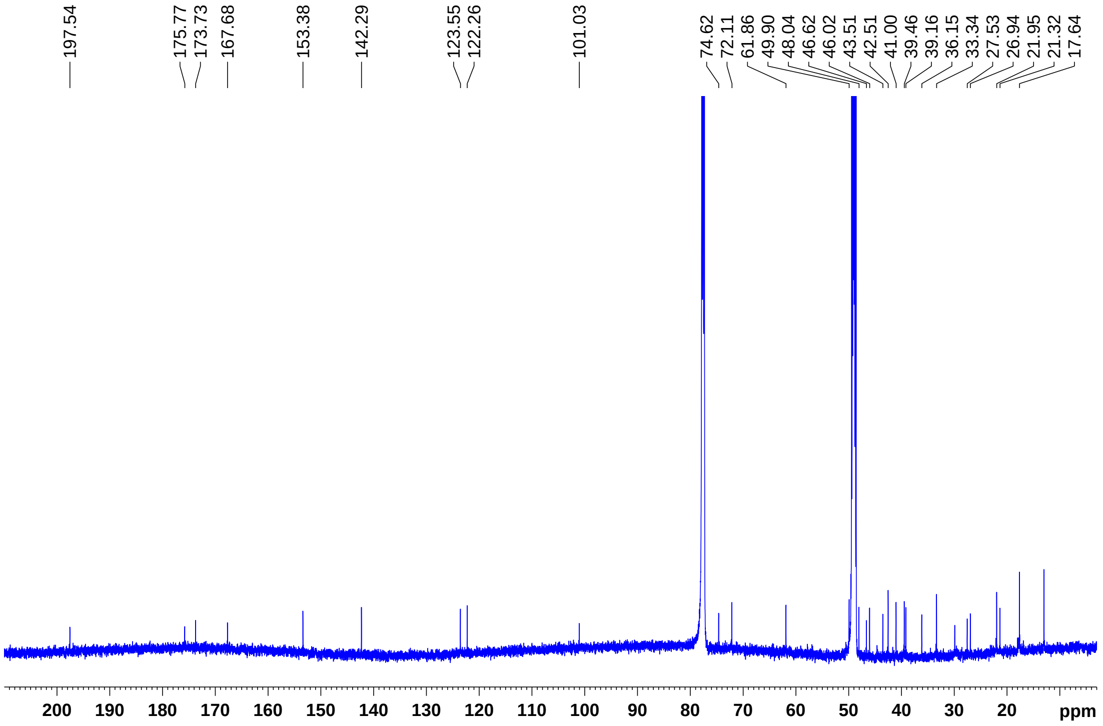

Figure S36. ^{13}C-DEPT NMR (600 MHz, MeOD/$CDCl_3$) spectrum of **4**.

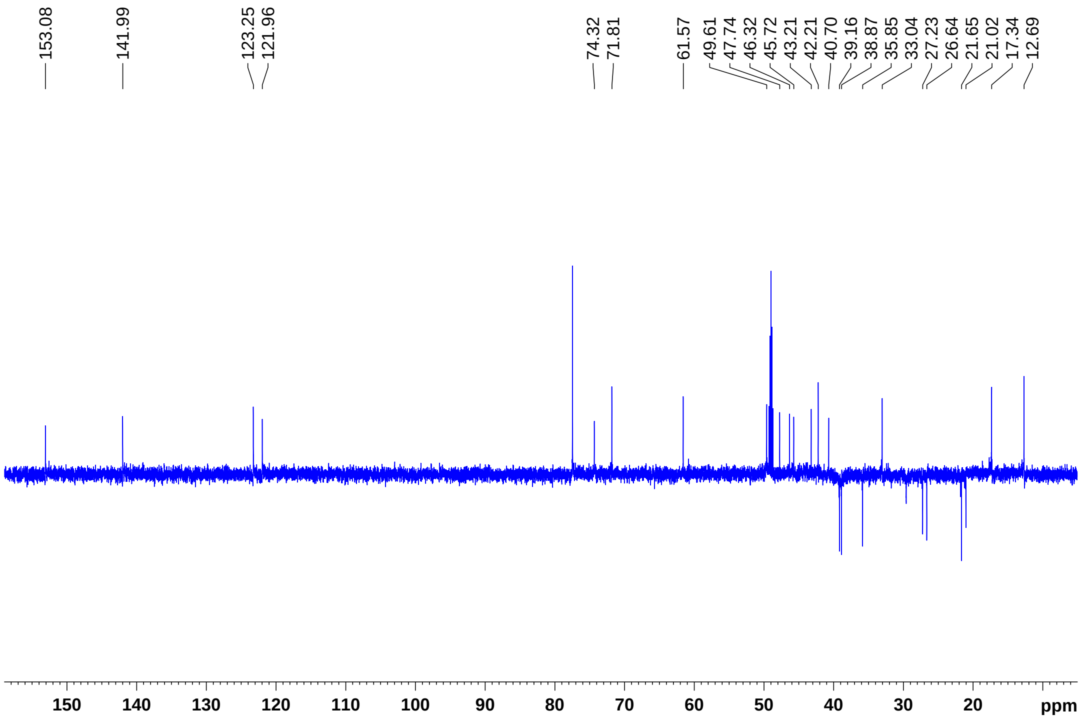

Figure S37. HSQC (600 MHz, MeOD/$CDCl_3$) of **4**.

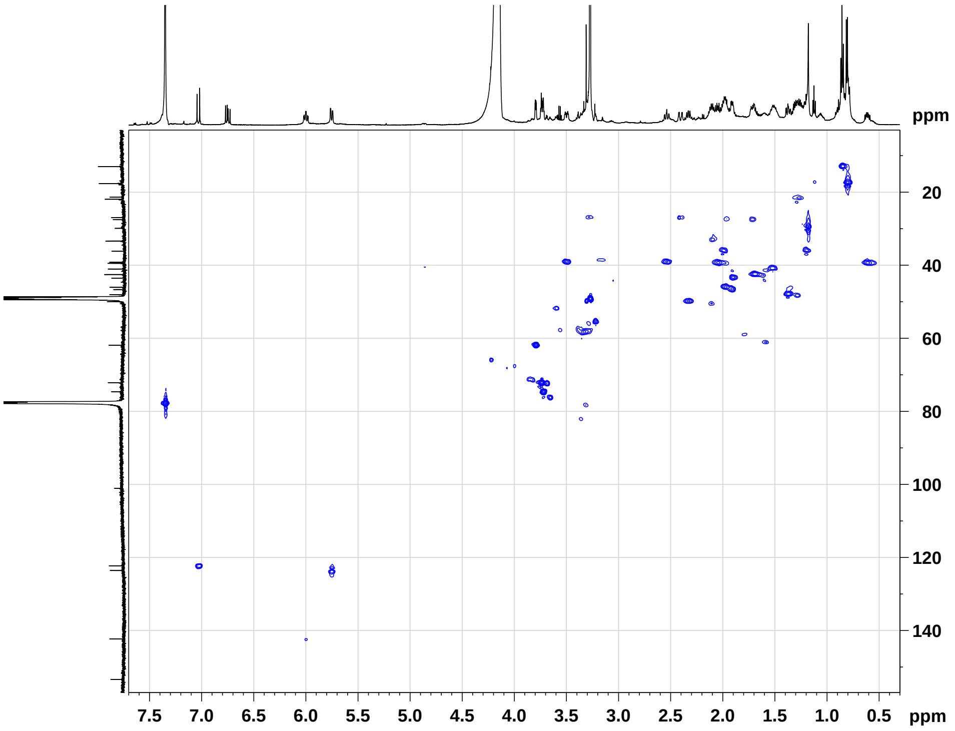

Figure S38. HMBC (600 MHz, MeOD/$CDCl_3$) of **4**.

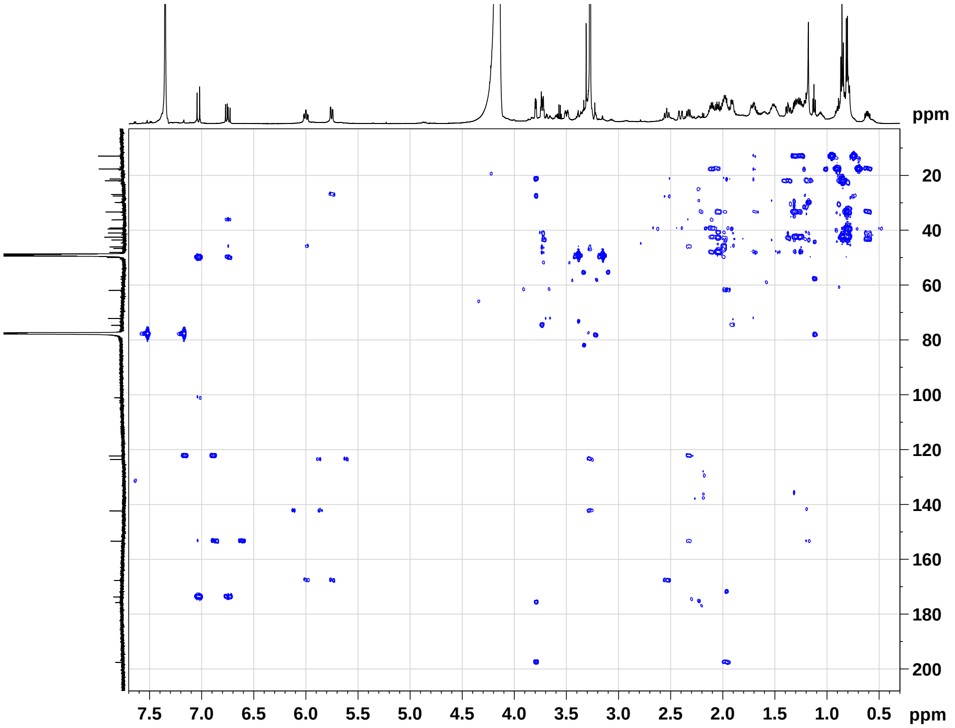

Figure S39. COSY (600 MHz, MeOD/$CDCl_3$) of **4**.

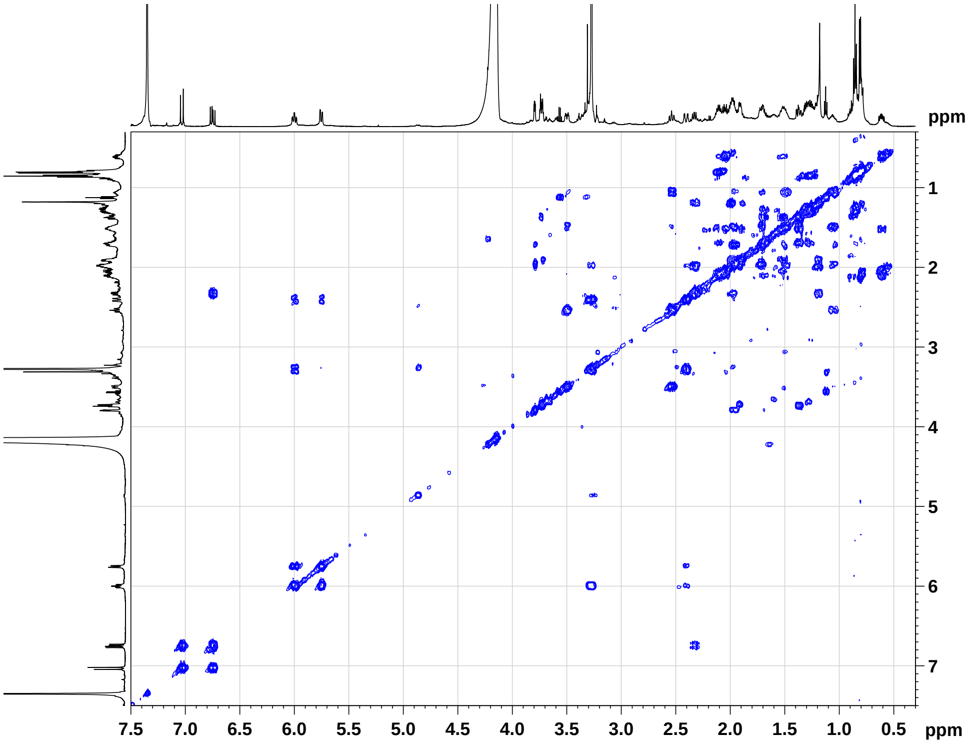

Figure S40. TOCSY (600 MHz, MeOD/$CDCl_3$) of **4**.

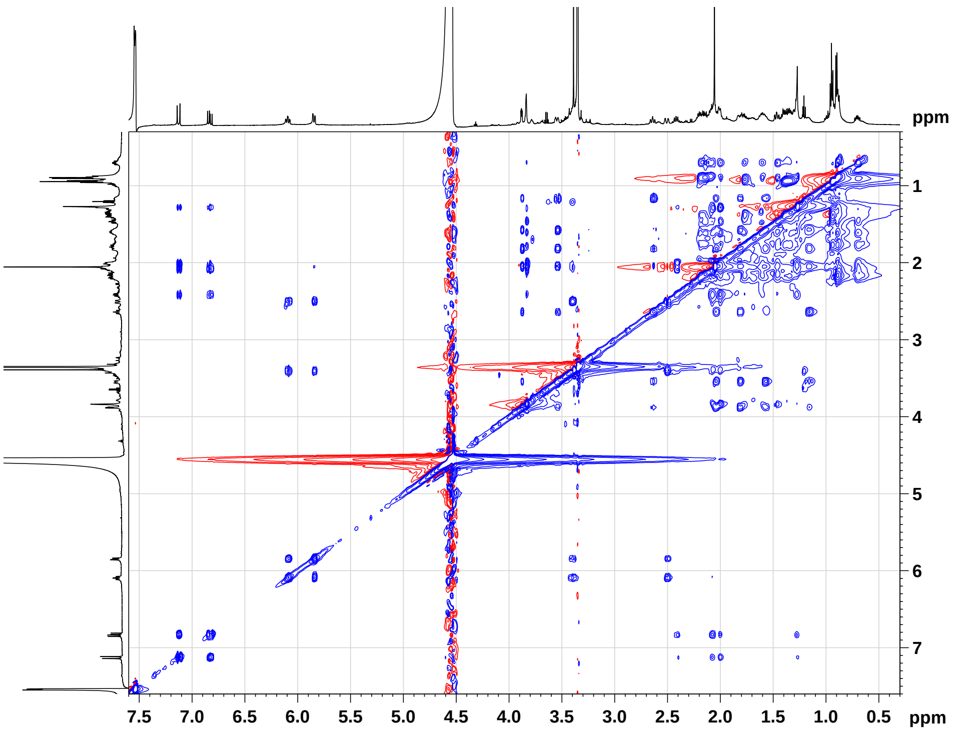

Figure S41-a. NOESY (600 MHz, MeOD/$CDCl_3$) of **4**.

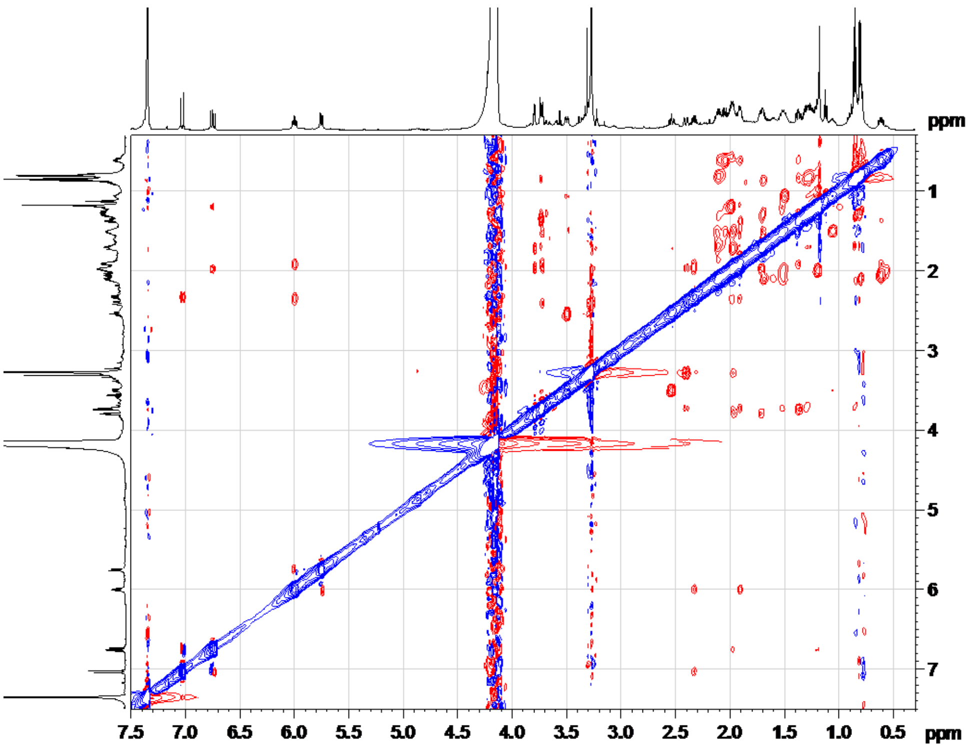

Figure S41-b. ROESY (600 MHz, MeOD/$CDCl_3$) of **4**.

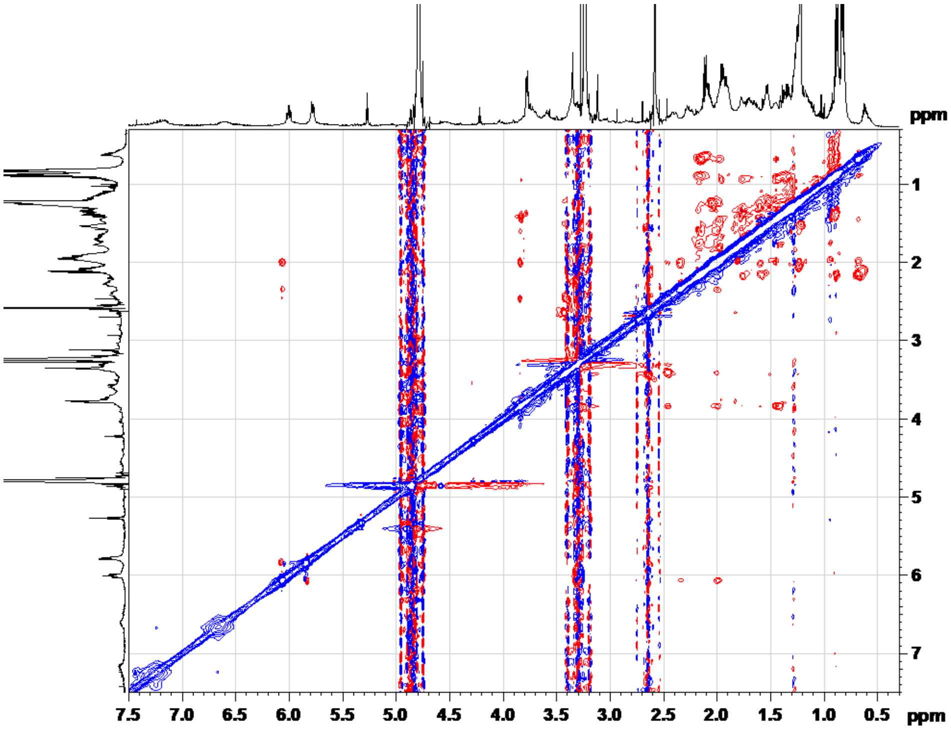

Figure S42-a. FT-MS of **5** (Positve mode).

Mass Spectrum SmartFormula Report

Analysis Info

Analysis Name	D:\Data\dh\20151123\Y37487FMS_WJY-170_41_01_59.d	Acquisition Date	11/24/2015 10:57:23 AM
Method	ESI_POS_150-1000_20151123	Operator	
Sample Name	Y37487FMS_WJY-170	Instrument	solariX
Comment			

Acquisition Parameter

Acquisition Mode	Serial MS	Acquired Scans	2	Calibration Date	Mon Nov 23 03:03:30
Polarity	Positive	No. of Cell Fills	1	Data Acquisition Size	209[illegible]152
Broadband Low Mass	150.5 m/z	No. of Laser Shots	200	Data Processing Size	4194304
Broadband High Mass	1000.0 m/z	Laser Power	30.0 lp	Apodization	Sine-Bell Multiplication
Source Accumulation	0.005 sec	Laser Shot Frequency	0.001 sec		
Ion Accumulation Time	0.100 sec				

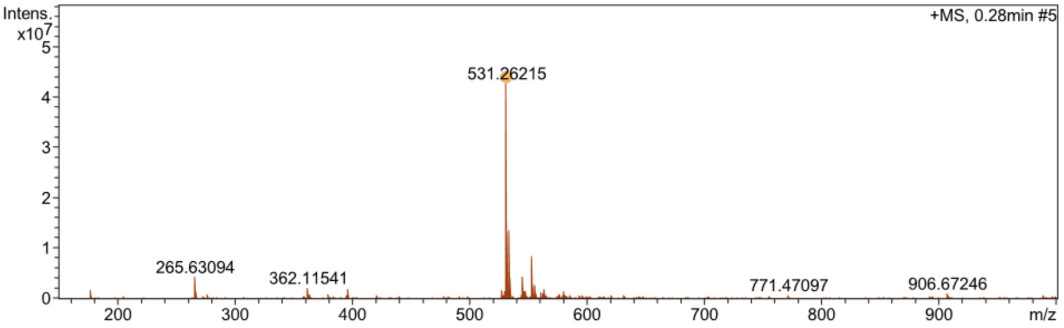

Meas. m/z	#	Ion Formula	Score	m/z	err [ppm]	Mean err [ppm]	mSigma	rdb	e⁻ Conf	N-Rule
531.262148	1	C29H40ClN2O5	100.00	531.262026	-0.2	-0.5	9.6	10.5	even	ok

Figure S42-b. HR-MS of **5** (Positve mode).

Elemental Composition Report — **Page 1**

Single Mass Analysis
Tolerance = 5.0 PPM / DBE: min = -1.5, max = 50.0
Element prediction: Off
Number of isotope peaks used for i-FIT = 3

Monoisotopic Mass, Even Electron Ions
975 formula(e) evaluated with 5 results within limits (up to 50 best isotopic matches for each mass)
Elements Used:
C: 0-35 H: 0-100 N: 0-5 O: 0-10 Cl: 0-2
WJY-170
XMJ_20151028_03 282 (6.059) Cm (280:282)
1: TOF MS ES+
5.82e+004

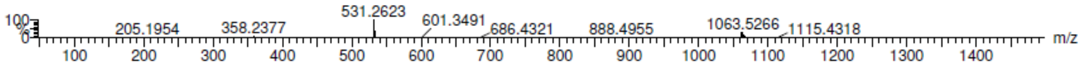

Mass	Calc. Mass	mDa	PPM	DBE	i-FIT	Norm	Conf(%)	Formula
Minimum:				-1.5				
Maximum:		5.0	5.0	50.0				
531.2623	531.2626	-0.3	-0.6	10.5	213.8	0.000	99.99	C29 H40 N2 O5 Cl
	531.2644	-2.1	-4.0	5.5	222.9	9.114	0.01	C28 H45 O5 Cl2
	531.2604	1.9	3.6	1.5	224.2	10.366	0.00	C23 H45 N2 O7 Cl2
	531.2607	1.6	3.0	15.5	226.3	12.483	0.00	C30 H35 N4 O5
	531.2648	-2.5	-4.7	19.5	226.4	12.622	0.00	C35 H35 N2 O3

Table S6. ^{1}H (600 MHz) and ^{13}C (150 MHz) spectroscopic data of **5**.

Position	δ_C, type	δ_H (multi., *J* in Hz)	HMBC (correlations from H to C)
1	197.1, C		
2	61.6, CH	3.88, dd (5.5, 2.1)	1, 3, 4, 27
3	27.5, CH_2	1.82, m	1, 2, 4, 5
		2.05, m	
4	21.1, CH_2	1.18, m	
		1.62, m	
5	39.0, CH_2	2.65, br t (11.2)	3, 4, 7
		3.55, ddd (11.2, 4.9, 3.0)	
6-NH			
7	167.2, C		
8	123.7, CH	5.84, dd (11.5, 1.3)	7, 10
9	141.6, CH	6.06, ddd (11.5, 11.5, 3.4)	7, 10, 11
10	26.4, CH_2	2.53, dd (17.3, 3.0)	8, 9, 11, 22
		3.38, m	
11	45.6, CH	2.14, m	12, 20, 22
12	47.3, CH	2.07, m*	13, 14, 20
13	73.7, CH	4.13, br t (2.6)	11, 14, 20
14	64.7, CH	4.23, br t (2.9)	13, 15, 19
15	47.0, CH	1.77, m	14, 17, 20
16	44.7, CH	1.76, d (3.3)	14, 15, 30
17	32.6, CH	2.21, m	15, 16, 18, 19, 29
18	38.6, CH_2	0.75, m	15, 16, 17, 19, 20, 29
		2.19, d (7.6)	
19	41.1, CH	1.61, m	12, 15, 20
20	42.6, CH	2.07, m*	13, 18, 19
21	35.6, CH_2	1.29, m	11, 12, 20, 22
		2.13, dd (7.6, 4.8)	
22	49.6, CH	2.41, m	11, 21, 23, 24, 25
23	153.0, CH	6.83, dd (15.4, 10.3)	11, 21, 22, 24, 25
24	122.2, CH	7.13, d (15.4)	22, 23, 25, 26
25	173.6, C		
26	100.8, C		
27	175.6, C		
28-NH			
29	17.7, CH3	0.90, d (6.8)	16, 17, 18
30	21.2, CH2	1.35, m	16, 17, 31
31	12.8, CH3	0.94, t (7.38)	16, 30

Measured in 90% $CDCl_3/CD_3OD$. δ values are given in ppm. (* inseperatable)

Figure S43. ^{1}H NMR (600 MHz, MeOD/CDCl$_3$) spectrum of **5**.

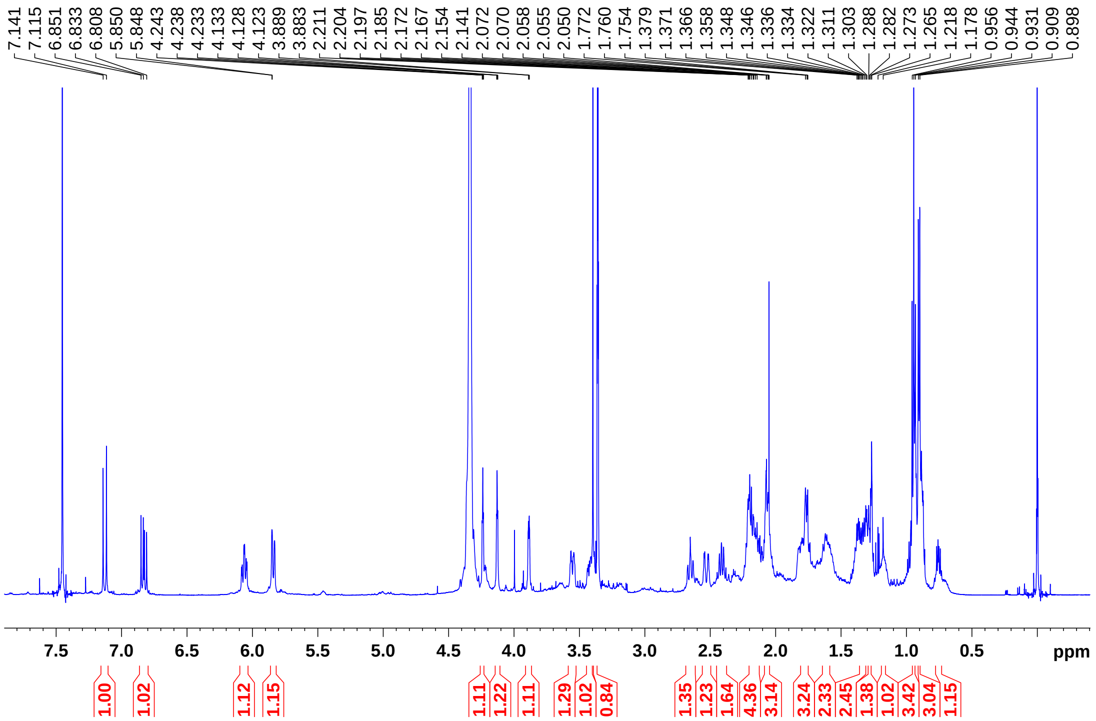

Figure S44. ^{13}C NMR (600 MHz, MeOD/CDCl$_3$) spectrum of **5**.

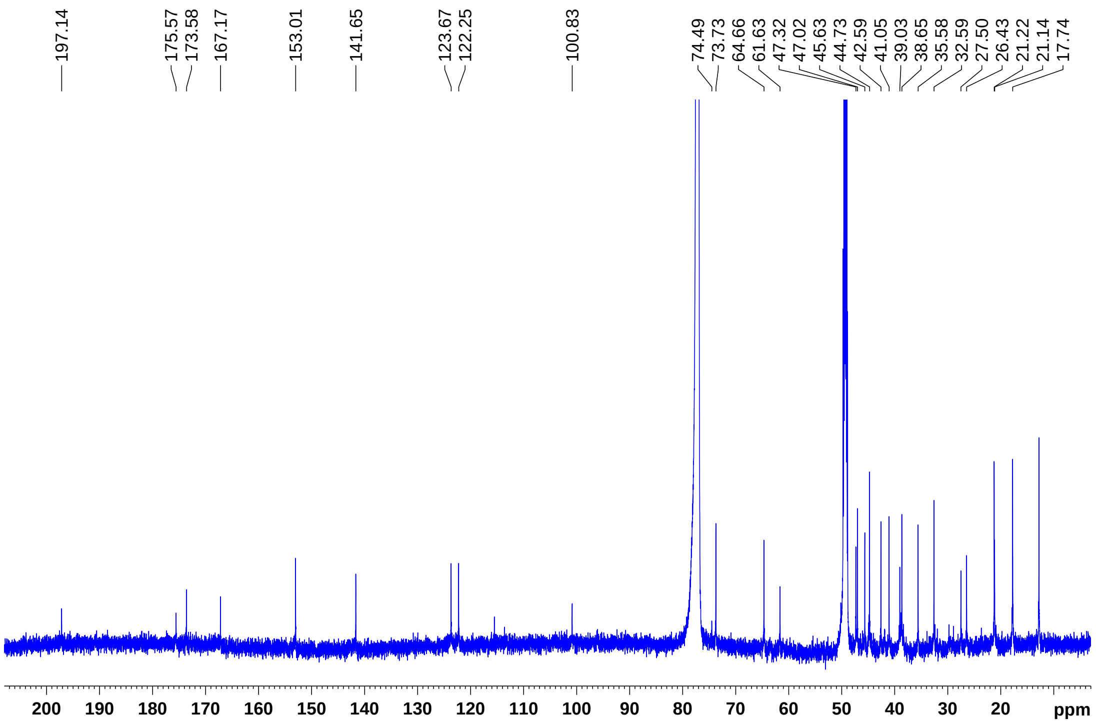

Figure S45. ^{13}C-DEPT NMR (600 MHz, MeOD/$CDCl_3$) spectrum of **5**.

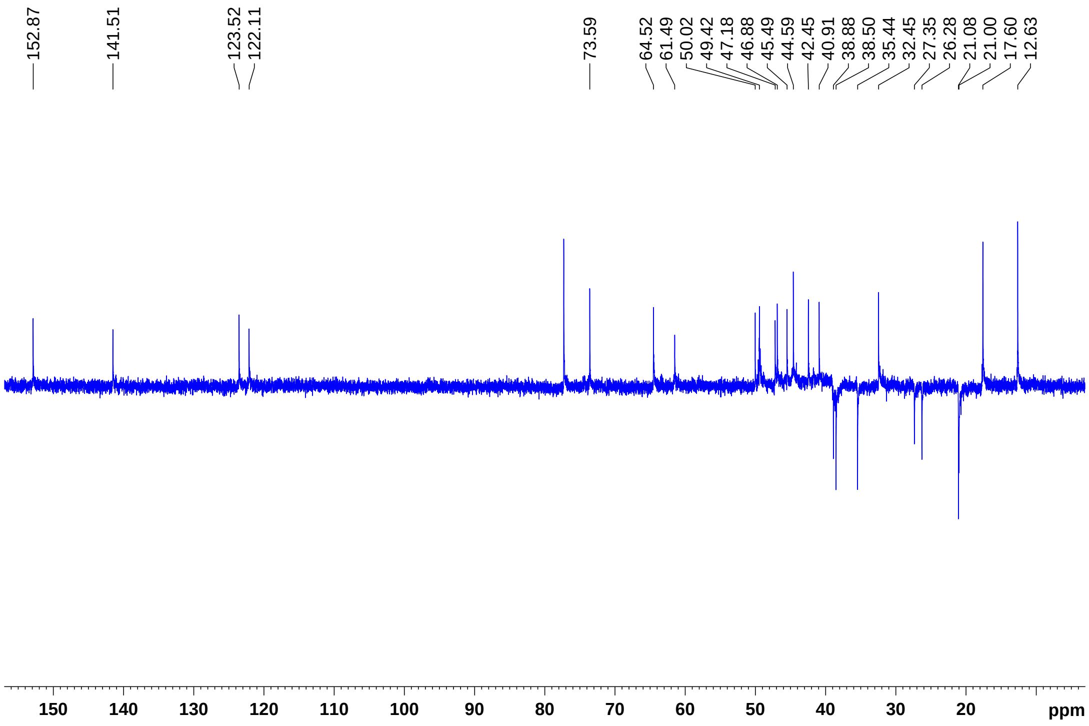

Figure S46. HSQC (600 MHz, MeOD/$CDCl_3$) of **5**.

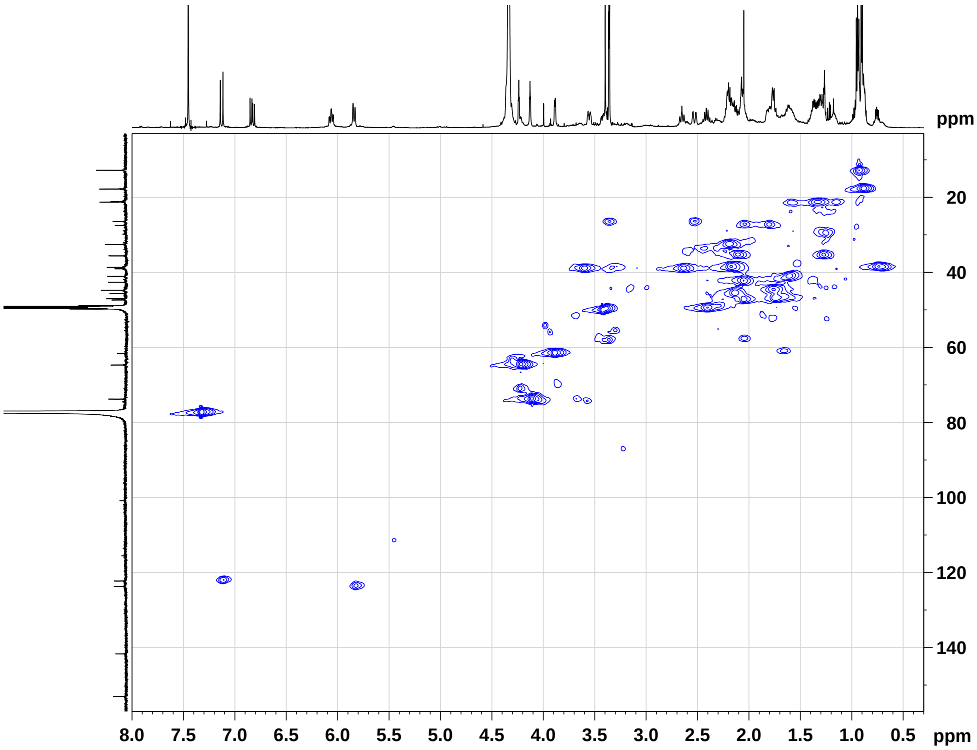

Figure S47. HMBC (600 MHz, MeOD/$CDCl_3$) of **5**.

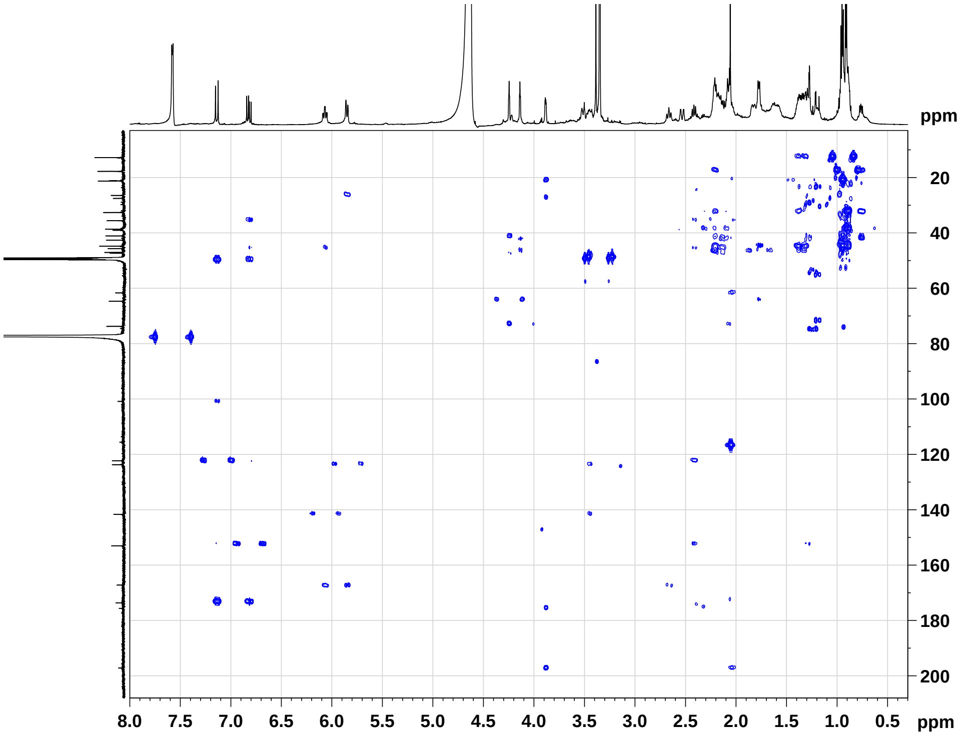

Figure S48. COSY (600 MHz, MeOD/$CDCl_3$) of **5**.

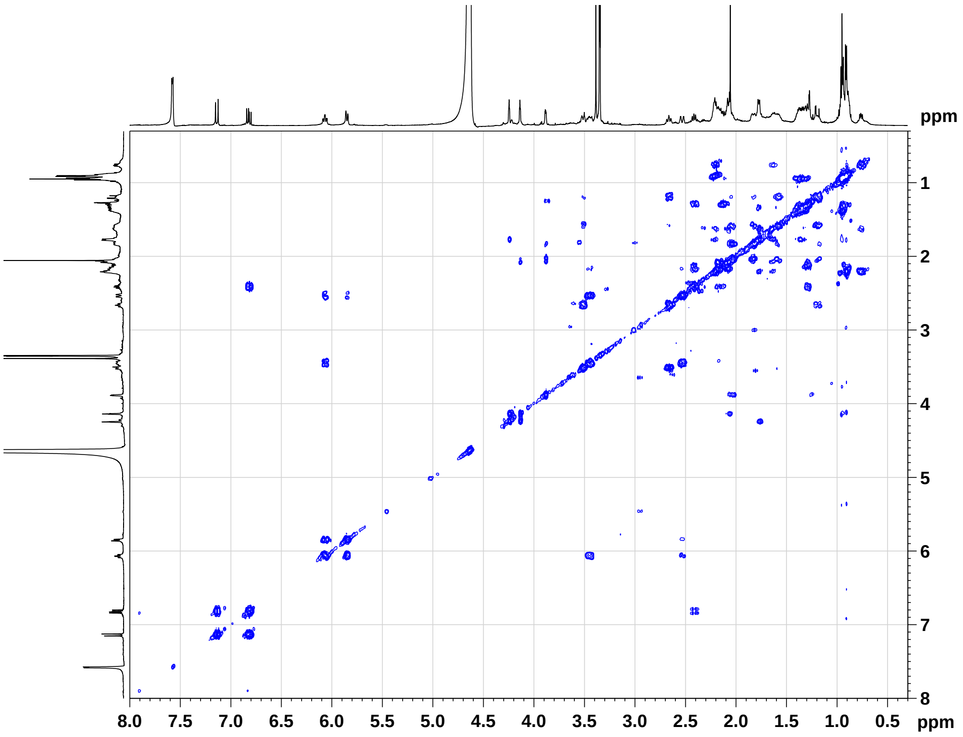

Figure S49. TOCSY (600 MHz, MeOD/$CDCl_3$) of **5**.

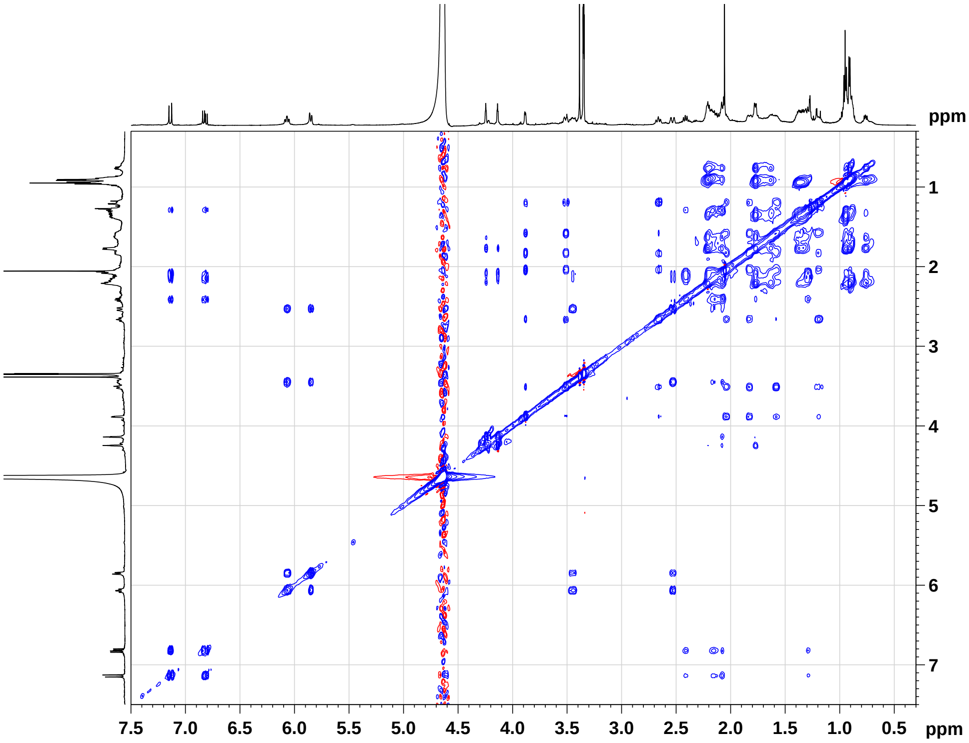

Figure S50-a. NOESY (600 MHz, MeOD/$CDCl_3$) of **5**.

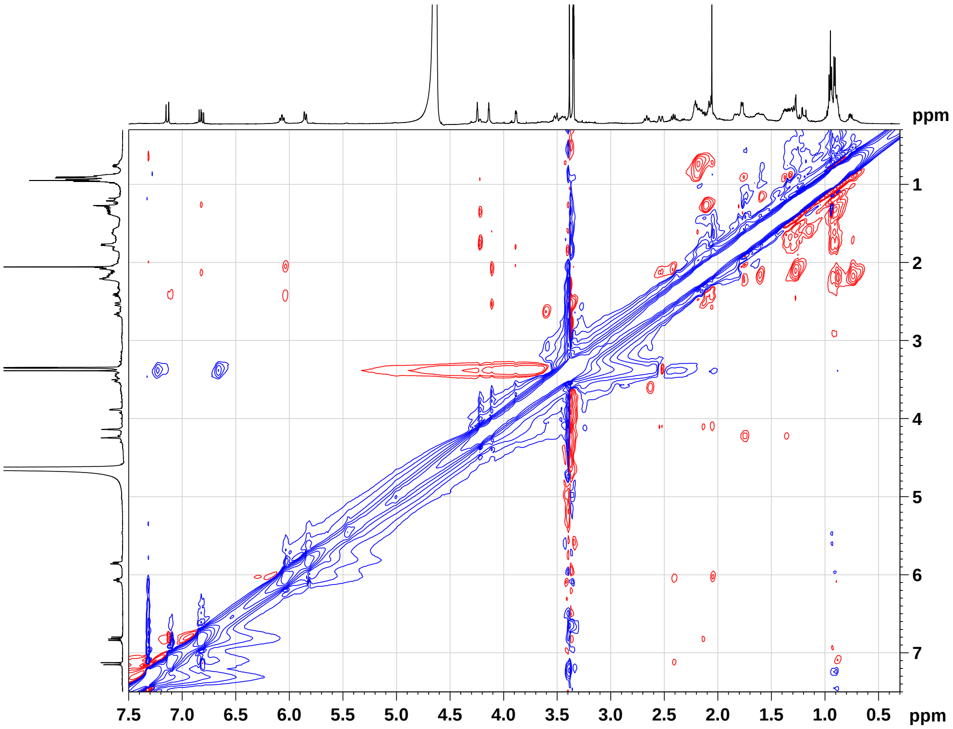

Figure S50-b. ROESY (600 MHz, MeOD/$CDCl_3$) of **5**.

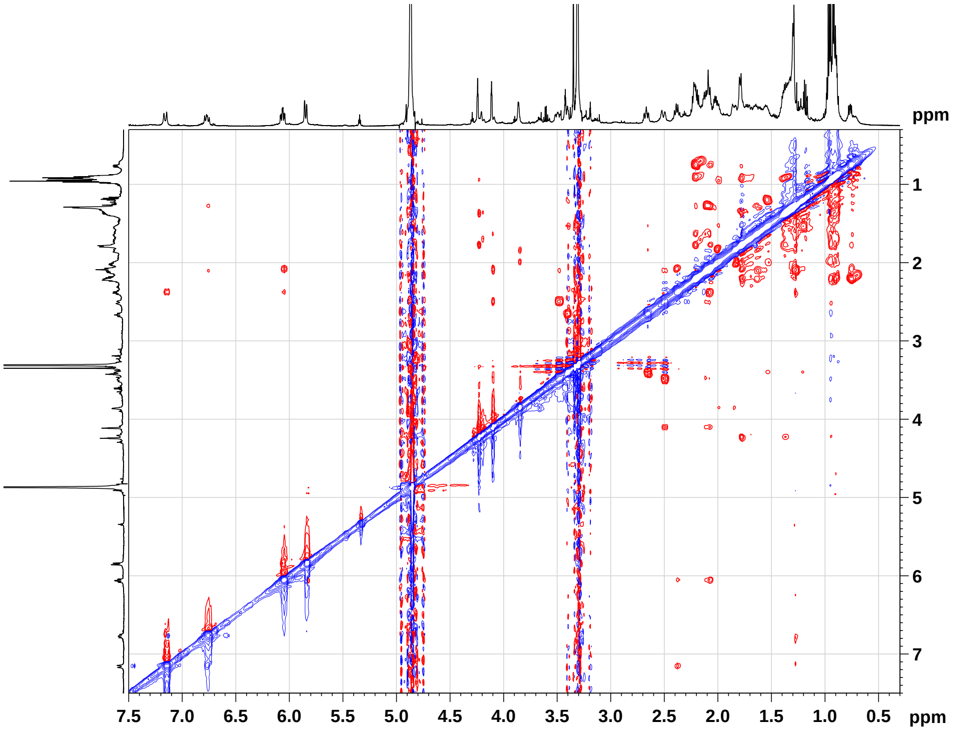

Figure S51. HRESIMS of **6** (Positve mode).

Elemental Composition Report **Page 1**

Single Mass Analysis
Tolerance = 5.0 PPM / DBE: min = -1.5, max = 50.0
Element prediction: Off
Number of isotope peaks used for i-FIT = 3

Monoisotopic Mass, Even Electron Ions
201 formula(e) evaluated with 1 results within limits (up to 50 best isotopic matches for each mass)
Elements Used:
C: 0-40 H: 0-50 N: 0-4 O: 0-10
289-AA-main1
XMJ_20160319_YHL_01 220 (4.717) Cm (219:221) 1: TOF MS ES+
3.73e+004

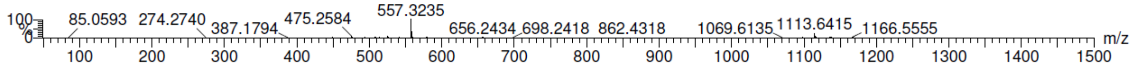

Minimum:				-1.5				
Maximum:		5.0	5.0	50.0				
Mass	Calc. Mass	mDa	PPM	DBE	i-FIT	Norm	Conf(%)	Formula
557.3235	557.3227	0.8	1.4	10.5	228.6	n/a	n/a	C31 H45 N2 O7

Table S7. ^{1}H (600 MHz) and ^{13}C (150 MHz) spectroscopic data of **6**.

Position	δ_C, type	δ_H, multi (*J* in H_Z)	HMBC (correlations from H to C)
1	198.2, C		
2	62.9, CH	3.85, br d (4.0)	1, 3, 4, 27
3	28.3, CH2	1.84, m	1, 2, 4, 5
		1.99, m	
4	22.0, CH2	1.18, m	3, 5
		1.54, m	
5	40.0, CH2	2.66, br t (11.0)	3, 4, 7
		3.40, m	
6-NH			
7	168.8, C		
8	124.7, CH	5.84, d (11.3)	7, 9, 10
9	142.6, CH	6.06, td (11.3, 3.8)	7, 10, 11
10	27.6, CH2	2.45, d (15.7)	8, 9, 11
		3.49, m	
11	45.9, CH	2.01, m	
12	49.6, CH	2.02, m	13, 19, 20
13	68.7, CH	4.08, br	14, 15, 19
14	83.0, CH	3.46, t (2.7)	12, 13, 15, 33
15	44.7, CH	1.69, dd (11.2, 2.6)	16, 19, 30
16	45.9, CH	2.06, m	14, 15, 17, 19, 30, 31
17	35.0, CH	2.18, m	16, 18, 29
18	40.9, CH2	0.67, m	16, 17, 19, 20, 29
		2.03, m	
19	42.7, CH	1.56, m	14, 15, 18, 20, 21
20	42.6, CH	2.00, m	12, 13
21	36.3, CH2	1.27, m	12, 20, 22
		2.08, m	
22	51.2, CH	2.37, m	10, 11, 21, 23, 24
23	153.0, CH	6.75, dd (15.8, 10.5)	11, 21, 22, 24, 25
24	123.7, CH	7.13, d (15.8)	11, 21, 22, 23, 25, 26
25	174.1, C		
26	102.2, C		
27	176.9, C		
28-NH			
29	18.4, CH3	1.00, d (7.1)	16, 17, 18
30	79.1, CH	3.39, m	16, 17, 31, 32
31	18.0, CH3	1.20, d (6.1)	16, 30
32	55.8, CH3	3.29, s	30
33	58.6, CH3	3.42, s	14

Measured in CD_3OD. δ values are given in ppm.

Figure S52. ^{1}H NMR (600 MHz, MeOD) spectrum of **6**.

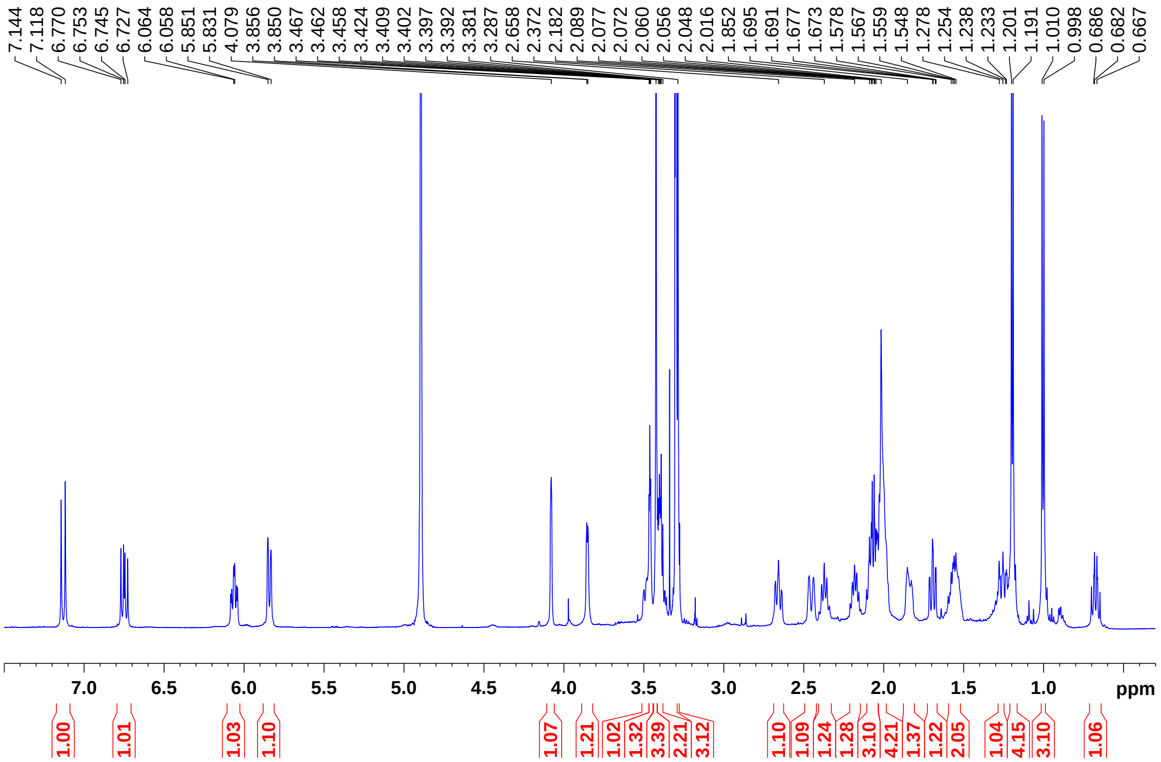

Figure S53. ^{13}C NMR (600 MHz, MeOD) spectrum of **6**.

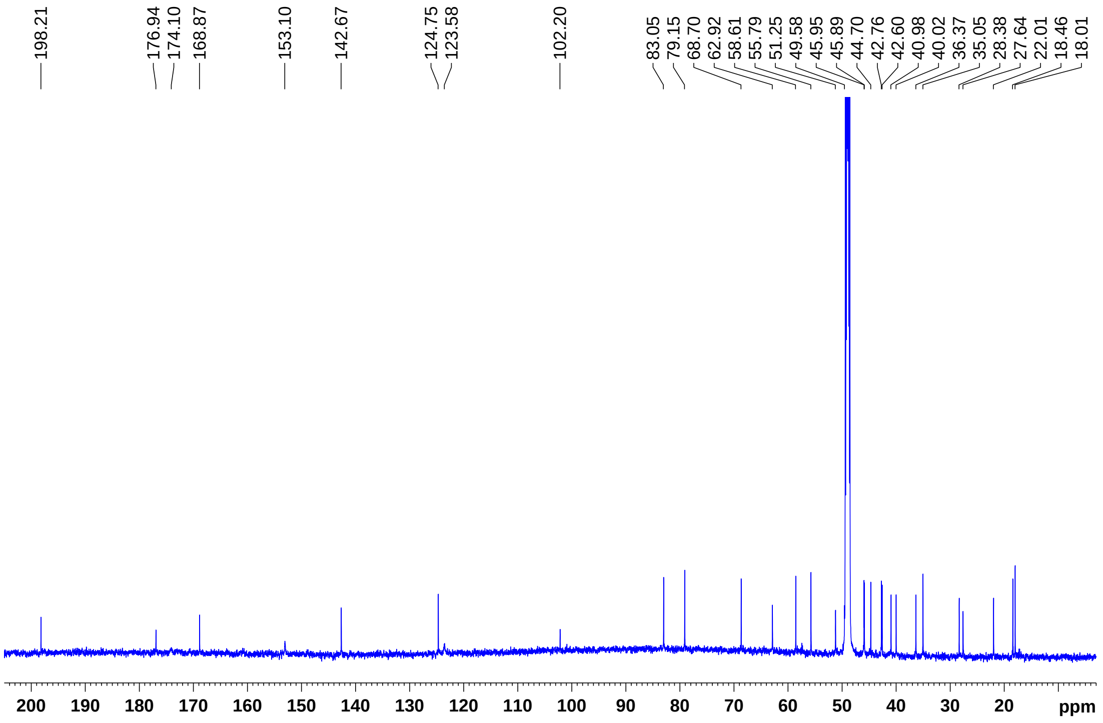

Figure S54. ^{13}C-DEPT NMR (600 MHz, MeOD) spectrum of **6**.

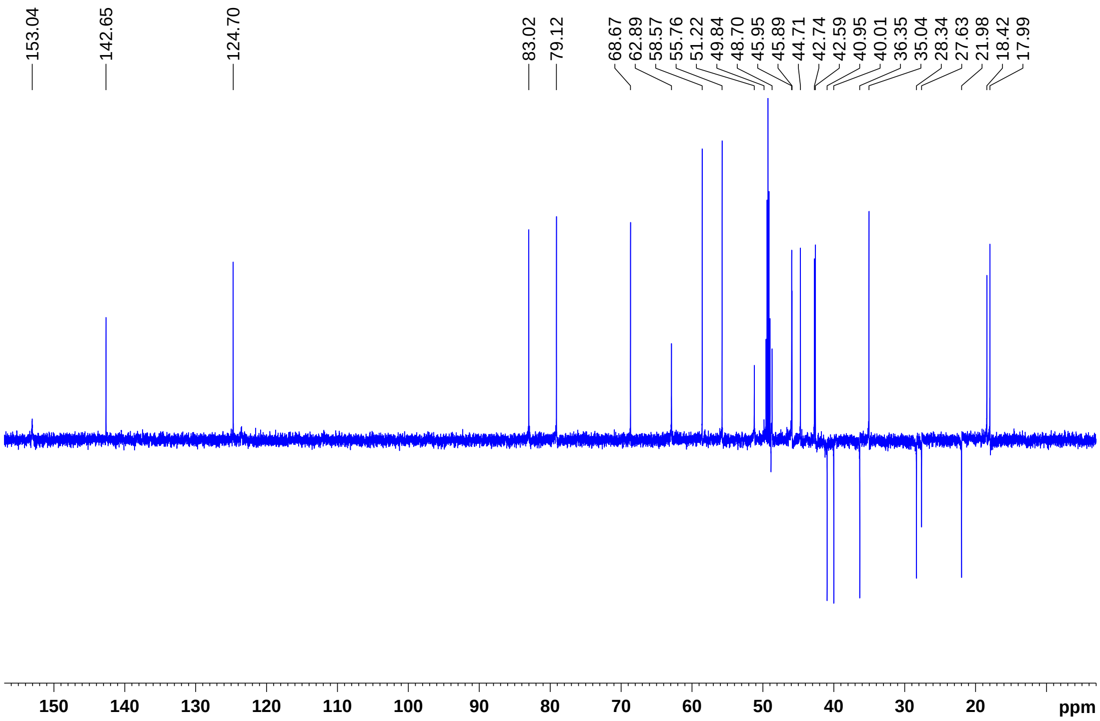

Figure S55. HSQC (600 MHz, MeOD) of **6**.

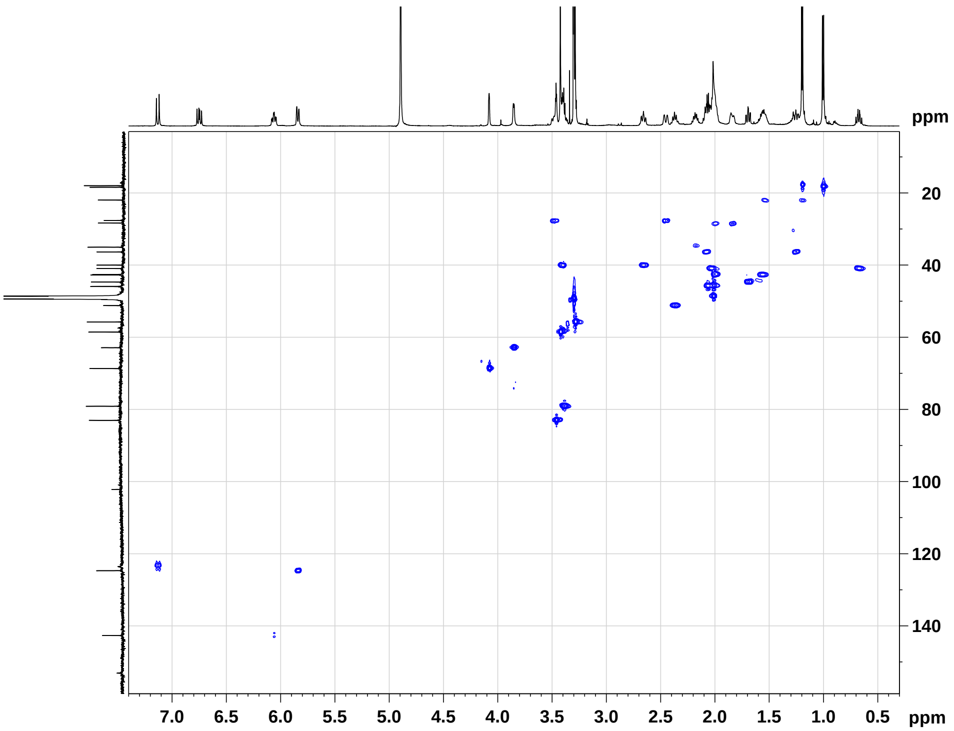

Figure S56. HMBC (600 MHz, MeOD) of **6**.

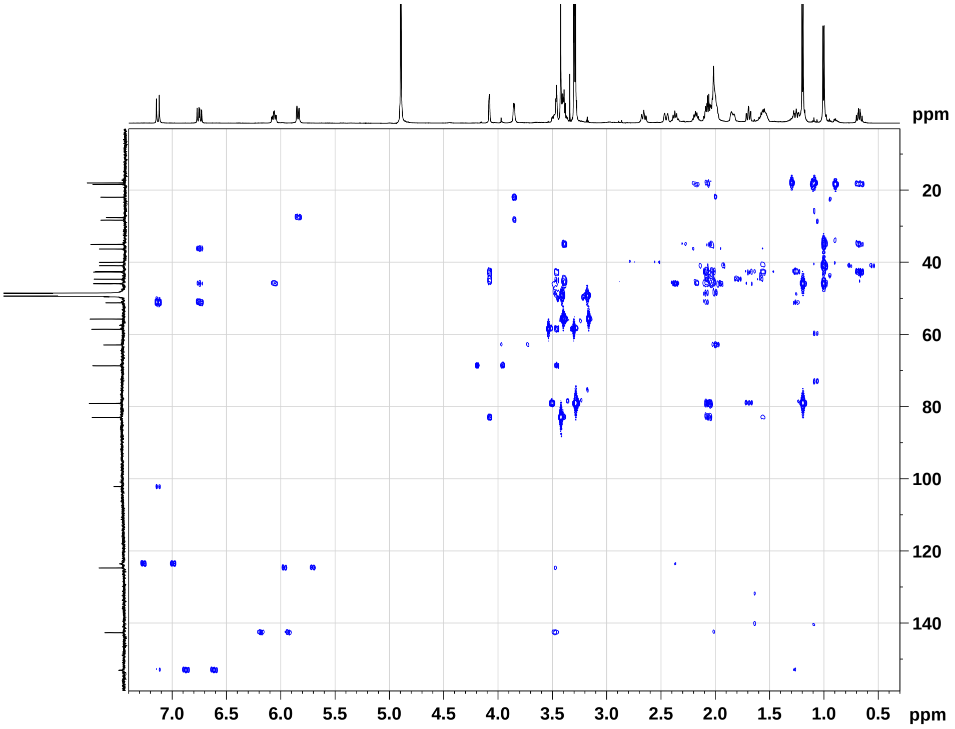

Figure S57. COSY (600 MHz, MeOD) of **6**.

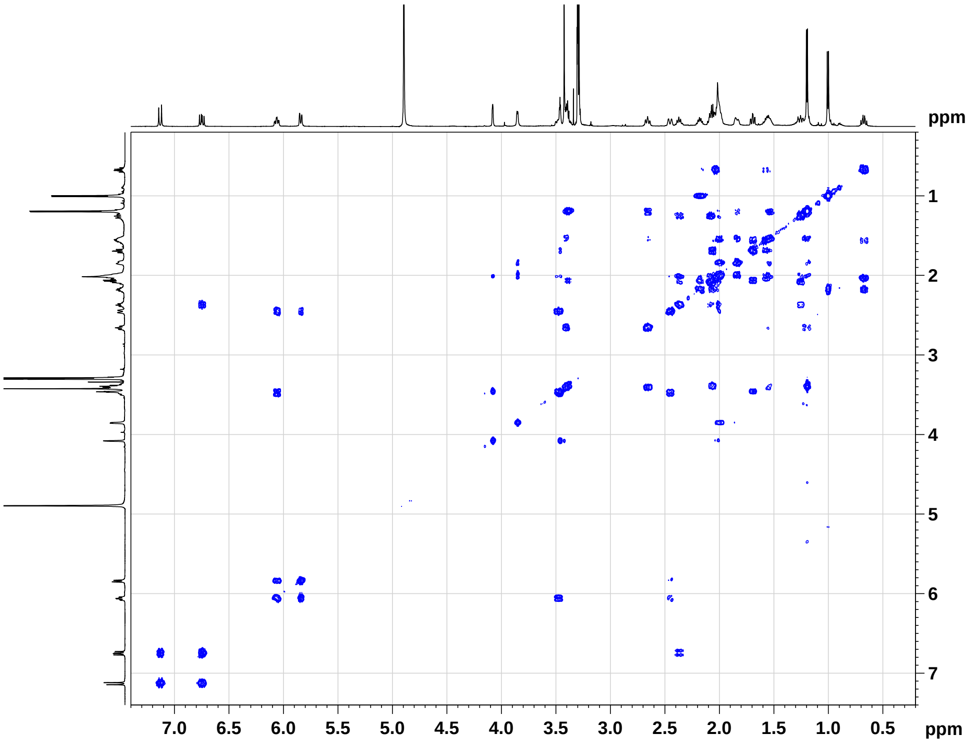

Figure S58-a. NOESY (600 MHz, MeOD) of **6**.

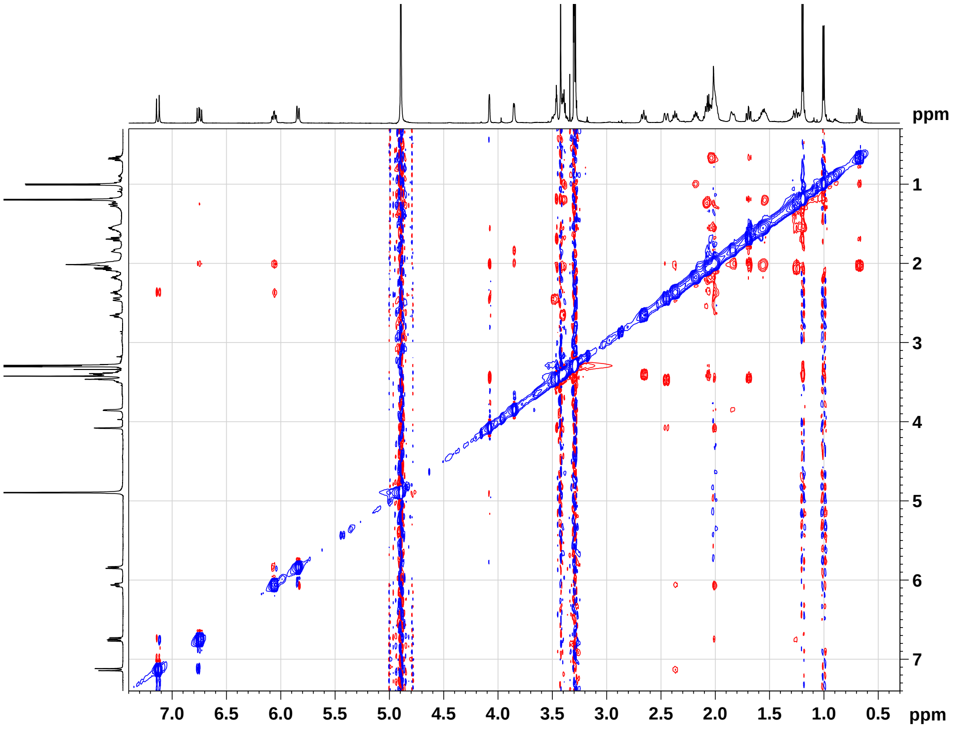

Figure S58-b. ROESY (600 MHz, MeOD) of **6**.

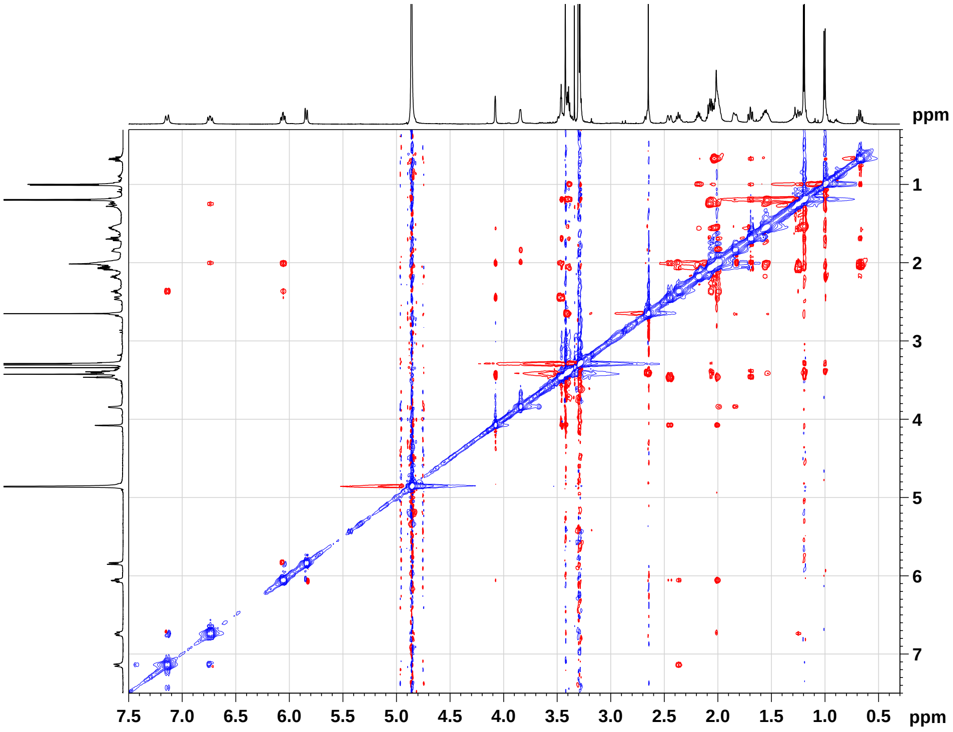

Table S8. ^{1}H (600 MHz) and ^{13}C (150 MHz) spectroscopic data of **7**.

Position	δ_C, type	δ_H, multi (*J* in H_Z)	HMBC (correlations from H to C)
1	198.2, C		
2	62.9, CH	3.85, br d (3.87)	1, 3, 4, 27
3	28.3, CH2	1.84, m	1, 2, 4, 5
		1.99, m	
4	22.0, CH2	1.19, m	3, 5
		1.54, m	
5	40.0, CH2	2.66, br t (10.9)	3, 4, 7
		3.40, m	
6-NH			
7	168.9, C		
8	124.7, CH	5.84, d (10.8)	7, 9, 10
9	142.7, CH	6.06, td (10.8, 3.8)	7, 10, 11
10	27.6, CH2	2.45, br d (15.3)	8, 11, 12
		3.46, m	
11	45.7, CH	2.00, m	9, 12
12	49.6, CH	2.01, m	11, 13, 14, 20
13	68.7, CH	4.08, br t (2.2)	11, 14, 15, 19
14	82.5, CH	3.38, m	13, 19, 33
15	47.6, CH	1.46, dd (11.5, 2.7)	16, 30
16	43.8, CH	1.73, m	15, 17, 29, 30, 31
17	33.8, CH	2.16, m	18, 29
18	40.2, CH2	0.66, m	15, 17, 20, 29
		2.14, m	
19	42.9, CH	1.53, m	14, 15, 18, 20, 21
20	43.0, CH	1.98, m	12, 21
21	36.3, CH2	1.24, m	11, 20, 22, 23
		2.08, m	
22	51.2, CH	2.37, m	10, 11, 21, 23, 24
23	153.2, CH	6.74, dd (15.3, 10.4)	11, 21, 22, 25
24	123.3, CH	7.13, d (15.3)	22, 25, 26
25	173.7, C		
26	102.2, C		
27	177.1, C		
28-NH			
29	18.0, CH3	0.89, d (3.5)	16, 17, 18
30	22.6, CH2	1.37, m	15, 16, 17, 31
31	13.6, CH3	0.95, t (7.6)	16, 30
32			
33	59.0, CH3	3.42, s	14

Figure S59. HRESIMS of **7** (Positve mode).

Elemental Composition Report **Page 1**

Single Mass Analysis
Tolerance = 5.0 PPM / DBE: min = -1.5, max = 50.0
Element prediction: Off
Number of isotope peaks used for i-FIT = 3

Monoisotopic Mass, Even Electron Ions
227 formula(e) evaluated with 2 results within limits (up to 50 best isotopic matches for each mass)
Elements Used:
C: 0-40 H: 0-50 N: 0-4 O: 0-10
315-AA-1
XMJ_20160319_YHL_08 259 (5.555) Cm (258:263) 1: TOF MS ES+ 3.90e+004

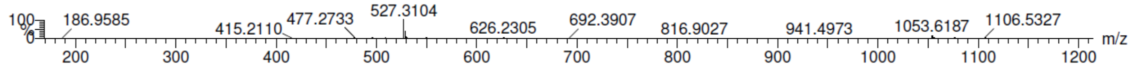

Minimum:				-1.5				
Maximum:		5.0	5.0	50.0				
Mass	Calc. Mass	mDa	PPM	DBE	i-FIT	Norm	Conf(%)	Formula
527.3104	527.3121	-1.7	-3.2	10.5	298.5	0.042	95.86	C30 H43 N2 O6
	527.3081	2.3	4.4	6.5	301.6	3.184	4.14	C25 H43 N4 O8

Figure S60. ^{1}H NMR (600 MHz, MeOD) spectrum of **7**.

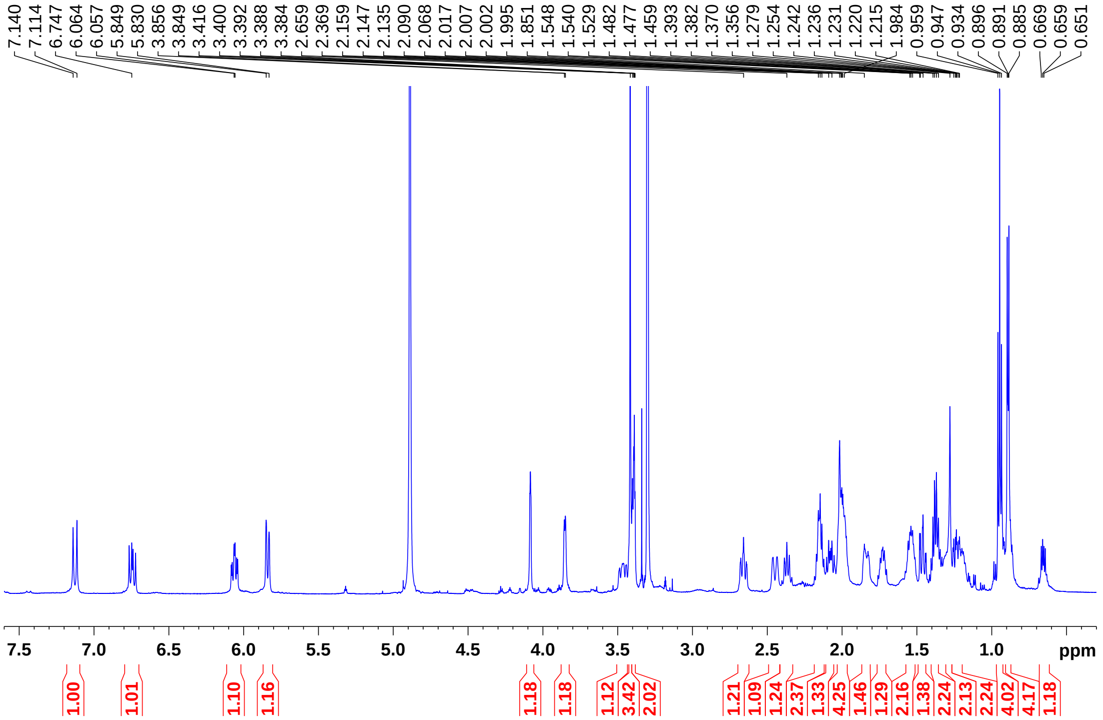

Figure S61. ^{13}C NMR (600 MHz, MeOD) spectrum of **7**.

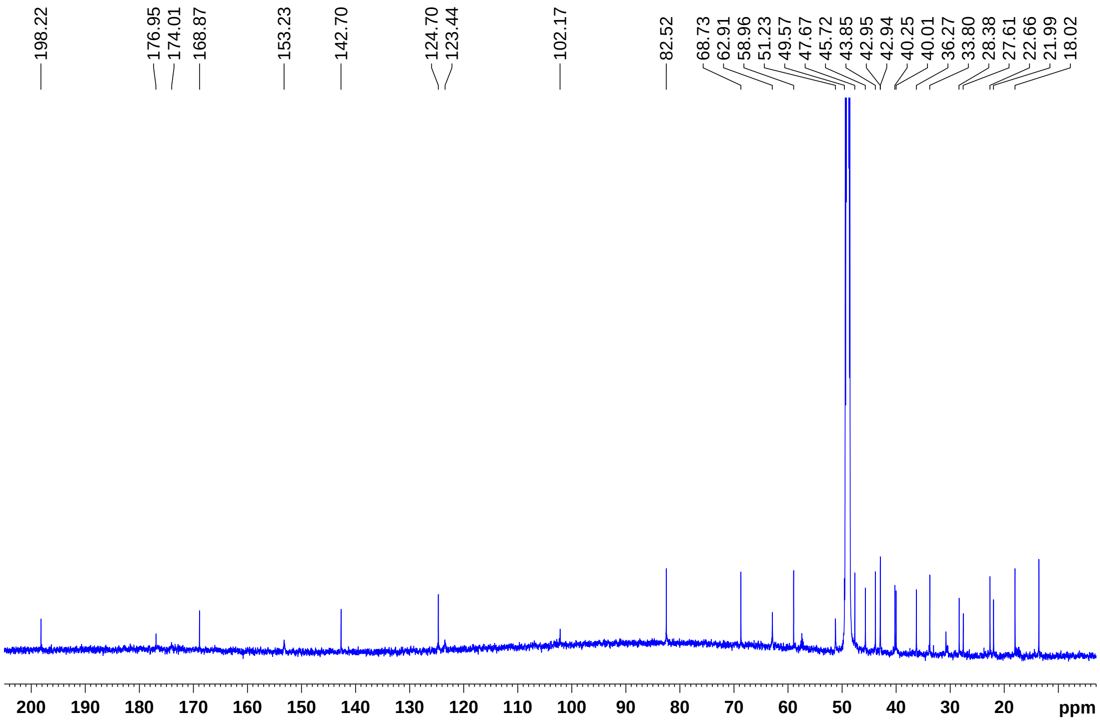

Figure S62. ^{13}C-DEPT NMR (600 MHz, MeOD) spectrum of **7**.

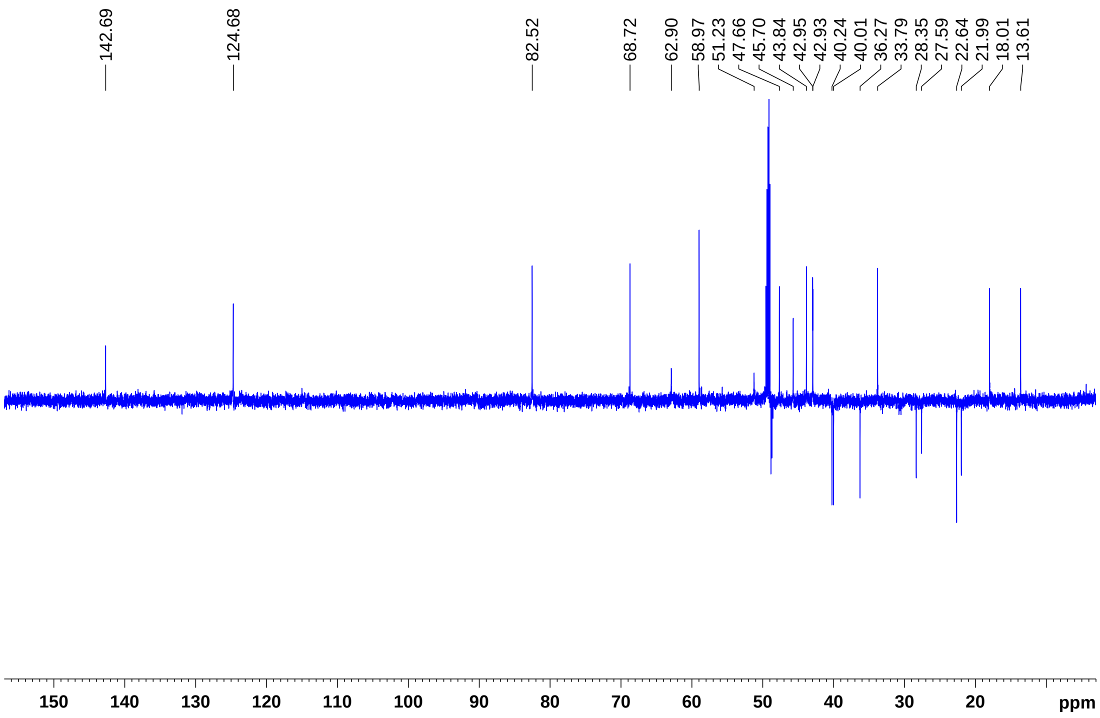

Figure S63. HSQC (600 MHz, MeOD) of **7**.

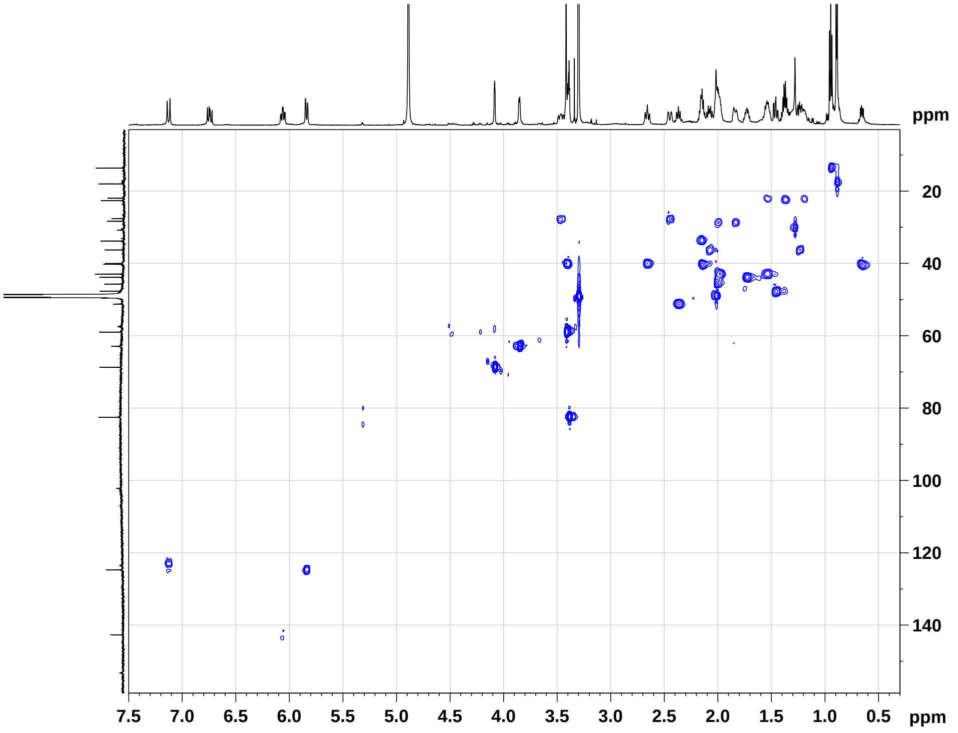

Figure S64. HMBC (600 MHz, MeOD) of **7**.

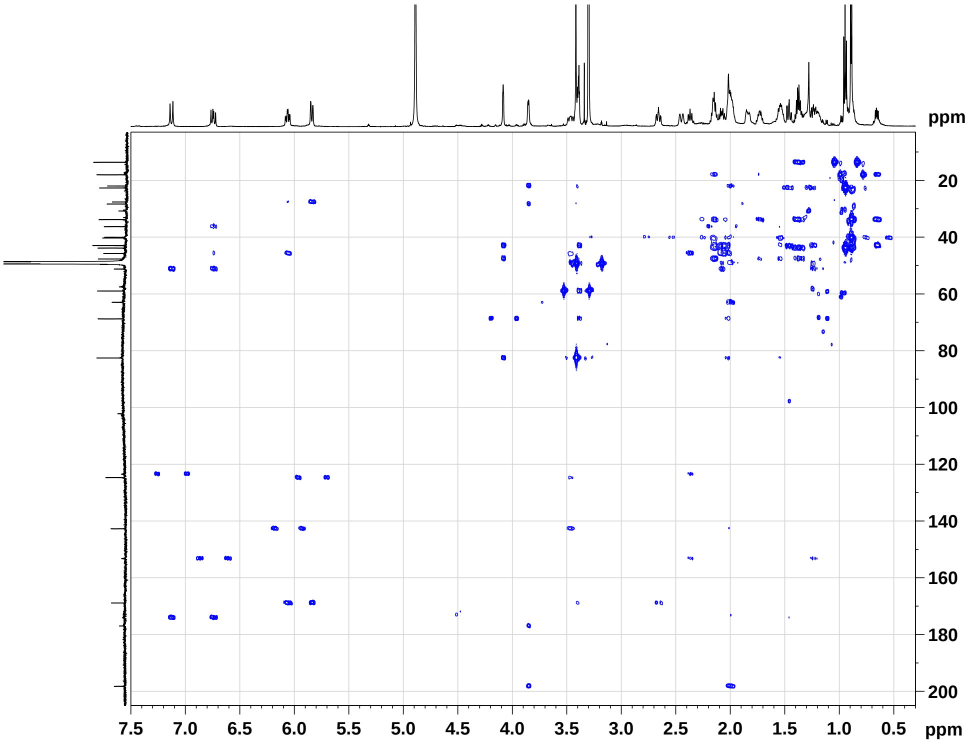

Figure S65. COSY (600 MHz, MeOD) of **7**.

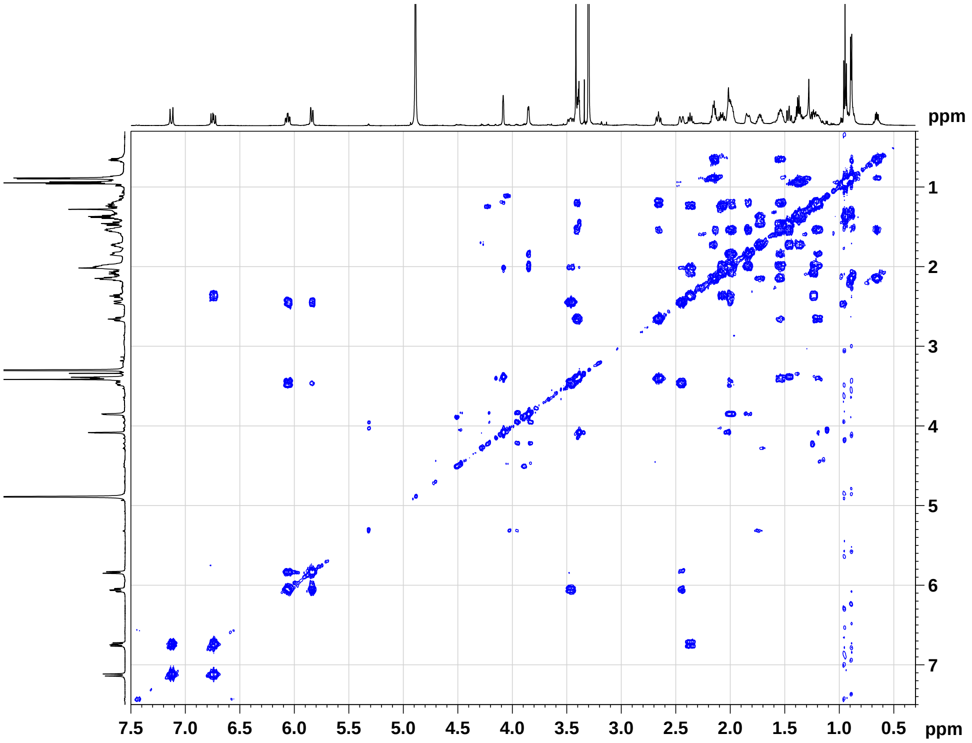

Figure S66-a. NOESY (600 MHz, MeOD) of **7**.

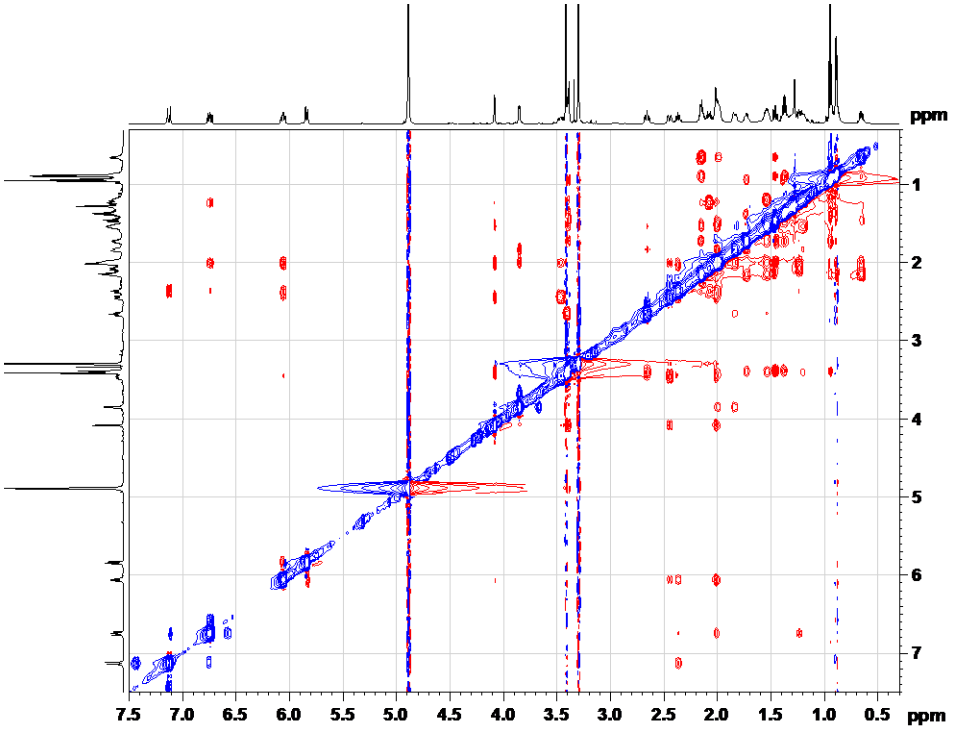

Figure S66-b. ROESY (600 MHz, MeOD) of **7**.

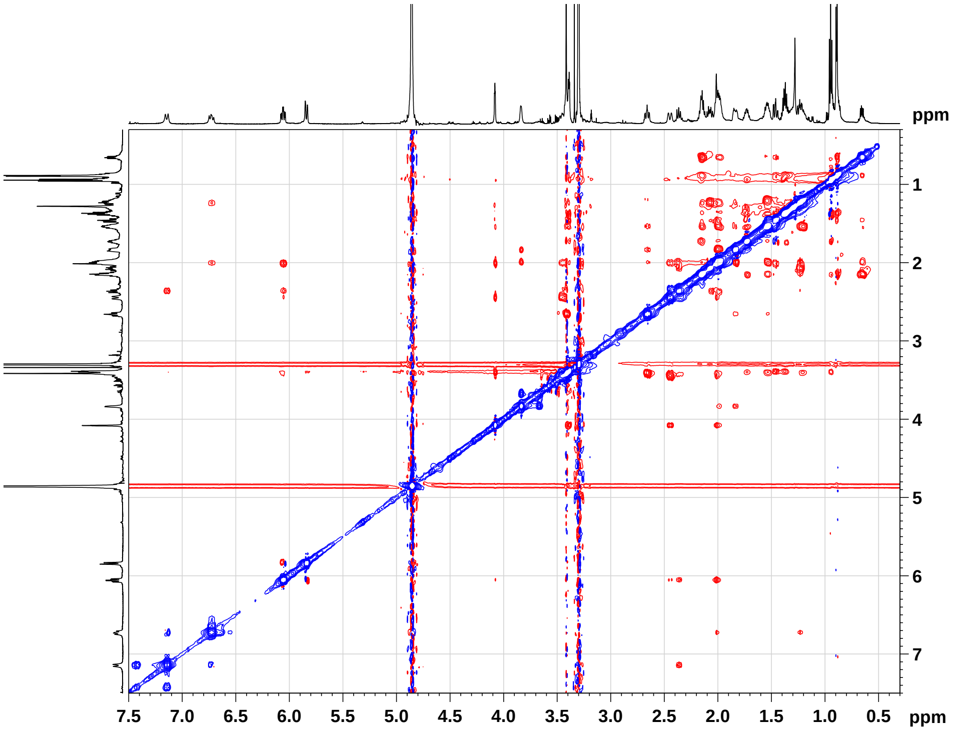

Table S9. ^{1}H (600 MHz) and ^{13}C (150 MHz) spectroscopic data of **3'**.

Position	δ_C, type	δ_H, multi (*J* in Hz)	HMBC (correlations from H to C)
1	198.0, C		
2	62.5, CH	3.78, br d (3.8)	1, 3, 4, 27
3	27.9, CH_2	1.85, m	1, 2
		2.00, m	
4	21.7, CH_2	1.26, m	3, 5
		1.52, m	
5	39.7, CH_2	2.64, m	3, 7
		3.46, br d (12.0)	
6-NH			
7	168.6, C		
8	124.9, CH	5.91, d (11.9)	7, 10
9	141.4, CH	6.05, ddd (11.9, 10.1, 2.8)	7, 11
10	26.8, CH_2	2.42, m	8, 9, 11, 12
		3.63, m	
11	47.1, CH	1.61, m	8, 13
12	41.5, CH	2.28, m	10, 11, 13, 14, 20
13	55.1, CH	2.89, d (3.8)	11, 12, 14, 20
14	59.4, CH	3.26, br s	15, 16
15	48.3, CH	0.93, m	14, 16, 19, 20, 30
16	52.8, CH	1.88, m	14, 15, 17, 29, 30, 31
17	34.8, CH	2.39, m	16, 18, 29
18	40.0, CH_2	0.65, td (11.9, 8.1)	17, 19, 20, 29
		2.04,	
19	48.6, CH	1.12, m	14, 18, 20
20	42.5, CH	1.68, m	11, 12, 18, 19
21	38.0, CH_2	1.10, m	19, 20, 22
		2.02, m	
22	50.0, CH	2.35, m	11, 21, 23, 24
23	149.3, CH	6.68, dd (15.0, 9.8)	11, 21, 22
24	124.7, CH	7.28, m	22
25	176.3, C		
26	102.4, C		
27	177.5, C		
28-NH			
29	18.3, CH_3	1.08, d (7.74)	16, 17, 18
30	68.9, CH	3.87, m	16, 17
31	23.5, CH_3	1.36, d (6.3)	16, 30

Measured in mixture solvent 90% MeOD/ C_6D_6.

Figure S67. HRESIMS of **3'** (Positve mode).

Single Mass Analysis
Tolerance = 10.0 PPM / DBE: min = -1.5, max = 50.0
Element prediction: Off
Number of isotope peaks used for i-FIT = 3

Monoisotopic Mass, Even Electron Ions
188 formula(e) evaluated with 2 results within limits (up to 50 closest results for each mass)
Elements Used:
C: 25-35 H: 0-45 N: 0-5 O: 0-10
511-2
XMJ_20160613_YHL_02 197 (3.841) Cm (196:198) 1: TOF MS ES+ 1.59e+004

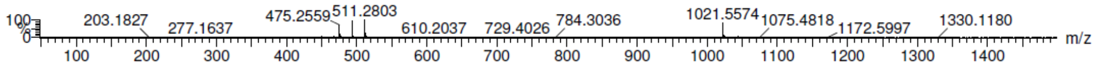

Minimum:				-1.5				
Maximum:		5.0	10.0	50.0				
Mass	Calc. Mass	mDa	PPM	DBE	i-FIT	Norm	Conf(%)	Formula
511.2803	511.2808	-0.5	-1.0	11.5	203.4	0.094	91.01	C29 H39 N2 O6
	511.2848	-4.5	-8.8	15.5	205.7	2.409	8.99	C34 H39 O4

Figure S68. ^{1}H NMR (600 MHz, 90% MeOD/C_6D_6) spectrum of **3'**.

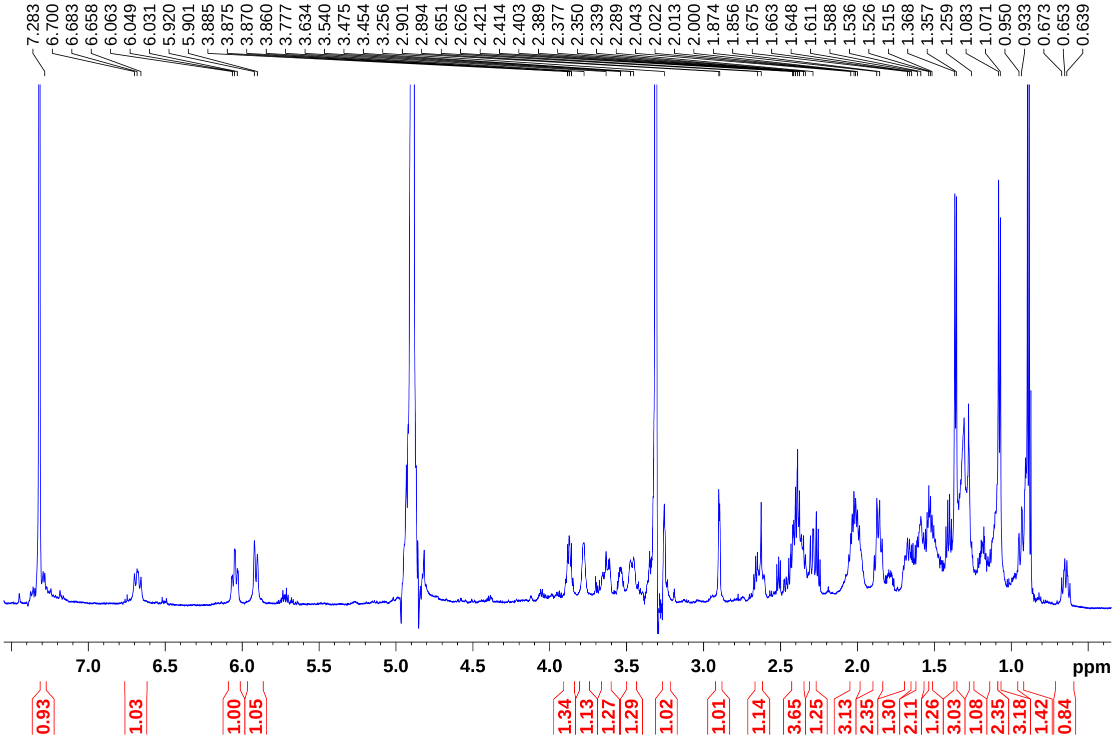

Figure S69. ^{13}C NMR (600 MHz, 90% MeOD/C_6D_6) spectrum of **3'**.

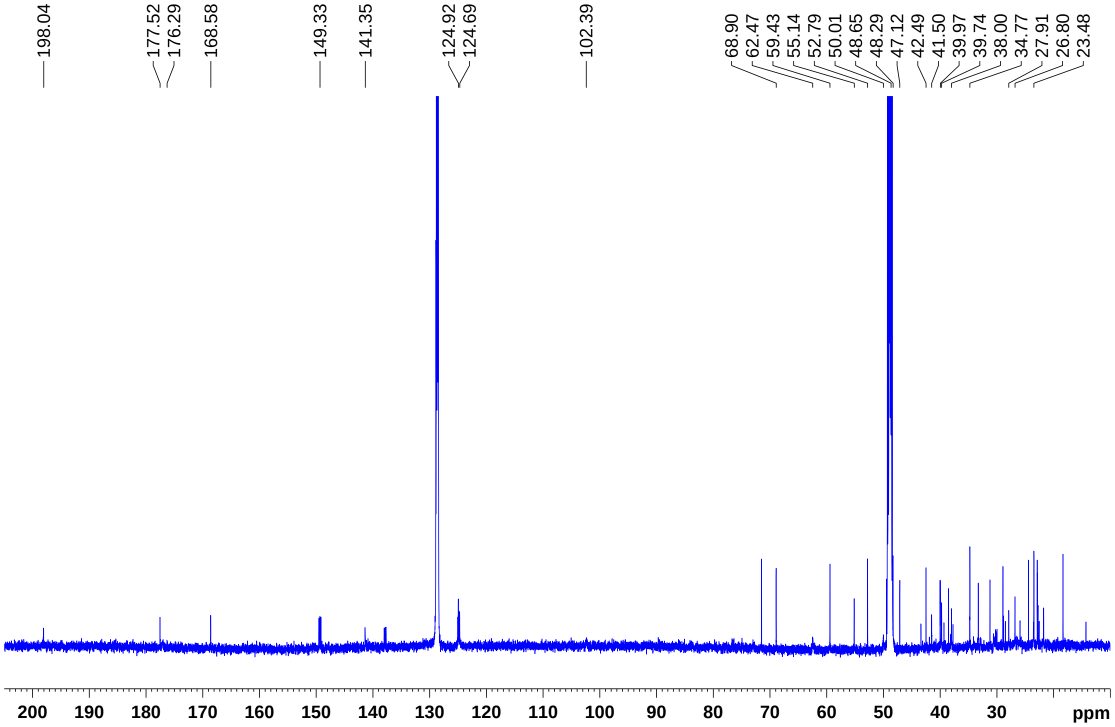

Figure S70. ^{13}C-DEPT NMR (600 MHz, 90% MeOD/C_6D_6) spectrum of **3'**.

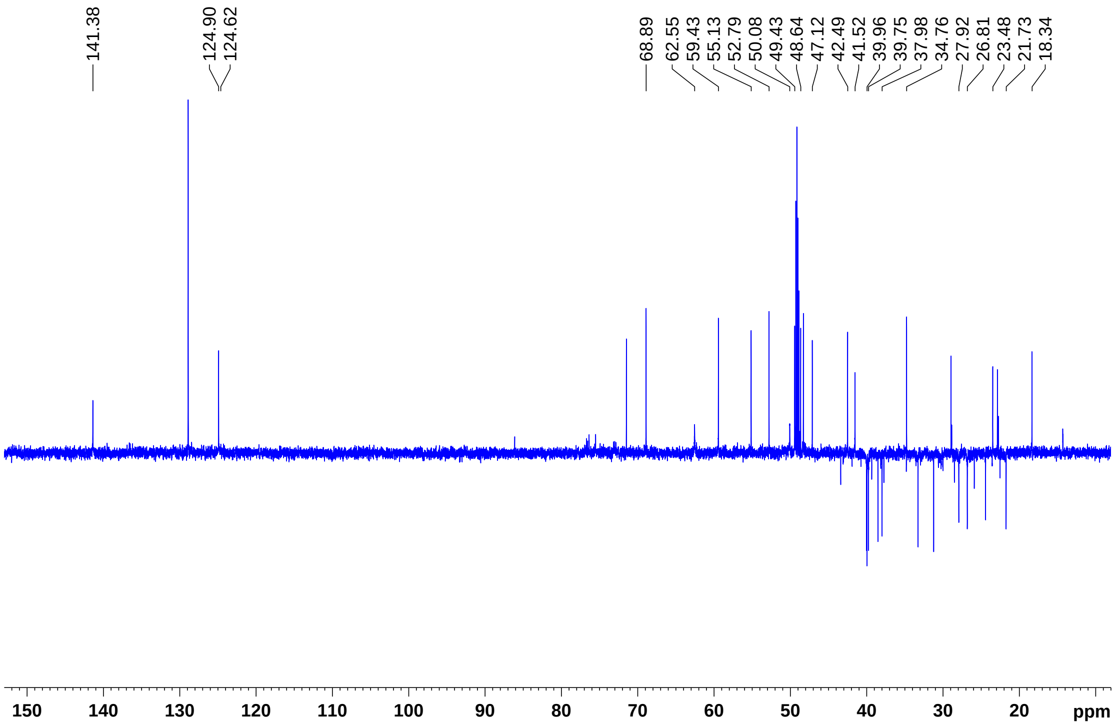

Figure S71. HSQC (600 MHz, 90% MeOD/C_6D_6) of **3'**.

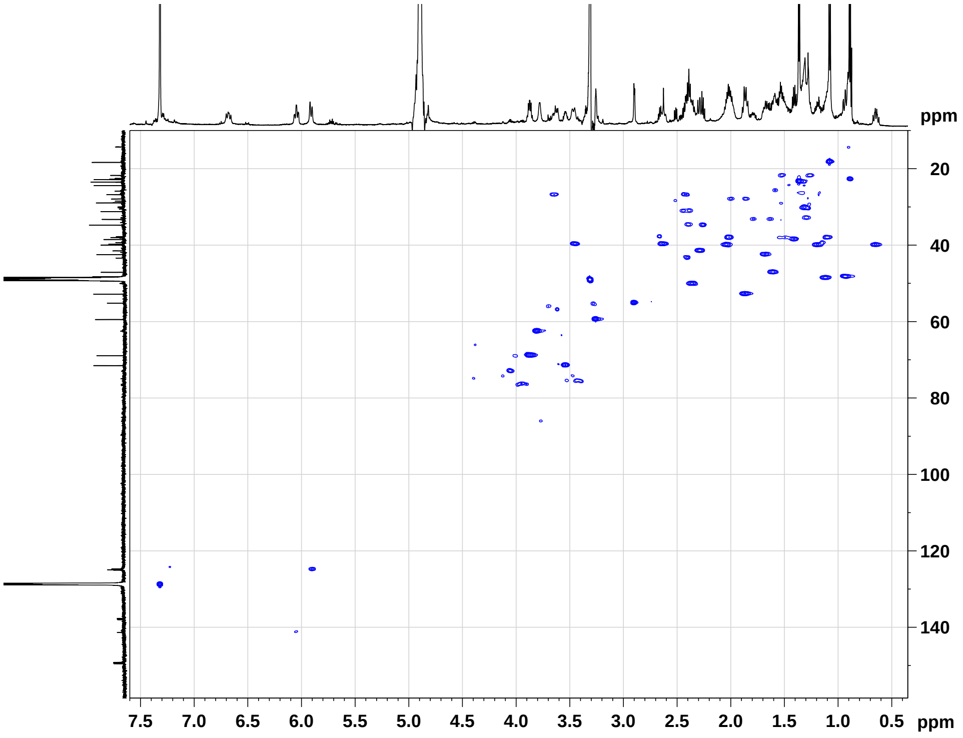

Figure S72. HMBC (600 MHz, 90% MeOD/C_6D_6) of **3'**.

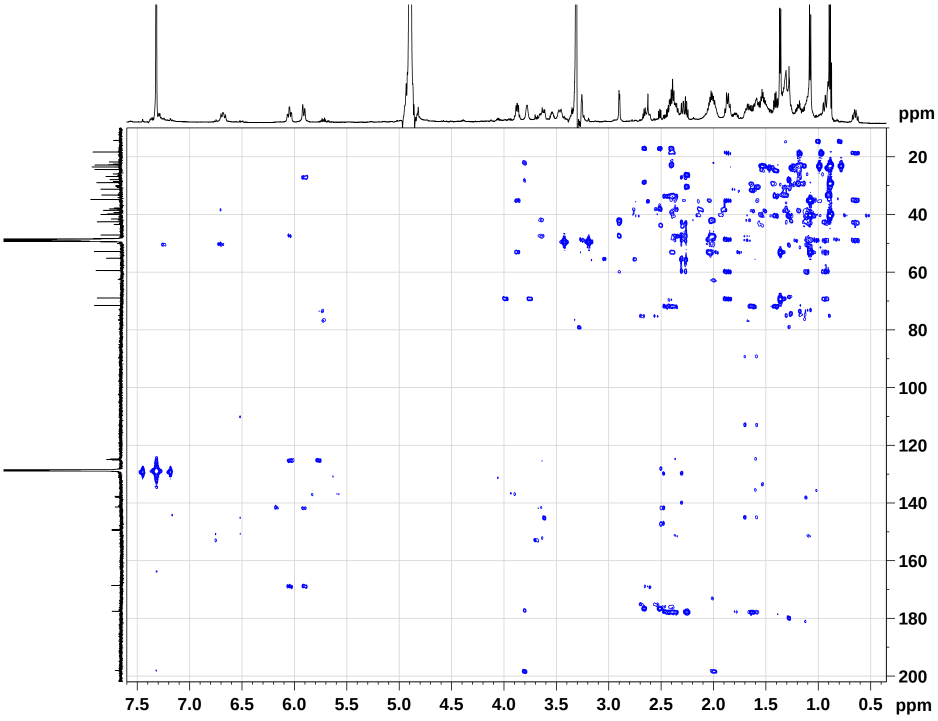

Figure S73. COSY (600 MHz, 90% MeOD/C_6D_6) of **3'**.

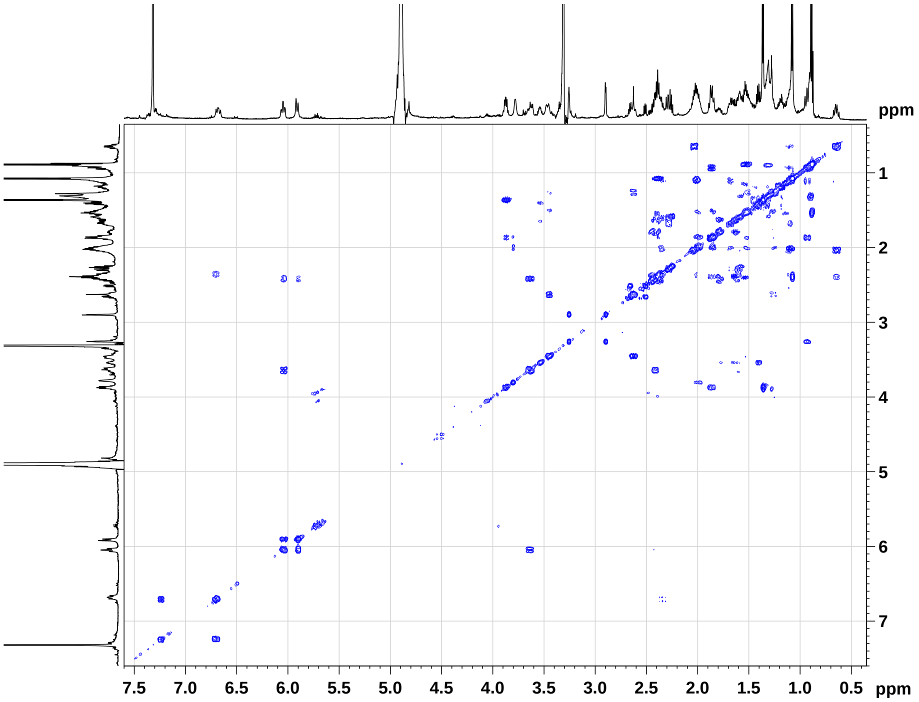

Figure S74. NOESY (600 MHz, 90% MeOD/C_6D_6) of **3'**.

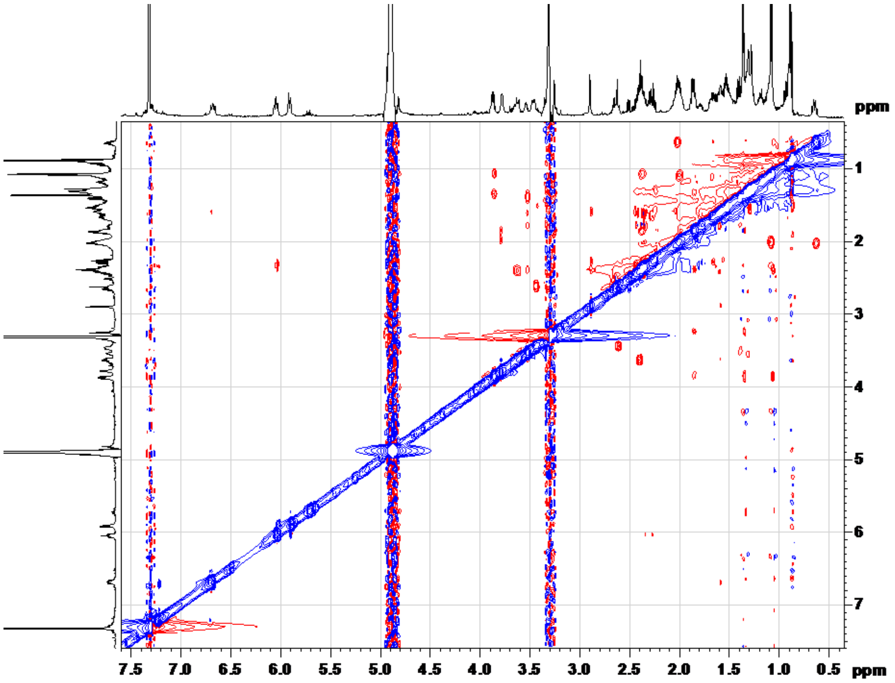

Figure S75. Kinetic analysis with Surface Plasmon Resonance (SPR) for A) ikarugamycin (**1**) and B) capsimycin B (**3**) binding to the IkaD.

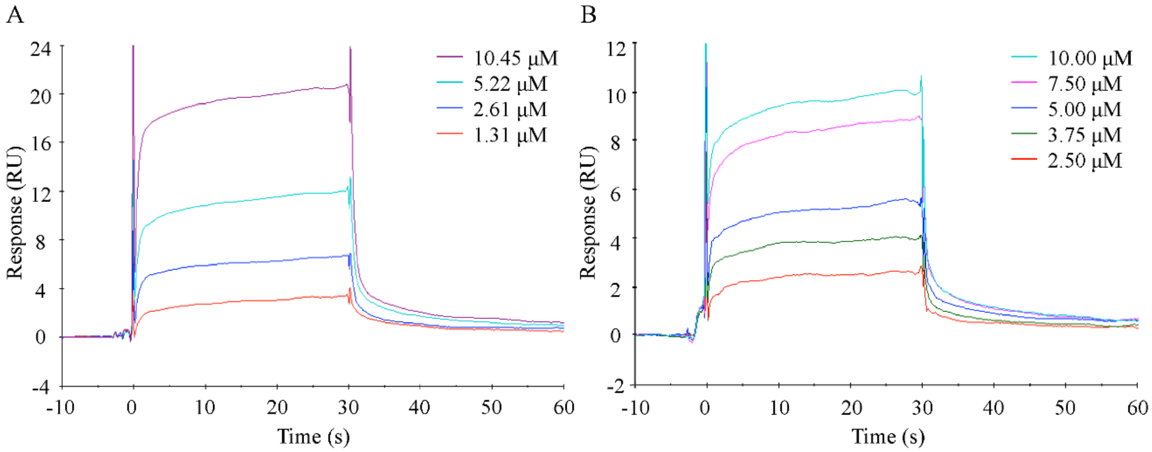

Table S10. Binding affinities and kinetic parameters for the interactions between ikarugamycin (**1**) and capsimycin B (**3**) with IkaD as measured with SPR.

	k_a (1/Ms)	k_d (1/s)	K_D (M)
ikarugamycin (**1**)	2.37×10^4	0.1305	5.50×10^{-6}
capsimycin B (**3**)	1.07×10^4	0.0713	6.65×10^{-6}

k_a, association rate constant; k_d, dissociation rate constant; K_D, equilibrium dissociation constant.

Surface Plasmon Resonance (SPR) assay

All binding kinetic experiments were performed at 25 °C using Biacore T200 instrument equipped with a CM5 sensor chip (GE Healthcare, USA). The surface of the CM5 chip was pre-activated with a 1:1 mixture of 0.05 M

N-hydroxysuccinimide (NHS) and 0.2 M *N*-ethyl-*N'*-(3-dimethylaminopropyl) carbodiimide (EDC). Then fresh IkaD, diluted to 70 μg/mL in 10 mM sodium acetate (pH 4.0), was immobilized on the CM5 chip via amino coupling, following blocking by 1 M ethanolamine HCl (pH 8.5). An empty chip surface was submitted as a negative control to monitor nonspecific binding between compounds with the surface. Ikarugamycin (**1**) and capsimycin B (**3**) were sequentially diluted with running buffer to different concentrations (ikarugamycin: 0-10.45 μM; capsimycin B: 0-10 μM), and injected with HBS-EP+ running buffer over the chip surfaces at a constant flow rate of 30 μL/min. Between experiments, surfaces were regenerated with 50 mM glycine (pH 9.5). Nonspecific sensorgrams were subtracted from experimental sensorgrams to obtain curves representing specific binding. Kinetics and affinity parameters were evaluated in global fitting based on a 1:1 binding model by using Biacore evaluation software (version 3.0).

Figure S76. The reactions of capsimycin B (**3**) with NaX (X = Cl, Br, I). Targeted products were detected under the following conditions. A) Reaction of compound **3** in MeOH/H_2O; UPLC-total ion chromatography MS profiles of the products produced under the condition of B) 1 h after the mixing with TFA at room temperature; C) 1 h after the mixing with TFA at 45 ºC; D) MS spectra of (a) capsimycin B + HCl, (b) capsimycin B + HBr, and (c) capsimycin B + HI.

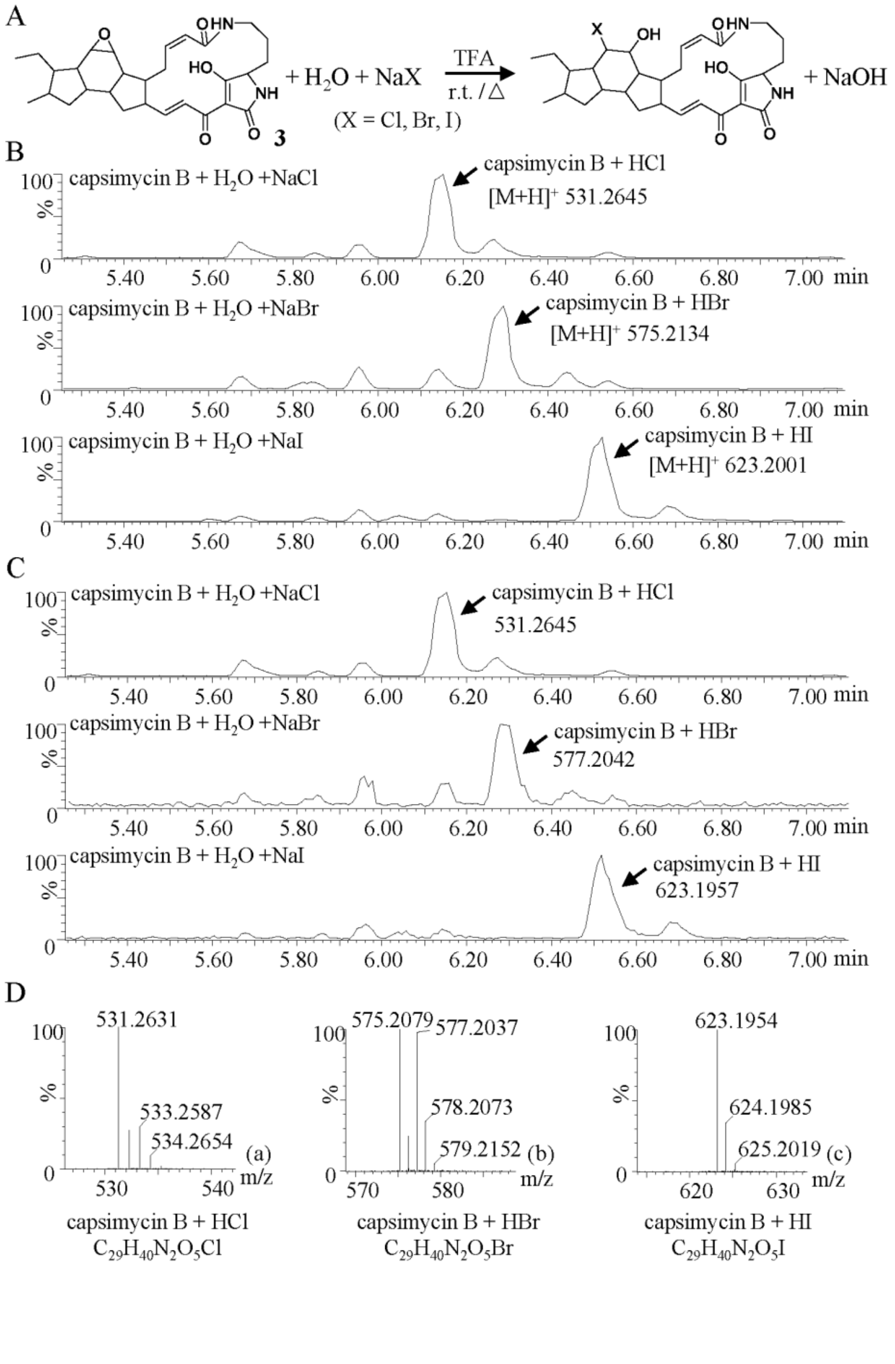

Figure S77. The reactions of capsimycin B (**3**) with NaX (X = Cl, Br, I). No products were detected under the following conditions. A) Reaction of compound **3** in MeOH/H_2O; UPLC-total ion chromatography MS profiles of the products produced under the condition of B) immediately after the mixing at room temperature; C) 1 h after the mixing at room temperature; D) 1 h after the mixing at 45 ºC.

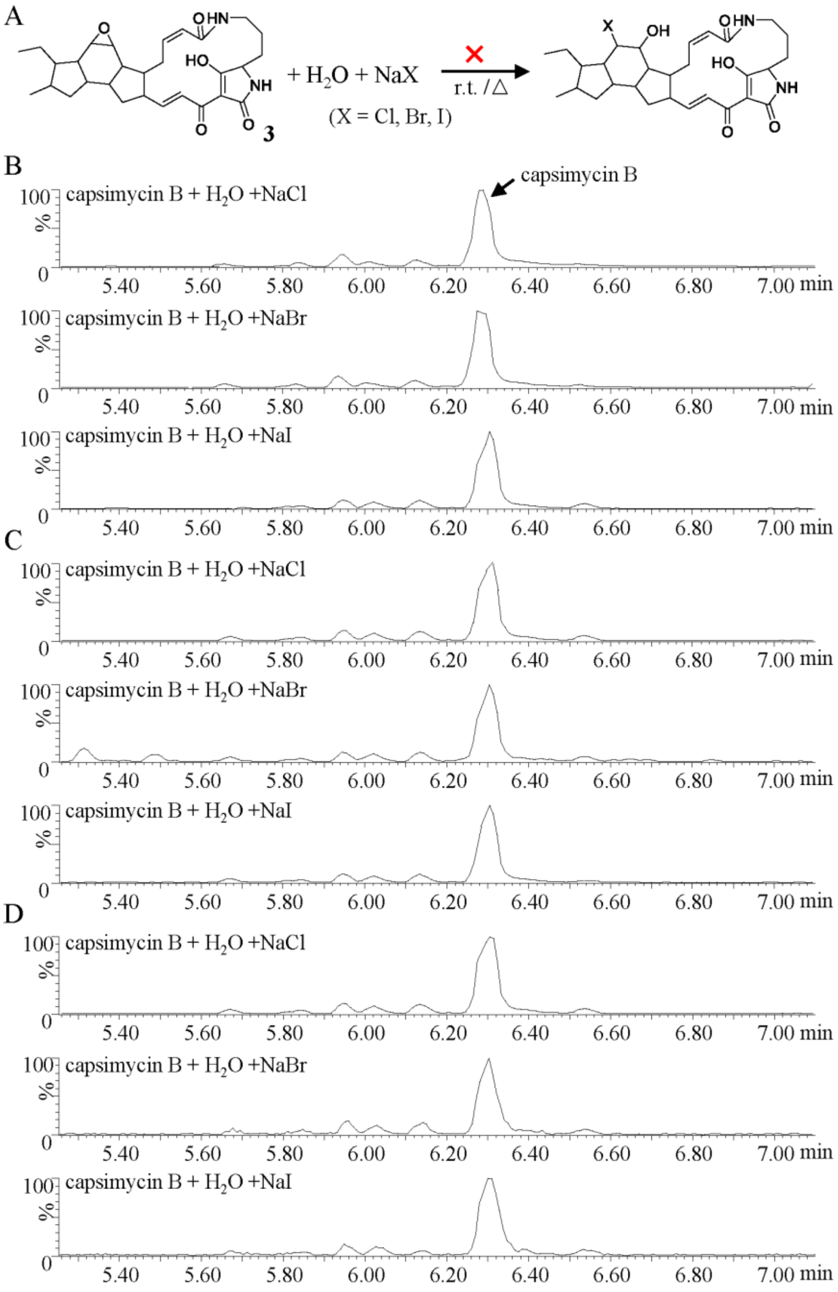

Figure S78. The reactions of capsimycin B (**3**) with H_2O. Targeted product (**4**) was detected under the following conditions. A) Reaction of compound **3** in MeOH/H_2O can be accelerated by the TFA; B) UPLC-total ion chromatography MS profiles of the products produced under the condition of a) immediately after the mixing at room temperature; b) 1 h after the mixing at 45 °C; c) 1 h after the mixing with TFA at room temperature; (d) 1 h after the mixing with TFA at 45 °C.

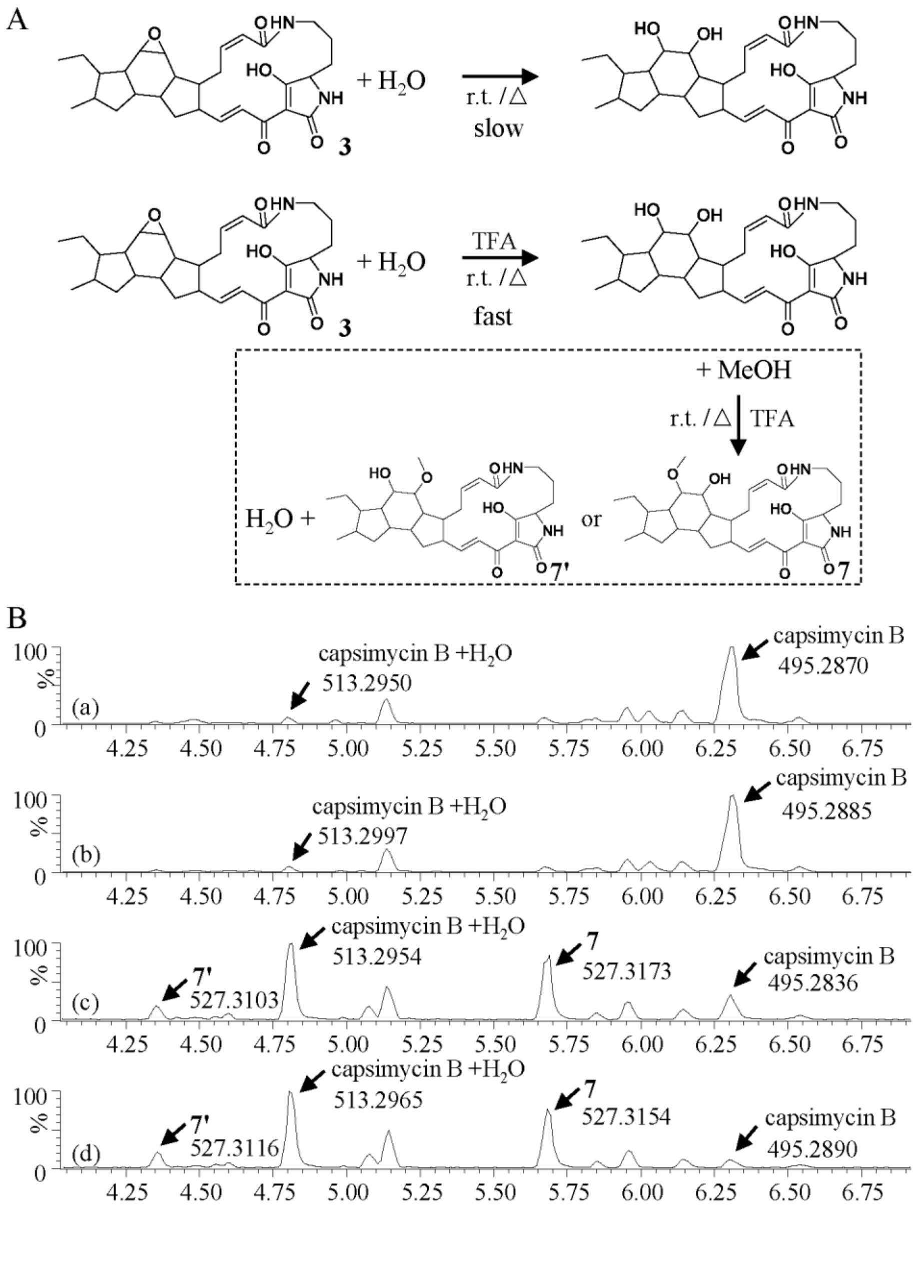

Figure S79. Gel filtration of purified IkaD indicated with the blue line.

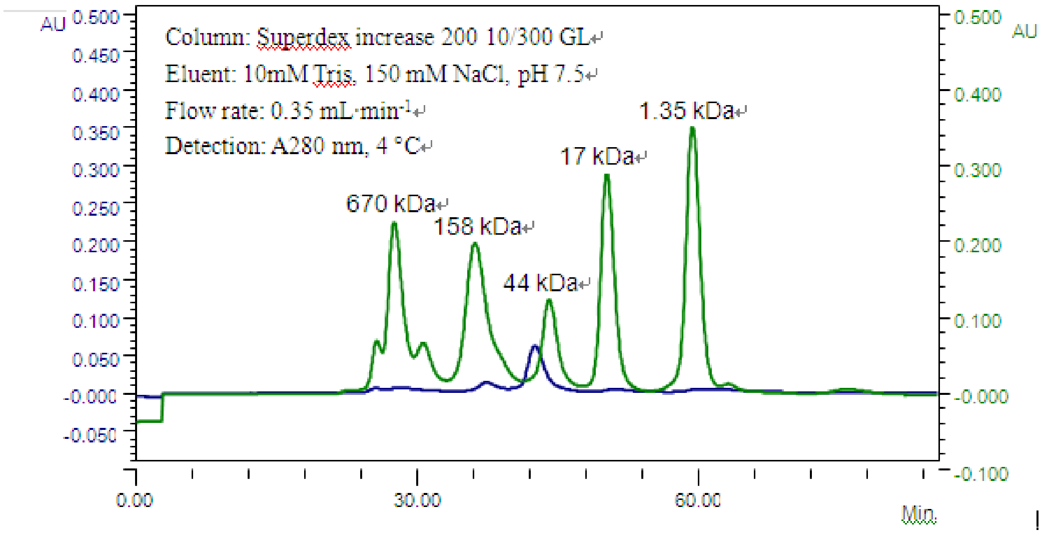

Gel filtration of purified IkaD. The standards (Bio-Red, USA) are shown in green and are as follows: 1.35 kDa (Vitamin B12), 17 kDa (Myoglobin from horse), 44 kDa (Ovalbumin from chicken), 158 kDa (γ-globulin from bovine) and 670 kDa (Thyroglobulin from bovine). The molecular mass of the recombinant P450 monooxygenase IkaD was determined by analytical gel filtration on Biologic DuoFlowTM Chromatography System (Bio-Rad, USA), column using Tris-HCl (10mM Tris, 150 mM NaCl, pH 7.5) buffer.

Figure S80. IkaD identification by nano LC-MS/MS and database search

MASCOT Search Results

User : hou
E-mail :
Search title : maXis_manual
MS data file : 281474976714999.mgf
Database : Trembl 2016_01 (59,718,159 sequences; 19,944,314,533 residues)
Taxonomy : Bacteria (Eubacteria) (37,204,640 sequences)
Timestamp : 10 Aug 2016 at 05:38:07 GMT

Re-search ◉ All ○ Non-significant ○ Unassigned [help] Export As XML

Not what you expected? Try the select summary.

▼Search parameters

Type of search : MS/MS Ion Search
Enzyme : Trypsin
Fixed modifications : Carbamidomethyl (C)
Variable modifications : Oxidation (M)
Mass values : Monoisotopic
Protein mass : Unrestricted
Peptide mass tolerance : ± 20 ppm (# ^{13}C = 2)
Fragment mass tolerance : ± 0.05 Da
Max missed cleavages : 2
Instrument type : ESI-QUAD-TOF
Number of queries : 7,741

▼Score distribution

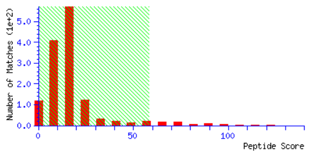

Peptide score distribution. Ions score is −10log(P), where P is the probability that the observed match is a random event. Individual ions scores **> 58** indicate **identity** or **extensive homology** (p<0.05).

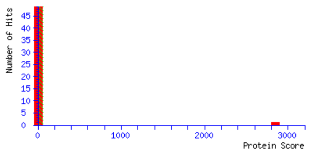

[Deprecated] Score distribution for family members in the first 50 proteins. Protein scores are derived from ions scores as a non-probabilistic basis for ranking protein families.

▼Legend

Dupes	Expect	Rank	U	1	2	Peptide	
	0.037	▶2				GAYSLSLR	significant
	9	▶1				GFFLFVEGGR	top ranking
	6.4e-005	▶1				GSSIFGLAPGK	significant and top ranking
	1.3e-006	▶1		■		SSGTSYPDVLK	peptide is found in all proteins in family member 1
	6.2e-007	▶1			■	VCNYVSWIK	peptide is found in some but not all proteins in family member 2
	6.4e-005	▶1	U			GSSIFGLAPGK	unique
▶2	5.7e-005	▶1				LNTLETEEWFFK	peptide has two duplicates
	0.18	▶1				*LNTLETEEWFFK*	duplicate peptide

Right-facing triangle (▶) in the Dupes or Rank column indicates content that can be expanded by clicking on it. Down-facing triangle (▼) indicates the content is expanded and can be collapsed.
For more details about particular columns, see results format help.

Protein Family Summary

Filter | Significance threshold p< 0.05 | Max. number of families AUTO | [help]
Ions score or expect cut-off 15.0 | Dendrograms cut at 0
Preferred taxonomy All entries

Proteins (6) | Report Builder | Unassigned (7609) | § permalink

Protein families 1–6 (out of 6)

10 per page 1 Expand all Collapse all

Accession contains Find

▼1 **A0A0F7FYZ9** 2847 Unspecific monooxygenase OS=Streptomyces xiamenensis GN=SXIM_40690 PE=3 SV=1

		Score	Mass	Matches	Sequences	emPAI	
**1.1**	A0A0F7FYZ9	2847	46024	131 (100)	22 (18)	6.44	Unspecific monooxygenase OS=Streptomyces xiamenensis GN=SXIM_40690 PE=3 SV=1

▼131 peptide matches (38 non-duplicate, 93 duplicate)

☑ Auto-fit to window

Query	Dupes	Observed	Mr(expt)	Mr(calc)	ppm	M	Score	Expect	Rank	U	Peptide
4386	▶3	516.7761	1031.5376	1031.5287	8.55	0	57	0.071	▶1	U	R.FGVPENELK.W
5104		673.8522	1345.6899	1345.7030	-9.74	1	40	0.038	▶1	U	R.FGVPENELKWK.Q
5109		449.5746	1345.7020	1345.7030	-0.74	1	30	0.12	▶1	U	R.FGVPENELKWK.Q
5144	▶6	452.9169	1355.7289	1355.7350	-4.53	0	65	0.0008	▶1	U	R.HVPILSGFSFPR.Y
5205	▶1	678.8746	1355.7347	1355.7350	-0.22	0	100	5.1e-007	▶1	U	R.HVPILSGFSFPR.Y
5418	▶1	683.8495	1365.6844	1365.6928	-6.16	0	92	1.4e-006	▶1	U	R.LPYGDDAYLAIR.Y
5543	▶2	709.3669	1416.7192	1416.7361	-11.9	0	122	1.3e-008	▶1	U	R.FSIVASLGQDQPR.T
5687		718.9174	1435.8202	1435.8259	-4.00	1	45	0.18	▶1	U	R.RGEAVIPVIAAANR.D
5690	▶1	479.6157	1435.8253	1435.8259	-0.40	1	80	0.00014	▶1	U	R.RGEAVIPVIAAANR.D
5833	▶5	749.9245	1497.8344	1497.8402	-3.87	0	108	7.1e-007	▶1	U	R.VELQVVLEALTER.F
5886	▶1	500.2875	1497.8407	1497.8402	0.30	0	80	6.9e-005	▶1	U	R.VELQVVLEALTER.F
6005	▶1	503.5863	1507.7370	1507.7276	6.26	0	59	0.0084	▶1	U	R.ARPTDDMFGMLVR.A
6046	▶2	761.8875	1521.7605	1521.7675	-4.61	0	123	3.1e-008	▶1	U	R.YATEDLELGGVTVR.R
6054	▶1	508.2615	1521.7627	1521.7675	-3.15	0	56	0.0078	▶1	U	R.YATEDLELGGVTVR.R
6253		508.9137	1523.7192	1523.7225	-2.17	0	51	0.0037	▶1	U	R.ARPTDDMFGMLVR.A + Oxidation (M)
6259		508.9170	1523.7292	1523.7225	4.44	0	26	0.28	▶1	U	R.ARPTDDMFGMLVR.A + Oxidation (M)
6422		811.4355	1620.8564	1620.8624	-3.67	1	71	0.00015	▶1	U	K.VRLPYGDDAYLAIR.Y
6426	▶2	541.2968	1620.8686	1620.8624	3.85	1	102	4.2e-006	▶1	U	K.VRLPYGDDAYLAIR.Y
6432	▶17	541.9420	1622.8042	1622.8165	-7.57	0	75	1.1e-005	▶1	U	R.VGNGLFSLDPPQHSR.L
6434	▶4	812.4099	1622.8051	1622.8165	-7.00	0	76	1.7e-005	▶1	U	R.VGNGLFSLDPPQHSR.L
6521	▶1	555.6119	1663.8138	1663.8287	-8.93	1	43	0.037	▶1	U	R.RARPTDDMFGMLVR.A
6523		832.9165	1663.8185	1663.8287	-6.13	1	44	0.019	▶1	U	R.RARPTDDMFGMLVR.A
6559		560.2891	1677.8455	1677.8686	-13.7	1	22	0.22	▶1	U	R.YATEDLELGGVTVRR.G

▶1 subset or intersection (1 subset protein in total)

Protein identification by nano LC-MS/MS and database search

IkaD was reduced by 1M DTT at 60 °C for 30 min and cysteine residues were blocked by 1M IAM for 20 min at room temperate. Protein was digested with Sequencing Grade Modified Trypsin (Promega, USA) via the FASP protocol with spin ultrafiltration units of nominal molecular weight limit of 10,000 Da. After digestion, the peptides were collected by centrifugation and the filtration units were washed with 50 mM NH_4HCO_3. Then the pooled peptides were dried by SpeedVac and re-dissolved in 2% ACN with 0.1% formic acid. Each of the fractions was performed using an LC system (Nano Pump, Ultimate 3000, Dionex, Thermofisher) equipped with an ESI-Q-TOF mass spectrometer (maXis, Impact, Bruker Daltonik, Germany). Briefly, the peptides were loaded onto a peptide trap column (100 µm × 2 cm, 5 µm, Dionex, Thermofisher) for desalination and concentration with 2% ACN and 0.1% formic acid at a flow rate of 5 µL/min for 10 min. Trapped peptides were released and separated in a C18 capillary column (75 µm × 15 cm, 3 µm, Dionex, Thermofisher). The peptides were eluted with a gradient of 4-80% ACN/H_2O hold for 50 min at a constant flow rate of 400 nL/min. The mass spectrometer was performed in data dependent acquisition mode (m/z 350-1500) using a full MS scan followed by ten MS/MS scans on the ten most intense ions from the MS spectrum. Tandem mass spectra were processed with Compass Data Analysis (version 4.1) according to the standard workflow. The peak list was directly generated from raw data using centroid algorithm with peak width set as 0.1 m/z and intensity above 100, and submitted for database search using Mascot (version 2.4, Matrix Science) to identify the protein from the Trembl_Bacteria database (37,204,640 sequences). No peak smooth or filter process was applied. Carbamidomethyl of cysteine was specified as fixed modifications (C) and oxidation of methionine was specified as variable modification (M). Two missed cleavages were allowed to trypsin. Peptide mass tolerance was set to 20 ppm and fragment mass tolerance to 0.8 Da. Peptide charges of +2, +3, and +4 were selected. The criteria of two peptides and a significance threshold $p < 0.05$ were used for peptide identification.

Figure S81. Expended NOESY spectra (600 MHz, MeOD/$CDCl_3$) of **4** (A-C) and expended ROESY spectra (600 MHz, MeOH/$CDCl_3$) of **5** (D-E).

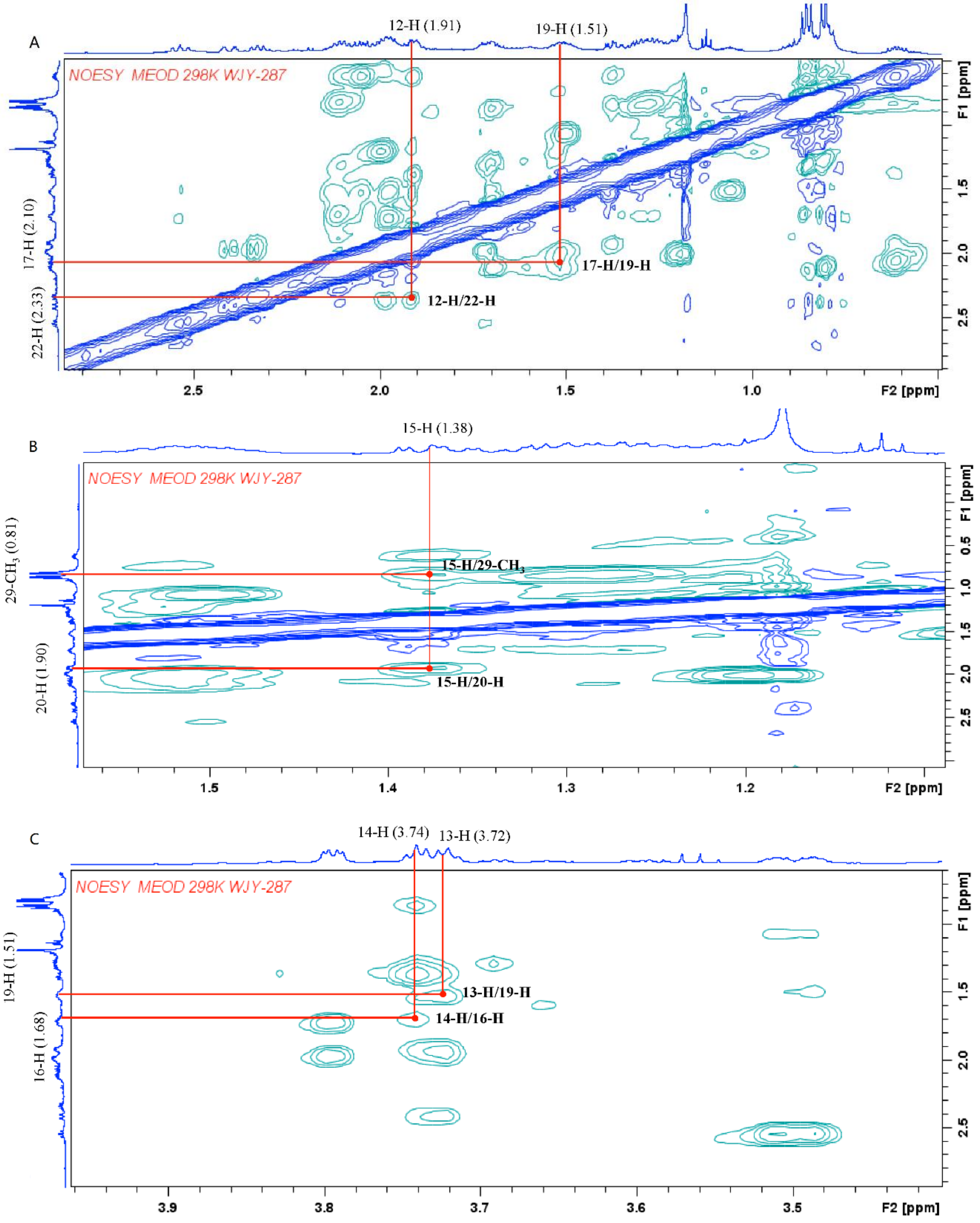

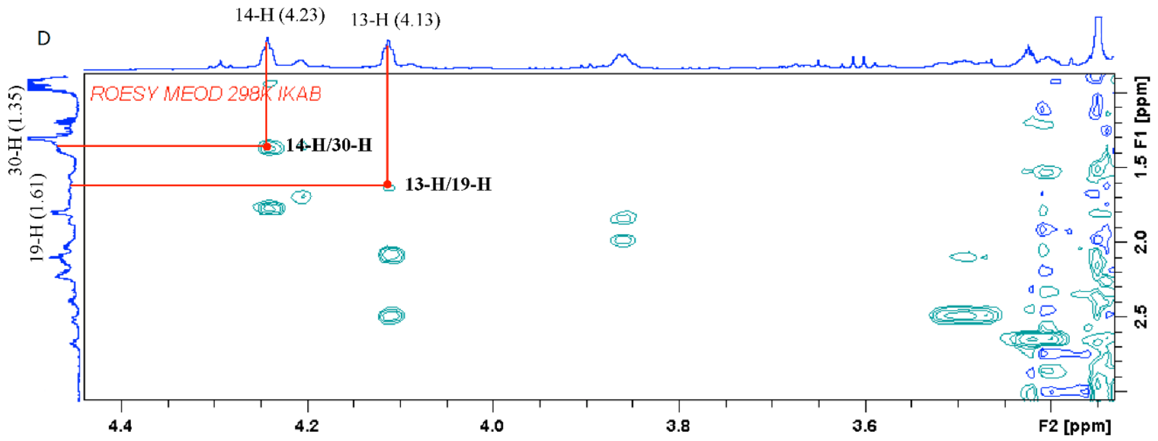
D
14-H (4.23)
13-H (4.13)
ROESY MEOD 298K IKAB
30-H (1.35)
19-H (1.61)
14-H/30-H
13-H/19-H
F1 [ppm]
F2 [ppm]

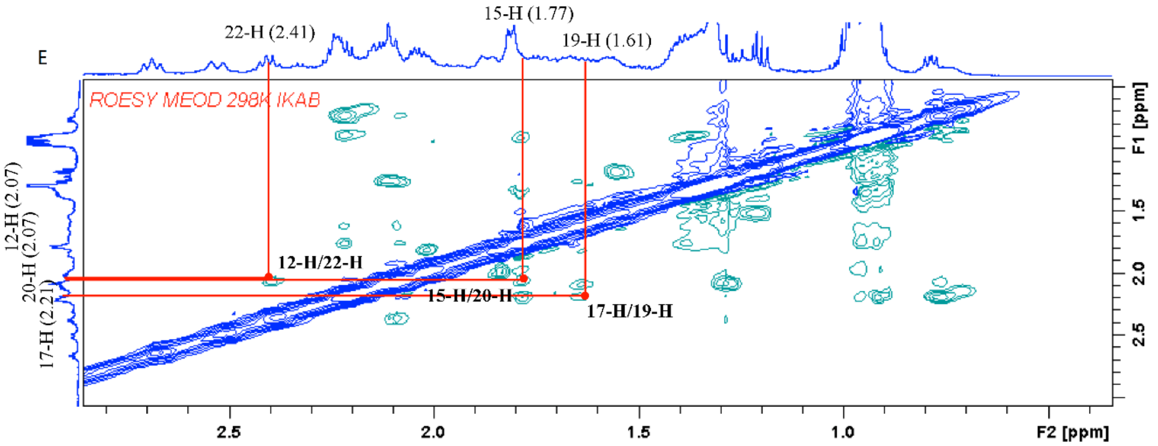
E
22-H (2.41)
15-H (1.77)
19-H (1.61)
ROESY MEOD 298K IKAB
12-H (2.07)
20-H (2.07)
17-H (2.21)
12-H/22-H
15-H/20-H
17-H/19-H
F1 [ppm]
F2 [ppm]
